# NAD^+^ supply and redox state limit developmental speed in the *Drosophila* eye

**DOI:** 10.1038/s44318-026-00801-4

**Published:** 2026-05-13

**Authors:** Nisha Veits, Yuting Guo, Jingjing He, Khalil Mazouni, Ivan Nemazanyy, Martin Bres, Cara Picciotto, Claire Mestdagh, Yan Yan, Francois Schweisguth

**Affiliations:** 1 Institut Pasteur, Université Paris Cité, 4D Unit, Paris, France; 2 CNRS UMR3738, Paris, France; 3 Shenzhen PKU-HKUST Medical Center; Division of Life Science, Hong Kong University of Science and Technology, Hong Kong, China; 4 Platform for Metabolic Analyses, INSERM US24/CNRS UMS 3633, Paris, France

**Keywords:** Development, Metabolism

## Abstract

The cellular and biochemical processes that define the speed at which embryos develop, tissues form, and cells differentiate remain largely unknown. Using the speed of progression of a differentiation front in the developing *Drosophila* eye to measure developmental speed, we identified genetic perturbations that slowed the progression of this front. Inhibiting the electron transport chain (ETC), and more generally energy production in mitochondria, resulted in reduced developmental speed. Defective ETC activity led to increased NADH/NAD^+^ ratio, whereas ATP levels remained constant due to a compensatory increase in glycolysis. Targeted perturbations showed that the metabolic state of the cells ahead of and/or at the differentiation front determined its speed. Genetic and diet-based perturbations of NAD^+^ metabolism indicated that developmental speed was limited by NAD^+^ availability. Thus, developmental speed appeared constrained by the cellular redox state and the demand for NAD^+^ in the developing *Drosophila* eye. Our findings therefore show that the NADH/NAD^+^ ratio is key to regulating developmental speed and highlight the importance of NAD^+^ availability for this regulation in *Drosophila*.

## Synopsis

During development, tissues in individuals of the same species in given environmental conditions differentiate at the same rate; however, how this tempo is determined is not well understood. This study identifies the NADH/NAD^+^ ratio and NAD^+^ availability as key determinants of developmental speed in the developing *Drosophila* eye.

- The speed of progression of the differentiation front of the eye was used to study the effects of gene-based perturbations on developmental speed using metabolomic, transcriptomic and biosensor analyses.
- Loss of the mitochondrial electron transport chain activity or of NAD^+^ recycling led to reduced developmental speed in the eye.
- Genetic and diet-based perturbations that change developmental speed converge on NAD^+^ metabolism and altered NADH/NAD^+^ ratio.

## Abstract (web summary)

The rate of cell differentiation in the *Drosophila* eye depends on the mitochondrial electron transport chain activity and NADH/NAD^+^ ratio, and is limited by the demand for NAD^+^.

## Graphical abstract

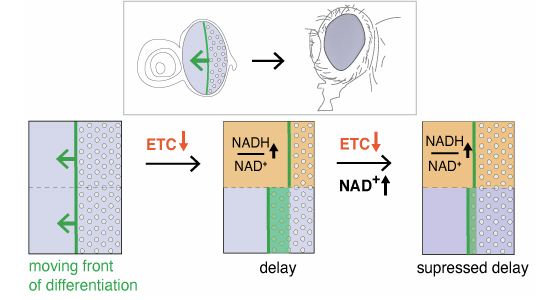

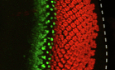

## Introduction

Time is an inherent property of living systems, and many biological processes have defined time scales. For instance, the one-cell embryo of *Drosophila* produces a complex larva within a single day at 25 °C. However, while it is commonly observed that embryos and tissues develop at a reproducible tempo for defined environmental conditions, little is known about what sets the speed at which ordered, interacting, and non-reversible steps occur during development (Ebisuya and Briscoe, 2018). At the organismal level, developmental speed can be influenced by nutrition and temperature (Gillooly et al, 2002), indicating that metabolism constrains and influences the speed of development (Ghosh et al, 2023). Indeed, differences in mitochondrial activity, energy metabolism, protein stability and/or gene expression were recently shown to underly the species-specific differences in developmental speed between different mammalian embryos (Diaz-Cuadros et al, 2023; Iwata et al, 2023; Lázaro et al, 2023; Matsuda et al, 2020; Matsuda et al, 2025; Nakanoh et al, 2024; Porte et al, 2024; Rayon et al, 2020). Notably, the inhibition of the mitochondrial electron transport chain (ETC) was shown to reduce the speed of the segmentation clock in an ex vivo stem cell-based assay mimicking somitogenesis in the mouse (Diaz-Cuadros et al, 2023). The ETC uses the energy produced by the transfer of electrons from donors, e.g., reduced nicotinamide dinucleotide (NADH), to acceptors to pump protons in the intermembrane space of mitochondria, thereby generating a proton gradient that drives the production of adenosine triphosphate (ATP). Since the first protein complex of the ETC, complex I (cI) oxidizes NADH, inhibiting its activity increases the NADH/NAD^+^ ratio, affecting in turn the activity of many enzymes regulating catabolism and anabolism. Since various perturbations changing the NADH/NAD^+^ ratio altered the rate of somitogenesis in this ex vivo assay, developmental speed was proposed to be regulated by the NADH/NAD^+^ ratio in this context (Diaz-Cuadros et al, 2023). Beyond cellular redox state, other processes have been proposed to modulate developmental speed in mammalian embryos (Diaz-Cuadros et al, 2023; Iwata et al, 2023; Lázaro et al, 2023; Matsuda et al, 2020; Matsuda et al, 2025; Nakanoh et al, 2024; Porte et al, 2024; Rayon et al, 2020), raising the possibility that distinct molecular processes contribute to various extents in the regulation of developmental speed in different tissues and/or species (Matsuda et al, 2025).

**Figure 1:**
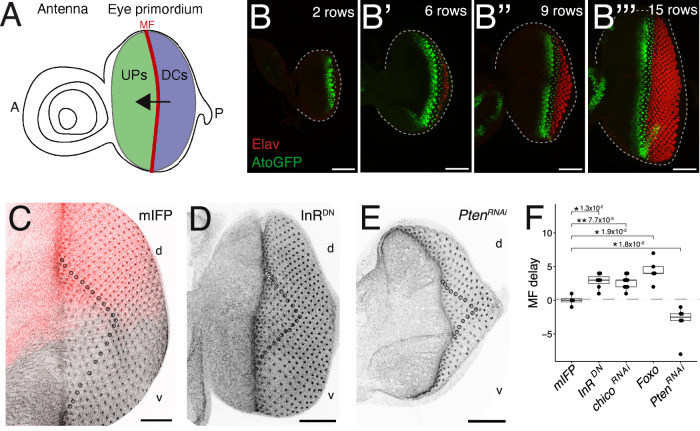
A simple assay for front progression. (**A**) Schematic of an eye-antenna imaginal disc. The MF (red) travels through the eye primordium from posterior (P) to anterior (**A**; arrow) as Undifferentiated Progenitors (UPs; green) become Differentiated Cells (DCs; blue). (**B**–**B”’**) Time series of eye discs stained for Atonal (AtoGFP, green) marking the MF, and Elav (red) marking DCs. The number of ommatidial rows was used as a proxy of developmental age. Note that disc growth correlates with MF progression (eye disc outlined by a white dotted line). Scale bars, 50 µm. (**C**–**E**) Mapping time in space: ommatidia identified using Ecad (black) were counted (circles) from the midline to the MF in dorsal (*d*) and ventral (*v*) compartments in *mirr* > *mIFP* (C; *mIFP*, red, marked *d* cells), *mirr* > *InR*^*DN*^ (**D**), and *mirr>Pten*^*RNAi*^ (**E**) eye discs. Scale bars, 50 µm. (**F**) Relative changes in MF progression, measured as the difference in row numbers in the *v* and *d* compartments, upon expression of InR^DN^ (2.9 + /−1.0, *n* = 6), *chico*^*RNAi*^ (4.4 + /−1.8, *n* = 7), Foxo (2.7 + /−1.0, *n* = 5), and *Pten*^*RNAi*^ (−3.2 + /−2.8, *n* = 6). Expression of mIFP (0.0 + /−0.8, *n* = 4) served as a negative control. While InR^DN^, *chico*^*RNAi*^, and Foxo led to a MF delay, *Pten*^RNAi^ resulted in increased MF progression. The box plots show the median and the interquartile range from the 25th to 75th percentile; whiskers extend to data points within 1.5× the interquartile range of the lower and upper quartiles, representing the approximate minimum and maximum non-outlier values. Wilcoxon test, *P* values: *<0.05; **<0.01. Source data are available online for this figure.

The *Drosophila* eye is an attractive model system to study developmental speed. The adult fly eye comprises ~750 light-receiving multicellular units, or ommatidia, that are arranged in a crystal-like array. It develops from an eye primordium in the eye-antenna imaginal disc (Ready et al, 1976). This eye primordium differentiates in a progressive manner as a moving differentiation front, forming a morphogenetic furrow (MF), sweeps through the disc epithelium from posterior to anterior over a 2.5-day period to produce ~30 rows of regularly spaced ommatidia (Roignant and Treisman, 2009) (Fig. 1A–B”’). Cells located anterior to the front remain undifferentiated and proliferate, whereas cells posterior to the furrow differentiate and form ommatidia. This front moves at a relatively constant speed of ~0.6 rows per hour (h) (Spratford and Kumar, 2015; Tsao et al, 2016), or ~3 μm/hr (Wartlick et al, 2014), with a new row of ommatidia produced at each pulse of Atonal, the proneural transcription factor that controls eye differentiation (Couturier et al, 2026; Jarman et al, 1994). A self-propagating mechanism involving signals produced by differentiating cells underlies the progression of the front (Couturier et al, 2026; Greenwood and Struhl, 1999; Ma et al, 1993; Roignant and Treisman, 2009). Thus, the progression speed of the differentiation front can be used as a proxy to study the speed of developmental patterning. This system offers key experimental advantages. First, the temporal progression of patterning and differentiation can be easily mapped in space. It can therefore be studied in fixed tissues. Second, the molecular mechanisms underlying the progression of the front are well-understood. Third, genetic tools allow for targeted perturbations of developmental speed in the eye. Indeed, compartment-specific perturbations make it possible to study relative changes in developmental speed within a single eye disc, thereby overcoming the systemic influence of extrinsic factors, such as temperature, nutrition, feeding behavior, infection, and other stresses on developmental speed. In this approach, the unperturbed compartment of the disc can be used as an internal control for any physiological change acting at the organismal level. Thus, the fly eye appears as a good model system to study developmental speed at the tissue level in the context of the living organism.

Here, we used a simple in vivo assay to identify RNAi-based perturbations that affected the timely progression of the MF. This approach showed that the mitochondrial ETC and Oxidative Phosphorylation (OxPhos) activities were required for the proper progression of the differentiation front. Using fluorescent metabolic sensors and metabolomic approaches, we found that the loss of ETC activity increased the NADH/NAD^+^ ratio but did not detectably change the level of ATP. ATP levels were maintained by increased glycolysis upon loss of ETC activity, which was associated with increased *Lactate dehydrogenase* (*Ldh*) gene expression, likely to promote NAD^+^ regeneration. Indeed, expression of a heterologous NADH oxidase and addition of NAD^+^ precursors in the diet indicated that the rate of NAD^+^ regeneration from NADH appeared to limit the rate of progression of the MF upon loss of ETC activity. Moreover, the biosynthesis of NAD^+^ by the salvage pathway was found to be important for proper developmental speed in the eye, and while changes in developmental speed often correlated with changes in NADH/NAD^+^ ratio, exceptions were noted in cells for which energy metabolism played a key role in the progression speed of the front. Our data therefore indicated that the demand for NAD^+^ constrained developmental speed in the developing fly eye.

## Results

### An assay for developmental speed in the eye

The front of differentiation moves at a relatively constant rate, with a new row of ommatidia forming every ~100 min at 25 °C (Spratford and Kumar, 2015; Tsao et al, 2016) (Fig. 1A–B”’). What determines this speed is not known. Since the speed of development at the organismal level varies with systemic variations in circulating nutrients, hormones, and other secreted factors, the speed of progression of the MF should vary with nutrition, stress, social interactions, and other environmental factors (Cassidy et al, 2019). This variability therefore complicates the analysis of the progression speed of the MF at the population level. To overcome this issue, we studied the relative differences in speed within individual eye discs by measuring the relative progression of the MF in experimental dorsal (*d*) cells expressing the *mirror* (*mirr*) *Gal4* driver relative to control ventral (*v*) cells. A difference in progression speed was inferred by counting the number of ommatidial rows produced over a given time in the *v* and *d* compartments of the same disc (Fig. 1C). Scoring fewer rows of ommatidia in *d* cells relative to *v* cells would indicate that the progression of the MF is delayed in the *d* compartment, whereas scoring more rows would indicate an increase in speed. Thus, this assay does not measure absolute speed and may rather reveal relative differences in speed. In addition, this simple assay should be faster than population-based assays used earlier (Spratford and Kumar, 2015; Tsao et al, 2016; Wartlick et al, 2014), therefore allowing us to test many genetic perturbations.

**Figure EV1:**
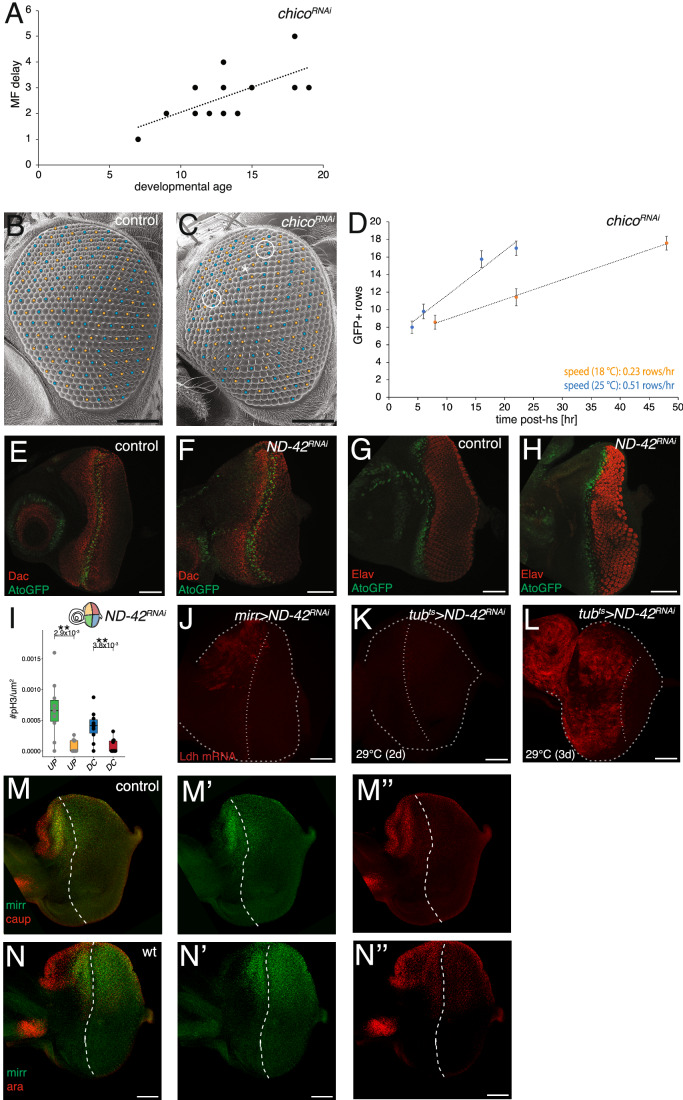
Patterning speed analysis, conditional silencing of the *ND-42* gene, and co-expression of the *ara*, *caup*, and *mirr* genes. (**A**) The MF delay in *mirr>chico*^*RNAi*^ eye discs increased with developmental age, as determined here using the total number of rows measured in the *v* compartment (the regression line is shown; *R*^2^ = 0.5). (**B**, **C**) Scanning Electron Microscopy (SEM) images of adult fly eyes. A regular crystal-like array of ommatidia was observed in control (**A**), whereas minor defects suggestive of interrupted rows were seen in the dorsal part of the eye in *mirr>*c*hico*^*RNAi*^ flies (**B**; white circles indicate where non-consecutive rows appeared to merge; the star indicates a partial interruption; orange and blue dots provide a visualization tool to follow the regular rows of ommatidia). (**D**) The speed of progression of the MF was measured in control *mirr* > *mIFP* discs from larvae grown at 18 °C (orange) and 25 °C (blue) using a recombination-based RFP-to-GFP switch. The MF moved twice as slowly at 18 °C (0.23 rows/h, *n* = 32) than at 25 °C (0.51 rows/h, *n* = 22). Error bars show standard deviation. (**E**–**H**) Dachsund (Dac, red in (**E**, **F**)) and Elav (red, in (**G**, **H**)) showed similar patterns of expression in control *mirr-Gal4 ato*^*GFP*^ (**D**, **F**) and *mirr* > *ND-42*^*RNAi*^ *ato*^*GFP*^ eye discs (**E**, **G**). AtoGPP (green) marked the position of the differentiation front. Dac is a transcription cofactor expressed in a patterned manner along the AP axis, and Elav is an RNA-binding protein expressed in photoreceptors. Dac and Elav are used here as AP patterning markers. (**I**) The silencing of *ND-42* led to a decrease in proliferation (number of pH3-positive cells per surface area) in UPs and to a loss of the SMW (plotted as DCs, *n* = 9). (**J**–**L**) To conditionally silence the *ND-42* gene in all eye disc cells, we used a *tub-Gal4 tub-Gal80*^*ts*^ driver (*tub*^*ts*^) and grew larvae at a restrictive temperature (29 °C). To monitor the downregulation of *ND-42* gene activity, we used *Ldh* mRNA accumulation as a reporter for reduced *ND-42* activity (see Fig. 5I–J”). We observed strong *Ldh* expression in UPs, peripodial, and antenna cells after 3 days (3 d) of conditional knockdown of *ND-42* (**K**). This effect appeared to be stronger than the one observed using *mirr-Gal4* (**J**). The *Ldh* signal was very weak after 2 days at 29 °C (**L**). The position of the MF was indicated by a white dotted line (disc outlines, gray dotted lines). (**M**, **N**) Expression pattern of the *ara* (red in (**N**, **N”**)), *caup* (red in (**M**, **M”**)) and *mirr* genes (green in (**M**–**N’**)) showing that all three genes were largely co-expressed in *d* eye field and peripodial cells (MF position indicated by white dotted line). The box plots show the median and the interquartile range from the 25th to 75th percentile; whiskers extend to data points within 1.5× the interquartile range of the lower and upper quartiles, representing the approximate minimum and maximum non-outlier values. Wilcoxon test, *P* values: **<0.01. Scale bars, 50 µm (**E**–**N”**) and 100 µm (**B**, **C**).

To validate this assay, we tested the Insulin signaling pathway that is known to regulate cell growth, cell proliferation, and differentiation timing in the *Drosophila* eye (McNeill et al, 2008; Cassidy et al, 2019). Using a dominant-negative (DN) form of the Insulin receptor (InR), RNAi-mediated silencing of a conserved and essential InR substrate (Chico), as well as the overexpression of Foxo, a transcription factor inhibited by InR signaling, we found that lowering Insulin signaling activity delayed the progression of the MF (Fig. 1D,F; Dataset EV1). In contrast, increasing InR signaling by silencing the Phosphatase and TENsin homolog (Pten) led to increased progression of the MF (Fig. 1E,F; Dataset EV1). These results indicated that developmental speed in the eye positively correlates with InR signaling, hence validating our simple assay. We further noticed that the delay in the progression of the MF increased with developmental age in *chico*^*RNAi*^ discs (Fig. EV1A), consistent with a speed phenotype. Thus, to minimize the variability in MF delay values measured in our screen, we scored eye discs from larvae showing 20 + /−10 rows of ommatidia in the *v* compartment. Finally, a delay in MF progression in larvae did not produce an easily scorable phenotype in the adult eye (Fig. EV1B,C), implying that developmental speed defects cannot be easily screened in adult eyes. In summary, since scoring a small number of eye discs was sufficient to detect a statistically significant difference in MF progression, this simple assay should in principle allow us to identify perturbations that reduce or increase the rate of development.

### Delayed progression of the MF upon energy metabolism perturbations

**Figure 2:**
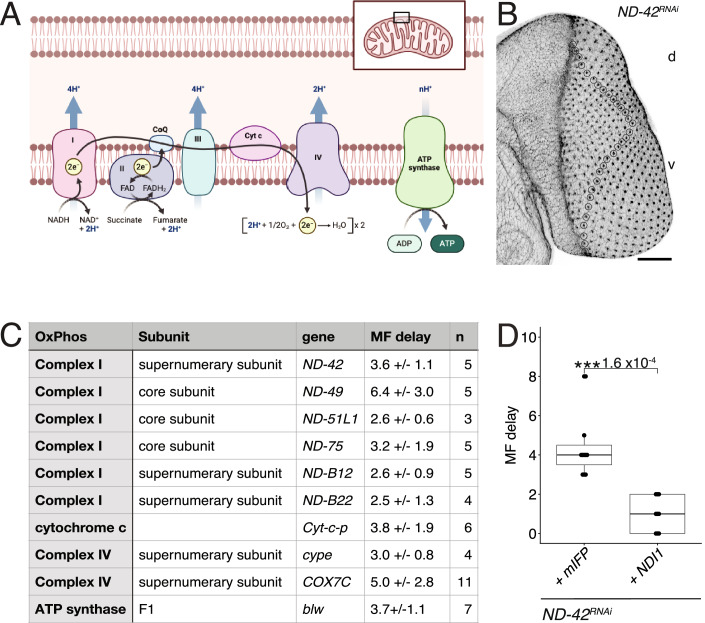
Delayed progression of the front upon ETC inhibition. (**A**) Schematic of the ETC (adapted from BioRender.com). (**B**) A *mirr* > *ND-42*^*RNAi*^ eye discs (Ecad, black) showing a difference in the number of ommatidial rows (circles), hence a delay in MF progression. Scale bar, 50 µm. (**C**) Results of the MF delay assay for selected ETC genes. (**D**) Expression of the yeast alternative NADH oxidase *NDI1* suppressed the *ND-42*^*RNAi*^ MF delay phenotype (*ND-42*^*RNAi*^ + *mIFP*, 4.2 + /−1.8, *n* = 11; *ND-42*^*RNAi*^ + *NDI1*, 1.0 + /−0.8, *n* = 9). Wilcoxon test, *P* values: ***<0.0001. Source data are available online for this figure.

We then used this assay to look for RNAi-based perturbations associated with altered developmental speed. To probe various aspects of cellular metabolism, including energy metabolism, stress responses, and biosynthesis and turnover of macromolecules, we selected and tested 166 genes (Dataset EV1). This screen identified several genes encoding subunits of the complexes cI, cIV, and cV of the ETC (Fig. 2A–C), including *ND-42*, a cI accessory subunit known as NDUFA10 in mammals (Kampjut and Sazanov, 2022; Wirth et al, 2016) and *bellwether* (*blw*), the alpha subunit of the mitochondrial F_0_F_1_-ATP synthase complex (cV). The MF delay seen upon *ND-42*^*RNAi*^ was suppressed by the expression of the yeast alternative NADH oxidase *NDI1* that was shown to bypass a loss of cI activity by restoring ETC activity downstream of cI (Cho et al, 2012; Sanz et al, 2010; Seo et al, 1998) (Fig. 2D). In addition, the RNAi-mediated silencing of the *Carnitine PalmitoylTransferase 2* (*CPT2*) and *pyruvate dehydrogenase* (*pdhb*) genes similarly delayed the progression of the MF (Dataset EV1), suggesting that fatty acid β-oxidation and production of Acetyl-CoA downstream of glycolysis were also important for the timely progression of the MF. In contrast, RNAi-mediated inhibition of many glycolytic and TCA enzymes showed little effect (Dataset EV1). Beyond energy metabolism, a few enzymes involved in other metabolic pathways scored positively in our assay (Dataset EV1). However, no perturbation associated with increased furrow progression was identified (beyond *Pten*^*RNAi*^ and a constitutively active *myr-Akt*; see Fig. 1E and Dataset EV1, respectively). In sum, our results strongly indicated that energy production by mitochondria is key for the timely progression of the MF in *Drosophila*.

### Reduced speed of front progression upon cI inhibition

**Figure 3:**
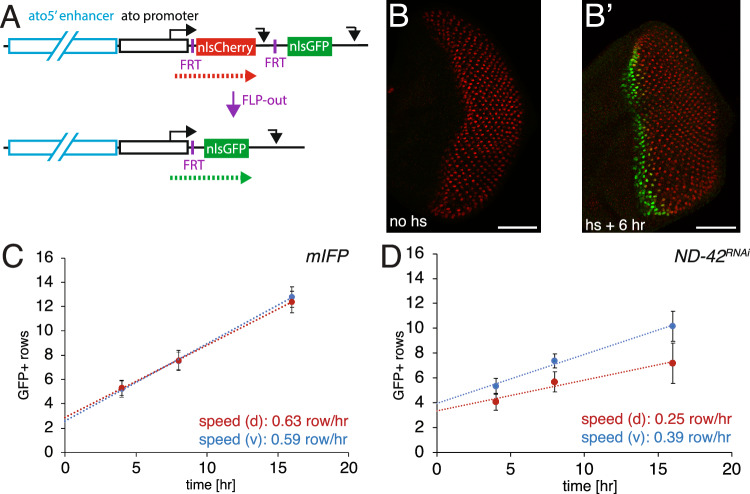
Reduced speed of progression of the front upon ETC inhibition. (**A**–**B’**) Recombination-based strategy to infer MF speed from fixed samples (**A**). The Flp-mediated excision of nuclear mCherry upon heat-shock (hs) triggers a mCherry-to-GFP switch in gene expression. The *ato5’* enhancer directs the expression of mCherry (or GFP) at the front. Prior to recombination, mCherry produced at the front was stable enough to mark all ommatidia (**B**). Following recombination, new rows of GFP-positive/mCherry-negative ommatidia are produced (**B’**). The speed of progression of the front was inferred by scoring the number of GFP-positive rows produced in the time interval from hs to fixation. Scale bars, 50 µm. (**C**, **D**) The speed of the front was determined as the slope of the regression line obtained from plotting the number of GFP-positive rows scored at 4, 8, and 16 h after hs. In control *mirr* > *mIFP* discs, the measured speed was ~0.6 rows/h (4 h, *n* = 44; 8 h, *n* = 25; 16 h, *n* = 37). No speed difference was observed between *d* and *v* cells in control discs (**C**). In *mirr* > *ND-42*^*RNAi*^ discs (**D**), the front progressed at a slower speed in *d* cells (0.25 rows/h) relative to control *v* cells (0.39 rows/h; 4 h, *n* = 31; 8 h, *n* = 29; 16 h, *n* = 32). Error bars indicate standard deviation. Source data are available online for this figure.

To further test whether the delayed progression of the MF resulted from reduced speed, we designed a recombination-based assay that allowed us to infer the speed of the differentiation front from fixed eye discs. In brief, a heat-induced recombination-based RFP-to-GFP switch followed by a chase period allowed us to score the number of GFP-positive rows produced in a defined amount of time (Fig. 3A,B). This assay was validated by showing that speed varied with temperature (Fig. EV1D). We next measured the average speed of the front in control larvae (Fig. 3C) and found that the MF moved at a speed of ~0.6 row/h, consistent with earlier reports (Spratford and Kumar, 2015; Tsao et al, 2016). Analysis of *mirr* > *ND-42*^*RNAi*^ discs showed that the silencing of the *ND-42* gene led to a ~30% reduction in relative speed (0.25 vs 0.39 row/hr in Fig. 3D). We therefore propose that the observed delay in MF progression likely resulted from a slow speed of the moving front of differentiation. Of note, the speed measured in control *v* cells of *mirr* > *ND-42*^*RNAi*^ discs (0.39 row/h) was lower than the speed measured in control larvae (~ 0.6 row/h), suggestive of a developmental delay in *mirr* > *ND-42*^*RNAi*^ larvae. We next addressed whether cI inhibition perturbed eye patterning and differentiation by studying the pattern of expression of key regulators of eye differentiation in *mirr* > *ND-42*^*RNAi*^ discs. We found that eye patterning remained largely unchanged upon cI inhibition (Fig. EV1E–H). However, we observed a significant reduction in cell proliferation, both anterior to the MF and at the position of the Second Mitotic Wave (SMW) (Kim et al, 2017), upon *ND-42* silencing (Fig. EV1I). We therefore conclude that cI inhibition slowed the progression of the MF and affected tissue growth but had no clear effect on fate patterning.

### NADH accumulation upon cI inhibition

**Figure 4:**
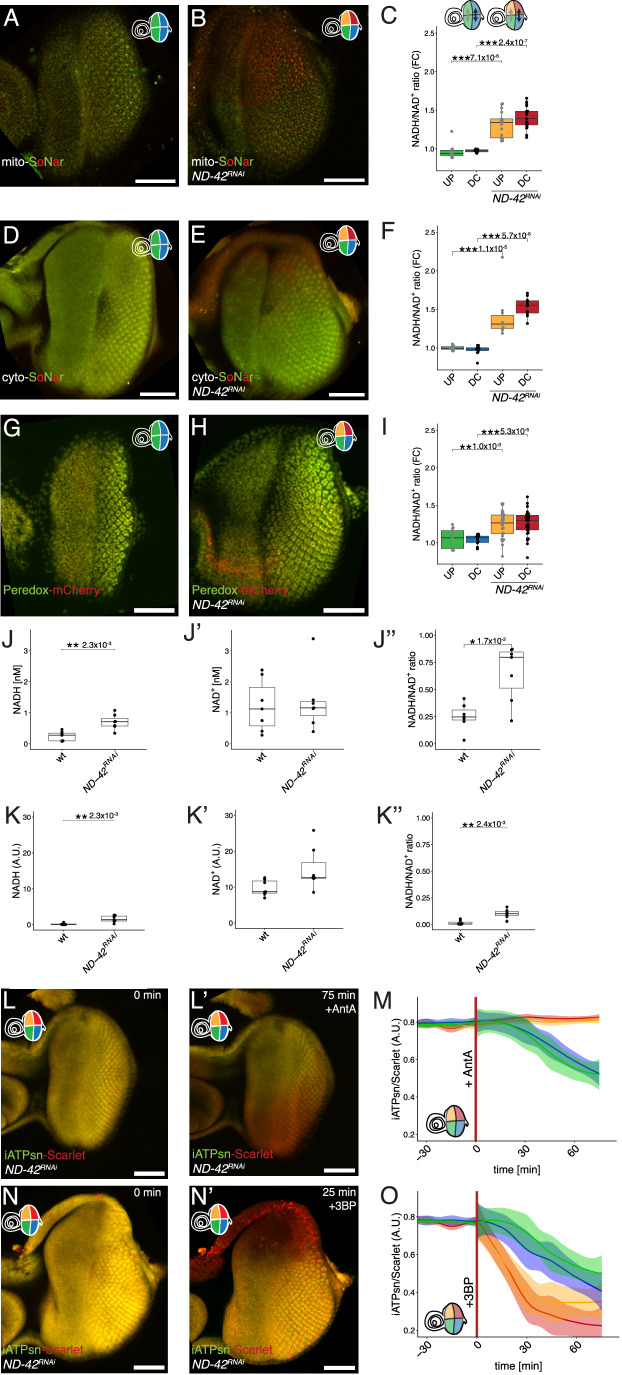
Glycolysis maintained ATP levels constant upon ETC inhibition. (**A**–**C**) Analysis of the mitochondrial NADH/NAD^+^ ratio in control *mirr* > *mIFP* (**A**, **C**) and *mirr* > *ND-42*^*RNAi*^ + *mIFP* discs (**B**, **C**). The *d* cells were identified using the infrared signal produced by mIFP (not shown). The color code used in (**C**) (and throughout the figures) is explained in the schematics shown in (**A**, **B**) (top): control UPs, green; control DCs, blue; *ND-42*^*RNAi*^ UPs, orange; *ND-42*^*RNAi*^ DCs, red. The mito-SoNar signals following excitation at 405 and 488 nm (in red and green in (**A**, **B**)) were used to measure the NADH/NAD^+^ ratio (**C**). Since similar ratio values were measured in *d* and *v* cells of control discs, the Fold Change (FC) between ratio values measured in *d* and *v* cells remained close to 1 (UPs: 0.97 + /−0.1; DCs: 0.97 + /−0.06, *n* = 10). In contrast, higher NADH/NAD^+^ ratio values were observed in dorsal UPs and DCs of *ND-42*^*RNAi*^ eye discs (UPs: 1.33 + /−0.16; DCs: 1.4 + /−0.15, *n* = 17), showing that cI inhibition resulted in increased NADH/NAD^+^ ratio in mitochondria. (**D**–**F**) Analysis of the cytosolic NADH/NAD^+^ ratio in control *mirr* > *mIFP* (**D**, **F**) and *mirr* > *ND-42*^*RNAi*^ + *mIFP* discs (**E**, **F**) using cyto-SoNar. The results are shown as in A-C for control (UPs: 0.97 + /−0.06; DCs 0.98 + /−0.07, *n* = 11) and *ND-42*^*RNAi*^ eye discs (UPs: 1.4 + /−0.29; DCs: 1.54 + /-0.12, *n* = 10). Inhibition of cI led to an elevated NADH/NAD^+^ ratio in the cytosol. (**G**–**I**) Analysis of the nuclear NADH/NAD^+^ ratio in control *mirr* > *mIFP* (**D**, **F**) and *mirr* > *ND-42*^*RNAi*^ + *mIFP* discs (**E**, **F**) using a ratiometric Peredox sensor (Peredox, green; mCherry, red). In control eye discs (*n* = 11), similar Peredox/mCherry ratio values were observed in UPs (1.05 + /0.13) and DCs (1.04 + /−0.06). In contrast, silencing the *ND-42* gene led to increased ratio values in both UPs (1.24 + /−0.17) and DCs (1.26 + /−0.17, *n* = 30), indicative of elevated NADH/NAD^+^ ratio upon cI inhibition. (**J**–**J”**) Analysis of NADH (**J**) and NAD^+^ levels (**J’**), and corresponding NADH/NAD^+^ ratio (**J”**), in whole cell lysates from control and *tub*^*ts*^ > *ND-42*^*RNAi*^ eye discs using a NAD^+^/NADH-glo assay (*n* = 7). The increased NADH/NAD^+^ ratio largely resulted from increased NADH. (**K**, **K”**) Quantification of NADH (**K**), NAD^+^ (**K’**), and corresponding NADH/NAD^+^ ratio (**K”**) in whole cell lysates of control *tub*^*ts*^ > *+* and *tub*^*ts*^ > *ND-42*^*RNAi*^ eye discs by LC/MS (*n* = 7). Increased NADH and NADH/NAD^+^ were observed (the ~twofold increase in NAD^+^ did not appear statistically significant). (**L**–**O**) Time-course analysis of ATP levels in *mirr* > *ND-42*^*RNAi*^ eye disc using a ratiometric ATP sensor (iATPsn, green; Scarlet, red in (**L**–**N’**)). Normalized intensity ratios are plotted in (**M**, **O**). Similar iATPsn/mScarlet ratios were observed in the *d* and *v* cells of *mirr* > *ND-42*^*RNAi*^ discs prior to adding the drug (**M**, *n* = 12; **O**, *n* = 9; the mean and standard deviation (s.d.) are shown), as seen also in snapshots taken at *t* = 0 (**L**, **N**; ~45 min after the onset of imaging). This indicated that the silencing of *ND-42* did not detectably affect the levels of ATP. However, addition of AntA, a cIII inhibitor, led to a rapid decrease in ATP in ventral UPs and DCs, showing that the ETC was active in these cells (**L’**, **M**). In contrast, AntA had no effect on *ND-42*^*RNAi*^ cells, indicating that ATP production did not rely on the ETC in these cells. Addition of 3BP, a glycolysis inhibitor showed that *ND-42*^*RNAi*^ cells, both UPs and DCs, were more sensitive than *v* control cells to glycolysis inhibition (**M**–**O**). This showed that the silencing of *ND-42* made UPs and DCs strongly dependent on glycolysis for ATP production. The box plots show the median and the interquartile range from the 25th to 75th percentile; whiskers extend to data points within 1.5× the interquartile range of the lower and upper quartiles, representing the approximate minimum and maximum non-outlier values. two-sided *t* test (**J**–**K”**) or Wilcoxon test (all other panels), *P* values: *<0.05; **<0.01; ***<0.0001. Scale bars, 50 µm. Source data are available online for this figure.

The cI is an oxidoreductase located at the inner membrane of mitochondria which accepts electrons from NADH and transfers them to ubiquinone (and eventually oxygen), thereby converting the produced free energy to pump protons across the inner membrane (Fig. 2A). Loss of cI activity therefore decreases the rate of NADH oxidation, leading to NADH accumulation. To examine whether cI inhibition altered the NADH/NAD^+^ ratio, we used the fluorescence sensor SoNar, which consists of an NADH/NAD^+^-binding domain fused to a circularized permuted YFP. The excitation spectra of SoNar vary with the relative binding of NADH *vs* NAD^+^ (Zhao et al, 2015). Flies expressing a mitochondria-targeted SoNar in all cells were generated and used to study the mitochondrial NADH/NAD^+^ ratio in ex vivo cultured eye discs. Similar SoNar signals were observed in *d* and *v* cells in control eye discs (Fig. 4A), in both Undifferentiated Progenitors (UPs, anterior to the furrow) and Differentiated Cells (DCs, posterior to the furrow), as shown by the *dv* Fold Change (FC) values which were close to 1 (Fig. 4A,C). In contrast, the silencing of the *ND-42* gene led to increased NADH/NAD^+^ ratio values in both UPs as well as DCs (Fig. 4B,C). Thus, as expected, cI inhibition led to an elevated NADH/NAD^+^ ratio in mitochondria.

Since the mitochondrial pools of NAD^+^ and NADH equilibrate with the cytosolic pools, in part via the activity of the Malate-Aspartate shuttle (Hu et al, 2021), we also generated a cytosolic SoNar. Similarly, the silencing of the *ND-42* gene resulted in increased NADH/NAD^+^ ratio values (Fig. 4D–F), showing that cI inhibition changed the NADH/NAD^+^ ratio in both mitochondria and cytosol.

To normalize the measured ratio values by the expression of the sensor, we next used the ratio metric sensor Peredox that consists of a circularly permuted variant of GFP fused to a NAD^+^/NADH-binding protein to monitor the ratio of free NADH over free NAD+ (Hung et al, 2011). Peredox was fused to the red fluorescent protein mCherry that served as an internal control for expression level. Since the cytoplasmic and nuclear pools of NAD^+^ and NADH are readily exchangeable (Cambronne et al, 2016), we used a nuclear version of Peredox-mCherry to report on the redox state of the cell (Diaz-Cuadros et al, 2023). Flies expressing nuclear Peredox-mCherry under the control of a ubiquitous promoter were generated and used to measure changes in the NADH/NAD^+^ ratio. Analysis of eye discs confirmed that cI inhibition resulted in increased NADH/NAD^+^ ratio (Fig. 4G–I).

We next quantified the levels of NAD^+^ and NADH in whole disc extracts using NAD^+^/NADH-Glo assay (Fig. 4J–J”) and Liquid Chromatography Mass Spectrometry (LC/MS) approaches (Fig. 4K–K”; Dataset EV2). In both assays, eye-antenna discs expressing the *ND-42*^*RNAi*^ construct in all cells using a *tub-Gal4 tub-Gal80*^*ts*^ driver were compared with control eye-antenna discs (Fig. EV1J,K). First, cells from control eye-antenna discs had more NAD^+^ than NADH (6x in Fig. 4J,J’; 10× in Fig. 4K,K’). Second, we confirmed that the silencing of the *ND-42* gene led to elevated NADH/NAD^+^ ratio (2.6× and 4× in Fig. 4J”,K”) and found that these changes resulted from an increase in NADH levels (3× and 8× in Fig. 4J,K) and not from a decrease in NAD^+^. In addition, the loss of cI activity appeared to result in increased NAD pathway metabolites, including NAD^+^ (2× in Fig. 4K’, note this is not significant; but no change in Fig. 4J’; see Dataset EV2), possibly reflecting a compensatory increase in NAD^+^ biosynthesis. We conclude that cI inhibition resulted in NADH accumulation and increased NADH/NAD^+^ ratio. Thus, consistent with earlier results obtained in mammalian cells (Diaz-Cuadros et al, 2023), developmental speed appeared to negatively correlate with the NADH/NAD^+^ ratio in the *Drosophila* eye.

### Glycolysis maintained ATP levels constant upon loss of cI activity

Loss of cI activity should also reduce ATP production in mitochondria. To measure ATP levels, we used a ratio metric fluorescent sensor, iATPsnFR-95A.A119L (Marvin et al, 2024), noted iATPsn hereafter. This sensor is an improved version of the single-wavelength iATPsnFR sensor (Lobas et al, 2019). It carries an ATP-binding subunit of the F_0_F_1_-ATP synthase complex which was mutated to reduce its affinity to ATP down to the mM range and fused to a circularly permuted sfGFP. To perform ratio metric measurements, this ATP sensor also included the pH-sensitive pHmScarlet, noted Scarlet hereafter. This iATPsn-Scarlet biosensor was expressed ubiquitously in transgenic larvae, and analysis was performed on ex vivo cultured eye discs. Analysis of *mirr* > *ND-42*^*RNAi*^ discs showed no difference in ATP levels between *d* and *v* cells (Fig. 4L–O), suggesting that ETC inhibition had no major consequence on the steady-state level of ATP, and that reduced developmental speed did not result from low ATP levels.

We next examined the role of the ETC in ATP production. Inhibiting the activity of the ETC using Antimycin A (AntA), a cIII inhibitor (Xia et al, 1997), led to a rapid decrease in ATP levels in control *v* cells of *mirr* > *ND-42*^*RNAi*^ discs (Fig. 4L’,M). This showed that OxPhos contributes to ATP production in control UPs and DCs cells. In contrast, AntA had little effect on ATP production in *ND-42*^*RNAi*^-expressing *d* cells (Fig. 4L’,M). This showed that the activity of the ETC was already inhibited by the silencing of the *ND-42* gene and that ATP was produced by another metabolic pathway upon loss of cI activity.

Glycolysis, which breaks down glucose into pyruvate, is another important source of cellular ATP. We therefore hypothesized that glycolysis was upregulated upon ETC inhibition. To test this, we blocked glycolysis using Bromopyruvic acid (3BP), a pyruvate derivative that inhibits two key glycolytic enzymes (Ganapathy-Kanniappan et al, 2009; Li et al, 2022). Inhibition of glycolysis by 3BP led to decreased ATP levels in both control and *ND-42*^*RNAi*^ cells, with a more rapid decrease observed in the *ND-42*^*RNAi*^ cells (Fig. 4N,O). This indicated that glycolysis was active in all cells and that *ND-42*^*RNAi*^-expressing cells appeared to be more dependent on glycolysis for ATP production. We therefore conclude that increased glycolysis appeared to compensate for the loss of ETC activity, thereby maintaining ATP levels constant.

### Increased glycolysis is a key response to cI inhibition

**Figure EV2:**
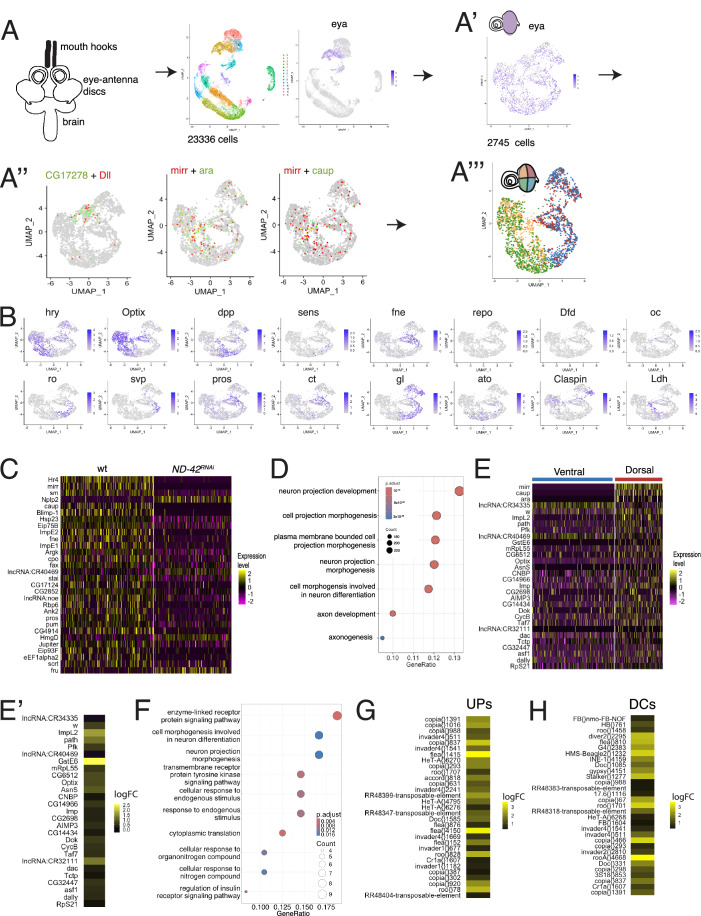
A scRNAseq analysis of the DC response to ETC inhibition. (**A**, **B**) scRNAseq was performed on cells from dissected eye-brain complexes from control Oregon-R larvae (30,045 cells) and *mirr* > *ND-42*^*RNAi*^ larvae (23,336 cells). Clustering analysis identified one cluster marked by *eya*+ cells corresponding to the eye primordium (**A**, **A’**). Cells from this cluster (2745 and 2112 cells for the *mirr* > *ND-42*^*RNAi*^ and Oregon-R samples, respectively) were further clustered (**A”**), leading to the identification of a peripodial cell cluster based on *DII* and *CG17278* gene expression (**A”**). This cluster was removed in all further analysis. The remaining UPs (1275 cells; color-coded green and orange) and DCs (1127 cells; color-coded red and blue) were identified based on marker gene expression (**A”’**, **B**). These eye cells were also categorized as *d* and *v* cells based on the expression of the *mirr, ara*, or *caup* genes (triple-negative cells were identified as *v* cells; see schematic in (**A”’**) for the color code). (**C**) Heatmap of the top 30 differentially expressed genes between wild-type *v* and *d* cells (wt, Oregon-R) and *mirr* > *ND-42*^*RNAi*^ control *v* cells (gene expression level color-coded as indicated). Genes were ranked by adjusted *P* values. A few ecdysone-regulated genes and differentiation markers appeared to be differentially expressed between these two populations of control cells. (**D**) Gene Ontology (GO) term enrichment analysis for biological pathways for the top 1781 genes. (**E**, **E’**) The top 30 differentially expressed genes between *ND-42*^*RNAi*^ *d* DCs (*n *= 414) and control *v* DCs (*n* = 713). DCs from *mirr* > *ND-42*^*RNAi*^ discs were ranked by adjusted *P* values, and expression levels were color-coded (**E**; see heatmap). Relative changes in averaged expression levels are shown as log2 Fold Change values (logFC; **E’**). The three genes used for clustering (*mirr*, *ara*, and *caup*) were removed from the analysis shown in (**E’**). (**F**) GO term enrichment analysis for biological pathways for the top 60 genes with *P* value < 0.05 in DCs. (**G**, **H**) The top 30 differentially expressed transposable elements (TE) in DCs (*n* = 73 TEs) and UPs (*n* = 53 TEs) were ranked by *P* values, and expression levels (logFC values) were color-coded as indicated. Two-sided *t* test (**D**, **F**).

**Figure 5:**
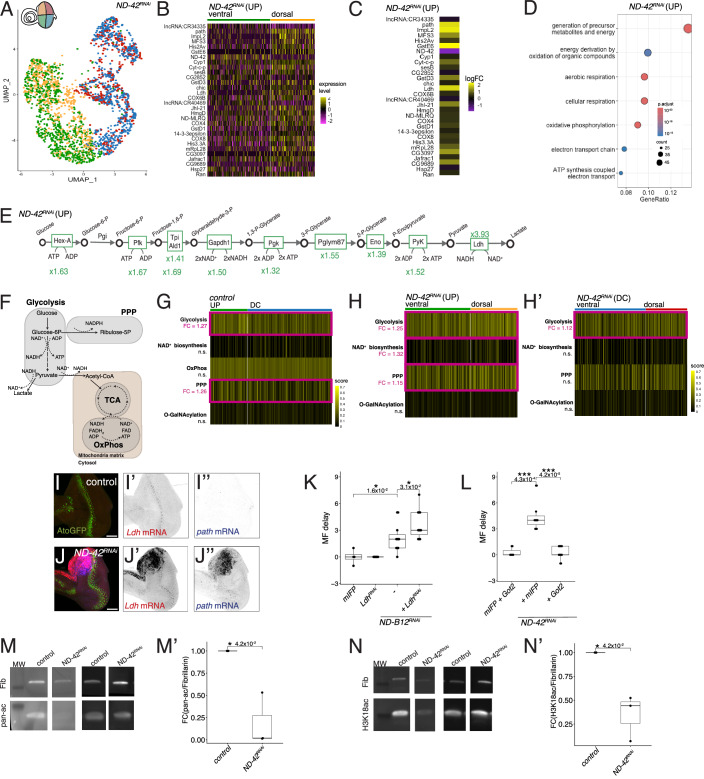
scRNAseq analysis of the compensatory response to ETC inhibition. (**A**) UMAP analysis of *mirr* > *ND-42*^*RNAi*^ eye field cells (color code as in the eye disc schematic). (**B**) List of the top 30 Differentially Expressed Genes (DEGs) between *d* (*n* = 528 cells) and control *v* UPs (*n* = 747 cells) in *mirr* > *ND-42*^*RNAi*^ eye discs. Genes were ranked by adjusted *P* value. (**C**) Relative changes in expression levels for the top 30 genes shown in (**B**). The log2 Fold Change (logFC) values were color-coded (see heatmap). (**D**) Gene Ontology (GO) term enrichment for biological pathways using the top 421 differentially expressed genes between *d* and *v* UPs in *mirr* > *ND-42*^*RNAi*^ discs (adjusted *P* value < 0.05). (**E**) Glycolysis pathway with changes in gene expression (FC, green, between *d ND-42*^*RNAi*^ and *v* control UPs). Increased glycolysis in *ND-42*^*RNAi*^ cells was in part due to changes in gene expression. (**F**–**H’**) Schematics of key energy metabolic pathways (**D**) studied in control (**E**) and *ND-42*^*RNAi*^ eye discs (**F**, **F’**) using a pathway score calculated based on our scRNAseq data (see “Methods”; for each cell, score values were shown as color-coded heatmap in (**E**–**F’**)). In control discs (**E**), Glycolysis (FC = 1.27; effect size (s)=0.59, *P* = 10^−31^) and PPP (FC = 1.26; s = 0.31, *P* = 10^−10^) were upregulated in UPs (data boxed in pink). In contrast, similar levels of OxPhos and NAD^+^ biosynthesis pathway activities were seen in UPs and DCs. In *ND-42*^*RNAi*^ eye discs (**F**, **F’**), Glycolysis was upregulated in dorsal UPs (**F**; FC = 1.2; s = 0.6, *P* = 10^−31^) and DCs (**F’**; FC = 1.1; s = 0.3, *P* = 10^−5^). PPP (FC = 1.1; s = 0.2, *P* = 10^−6^) and NAD^+^ biosynthesis (FC = 1.3; s = 0.2, *P *= 10^−5^) were increased in dorsal UPs (**F**), but not DCs (**F’**; since OxPhos activity was experimentally perturbed in *ND-42*^*RNAi*^ eye discs, its pathway activity was not measured in this context). No differences in O-GalNAcylation activity were observed within control discs nor upon silencing of the *ND-42* gene; this pathway activity served as a negative control. (**I**–**J”**) The expression of the *Ldh* (red in (**F**, **G**)) and *path* genes (blue in (**F**, **G**)) was studied by smiFISH in control *mirrGal4 ato*^*GFP*^ (**F**–**F”**) and *mirr* > *ND-42*^*RNAi*^ *ato*^*GFP*^ discs (**G**–**G”**; AtoGPP, green, marked the differentiation front). The *Ldh* and *path* genes were upregulated in UPs and dorsal peripodial cells upon silencing of the *ND-42* gene (**G’**, **G”**). Note that the *Ldh* gene was expressed by a subset of MF cells in wild-type discs (**F’**), consistent with its proposed regulation *by* Ato (Aerts et al, 2010). (**K**) The RNAi-mediated silencing of *Ldh* enhanced the MF delay resulting from the silencing of *ND-B12* (*Ldh*^*RNAi*^, 0.0 + /−0.0, *n* = 6; *ND-B12*^*RNAi*^, 2.2 + /−1.6, *n* = 11; *ND-B12*^*RNAi*^ + *Ldh*^*RNAi*^, 4.1 + /−2.4, *n* = 12). The mIFP data shown in Fig. 1E were used as a negative control in (**H**). Phenotypes were scored blind. (**L**) Overexpression of Got2 enhanced the MF delay resulting from the silencing of *ND-42* (*Got2* + *mIFP*, 0.3 + /−0.7, *n* = 12; *ND-42*^*RNAi*^ + *mIFP*, 4.2 + /−1.8, *n* = 11; *ND-42*^*RNA i*^+ *Got2*, 0.3 + /-0.5, *n* = 7; *mIFP* was used to control for the number of UAS transgenes). The data was counted blindly. (**M**–**N’**) Western blot analysis showing a decrease in histone acetylation (pan-acetylated histones, **M**, **M’**; H3K18ac, **N**, **N’**; signal intensity normalized using Fibrillarin) in *tub*^*ts*^ > *ND-42*^*RNAi*^ eye discs (*n* = 3). For each experiment, two representative western blots are shown (see Fig. EV5 for the full blots). The box plots show the median and the interquartile range from the 25th to 75th percentile; whiskers extend to data points within 1.5× the interquartile range of the lower and upper quartiles, representing the approximate minimum and maximum non-outlier values. Wilcoxon test (**K**, **L**) and two-sided *t* test (**M**–**N’**), *P* values: *<0.05; **<0.01; ***<0.0001. Scale bar, 50 µm. Source data are available online for this figure.

To further study the cellular response to the loss of ETC activity, we next performed single-cell RNA sequencing (scRNAseq). Starting from eye-brain complexes dissected from wild-type and *mirr* > *ND-42*^*RNAi*^ larvae, we sequenced ~30,000 cells per genotype and identified the eye field as a cluster of ~3000 *eya*-positive and *sim*-negative cells. Further clustering produced three sub-clusters corresponding to UPs, DCs, and peripodial cells based on key marker genes (Fig. EV2A,B). We next identified dorsal cells as cells expressing the *Iroquois Complex* genes *mirr*, *araucan* (*ara*) and/or *caupolican* (*caup*) (Fig. EV1M–N”). Triple-negative cells were identified as *v* cells (Figs. 5A and  EV2A”,A”’). Comparing control *v* cells from *mirr* > *ND-42*^*RNAi*^ larvae with control cells from *mirr-Gal4* larvae by Differential Gene Expression analysis revealed clear differences between these two populations of control cells (Fig. EV2C). Differences in Gene Ontology (GO) terms were associated with neuronal differentiation, possibly reflecting a sample-to-sample difference in staging (Fig. EV2D). We therefore used the *v* cells of the *mirr* > *ND-42*^*RNAi*^ eye discs as internal controls to study the transcriptomic response to loss of cI activity. We identified 421 and 60 differentially expressed genes (DEG) in *mirr* > *ND-42*^*RNAi*^ UPs and DCs, respectively (Figs. 5B,C and EV2E,E’; Dataset EV3; most of these genes were upregulated in *ND-42*^*RNAi*^ cells). GO analysis showed that genes involved in ATP production and OxPhos were enriched in *ND-42*^*RNAi*^ UPs (Fig. 5D). Of note, most genes encoding glycolytic enzymes were found to be upregulated in UPs (Fig. 5E). Consistent with increased Glycolysis, a glucose transporter (MFS3), a rate-limiting enzyme breaking glycogen into glucose (GlyP) and the rate-limiting enzyme contributing to the detoxification of a cytotoxic byproduct of glycolysis (GLO1) were also upregulated (Dataset EV3). In contrast, differentially expressed genes in DCs were predominantly associated with neuronal differentiation and morphogenesis (Fig. EV2E,F).

Using a previously published metric to estimate the relative activities of various metabolic pathways (Davis et al, 2024) (Fig. 5F), we first found that Glycolysis and the Pentose Phosphate Pathway (PPP), an anabolic pathway involved in the synthesis of nucleotides, appeared to be more active in the anterior proliferative UPs than in the DCs (Fig. 5G). Second, we found that Glycolysis was significantly increased in both UPs and DCs upon silencing the *ND-42* gene. We further observed that the PPP and the NAD^+^ biosynthesis pathway were significantly increased in *ND-42*^*RNAi*^ UPs (Fig. 5H,H’). Taken together, our data showed that glycolysis is a key metabolic response to cI inhibition. This response was primarily observed in UPs, possibly reflecting the metabolic constraints associated with the growth and proliferation of UPs.

**Figure EV3:**
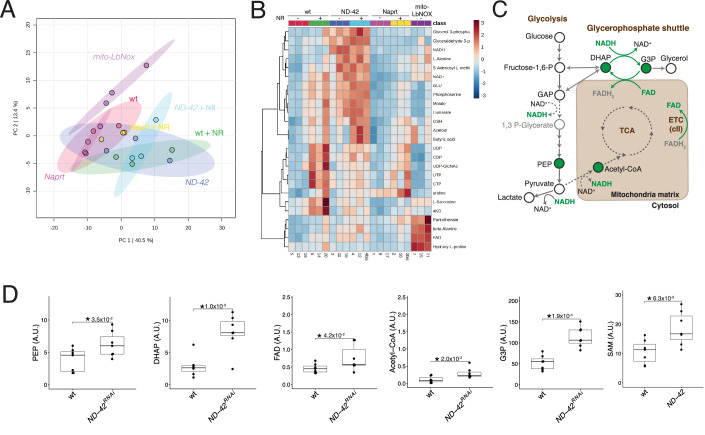
A metabolomic analysis of cI inhibition and reduced NAD^+^ recycling by LC/MS. (**A**) PCA analysis of the metabolome (149 metabolites) of whole cell extracts prepared from *tub*^*ts*^ > *+, tub*^*ts*^ > *ND-42*^*RNAi*^*, tub*^*ts*^>*Naprt*^*RNAi*^, and *tub*^*ts*^>*mito-LbNOX* eye discs. These discs were dissected from larvae grown in fly food supplemented or not with NR (as indicated). Clustering analysis indicated that the *tub*^*ts*^>*Naprt*^*RNAi*^ metabolome was distinct from those of all other samples. Also, the *tub*^*ts*^ > *ND-42*^*RNAi*^ metabolome differed from the *tub*^*ts*^ > *+* control metabolome in the absence of NR supplementation. In the presence of NR, the effect of *ND-42*^*RNAi*^ appeared to be more restricted. (**B**) Clustering analysis based on the top 25 differentially detected metabolites (abundance plotted as a heatmap). Differences in NAD, glycolysis, and TCA cycle metabolism were detected in the *tub*^*ts*^ > *ND-42*^*RNAi*^ metabolome relative to the *tub*^*ts*^ > *+* control metabolome. (**C**) Proposed changes in energy metabolism pathways based on the LC/MS results. Metabolites that showed a statistically significant increase upon *ND-42*^*RNAi*^ relative to wild-type condition appear in green (see Dataset EV2 for FC values). Enzymatic reactions proposed to be upregulated are shown as green arrows; those proposed to be down-regulated appear as dotted arrows. Limiting NAD^+^ levels are proposed to result in substrate accumulation in reactions coupled to NAD^+^ reduction, e.g., GAP. Increased DHAP and GAP levels are interpreted to suggest increased Glycerol Phosphate shuttle activity, promoting NAD^+^ regeneration in the cytosol and reduction of Flavin Adenine Dinucleotide (FAD) in mitochondria (FADH_2_ could then serve to fuel the ETC via the cII). High levels of Acetyl-CoA are proposed to accumulate due to defective TCA activity. (**D**) Plots showing the LC/MS results for the PhosphoEnolPyruvic acid (PEP), DHAP, FAD, Acetyl-CoA, G3P, and SAM (*n* = 7). The box plots show the median and the interquartile range from the 25th to 75th percentile; whiskers extend to data points within 1.5× the interquartile range of the lower and upper quartiles, representing the approximate minimum and maximum non-outlier values. Two-sided *t* test (**D**), *P* values: **<0.01.

Increased glycolysis is often accompanied by an increased rate of lactate production from pyruvate catalyzed by Ldh because this reaction regenerates NAD^+^ from NADH, and NAD^+^ is required to fuel glycolysis (Luengo et al, 2021). Consistent with increased glycolysis in UPs, we observed increased *Ldh* gene expression in UPs by scRNAseq (Fig. 5E) and smiFISH (Fig. 5I–J’). We therefore hypothesized that increased Ldh activity promotes glycolysis via the regeneration of NAD^+^. To test whether increased Ldh was part of a compensatory response to cI inhibition, we silenced the *Ldh* gene in combination with *ND-B12*, another cI subunit gene (Fig. 5K; Dataset EV1; double silencing of the *Ldh* and *ND-42* genes was not technically feasible because both RNAi constructs are at the same genomic site). While the RNAi-mediated silencing of *Ldh* had no effect on furrow progression, it strongly enhanced the weak *ND-B12*^*RNAi*^ MF delay phenotype (Fig. 5K). Thus, increased Ldh activity appeared to compensate for the loss of cI activity. We propose that Ldh acts in this context by regenerating NAD^+^ from NADH, allowing for increased glycolysis and compensatory ATP production. Metabolomic analysis of eye-antenna discs by LC/MS further supported the notion that NAD^+^ could be limiting upon cI inhibition (Fig. EV3). Indeed, the accumulation of Glyceraldehyde-3-phosphate (GAP) indicated that the NAD^+^-dependent reaction using GAP as a substrate becomes rate-limiting; the accumulation of glycerol-3-phosphate (G3P) and dihydroxyacetone phosphate (DHAP) that are produced from fructose-1,6-diphosphate (FDP) and GAP suggested that NAD^+^-producing reactions are enhanced and that the Glycerol Phosphate shuttle is activated to regenerate NAD^+^ in the cytoplasm (Fig. EV3C,D). Together, these data indicated that the rate of NAD^+^ regeneration could be limiting glycolysis upon cI inhibition.

**Figure EV4:**
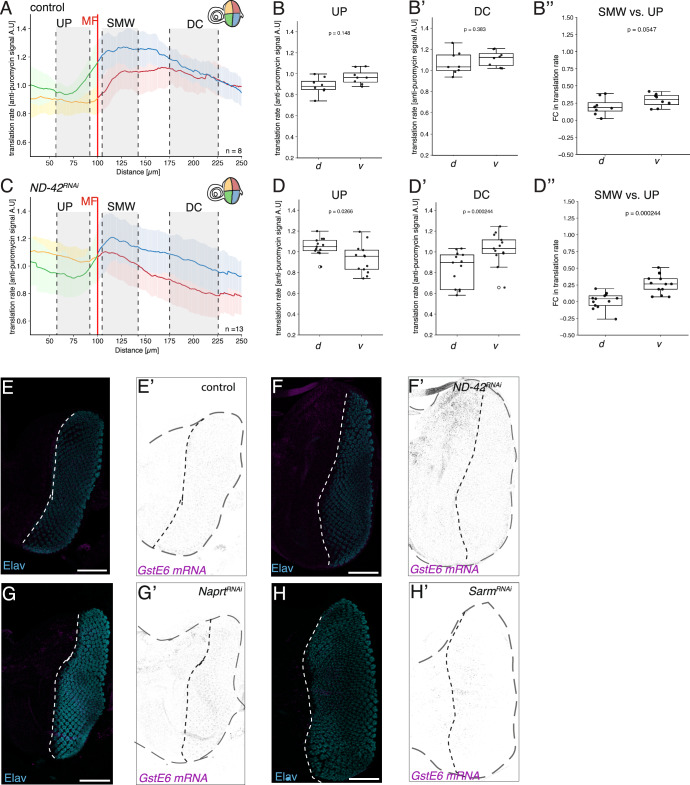
Translation rate analysis and ROS expression. (**A**–**D”**) Translation rates were inferred by measuring the intensity of the anti-puromycin signal in *mirr* > *+* (control; *n* = 8) and *mirr* > *ND-42*^*RNAi*^ (*n* = 13*)* eye discs (**A**, **C**). An increased rate of translation was detected posterior to the MF in control discs (**A**), where InR-dependent cell growth was associated with the SMW (Kim et al, 2017); this region is defined here as SMW ([105–145] in (**A**, **C**)). No statistically significant differences were observed in control discs between *d* and *v* cells in UPs (**B**; defined here as [60–90]) and DCs (**B’**; [175–225]). Likewise, a similar increase in translation from UPs to the SMW region was detected in both *d* and *v* cells (**B”**). In contrast, a mild increase in protein synthesis in UPs (**D**), but a decrease in DCs (**D’**), were noted in *d* versus *v* cells in *ND-42*^*RNAi*^ eye discs. We further found that loss of cI activity abolished the increase in protein translation in the SMW region (**D”**), consistent with reduced cell proliferation (Fig. EV1I). The *P* values from the Wilcoxon are indicated. (**E**–**H’**) The silencing of the *ND-42* gene led to increased *GstE6* expression in the dorsal UPs of *mirr* > *ND-42*^*RNAi*^ discs (**F’**; smiFISH signal in black; compare with control discs in (**E**, **E’**), and to the *v* cells in (**F’**)), indicative of increased ROS. In contrast, the silencing of the *Naprt and Sarm* genes had little effect of *GstE6* expression (**G**–**H’**), suggesting that these perturbations have a minor effect on ROS levels. Nevertheless, a clear MF delay phenotype was seen in all three RNAi conditions (Elav, cyan; the MF and the disc outline are indicated by dotted lines), suggesting that *GstE6* expression, i.e., ROS levels, did not correlate well with MF delay. The box plots show the median and the interquartile range from the 25th to 75th percentile; whiskers extend to data points within 1.5× the interquartile range of the lower and upper quartiles, representing the approximate minimum and maximum non-outlier values. Two-sided *t* test.

Beyond glycolysis, amino acid import also appeared to be upregulated upon ETC inhibition. Two transporters known to support growth upon nutrient restriction (Feng et al, 2020; Rebelo and Homem, 2023), Pathetic (*path*) and Jhl-21 were also upregulated in UPs upon silencing of *ND-42* (Fig. 5B,C,I”–J”; Dataset EV3). These gene expression changes also appeared to reflect a compensatory response since the RNAi-mediated silencing of *path* enhanced the *ND-42* MF delay phenotype (data not shown). Previous studies have shown that Aspartate is limiting for cell growth upon cI inhibition in mammals, and that the increased NADH/NAD^+^ ratio decreases the ability of cells to synthesize Aspartate, a precursor for the synthesis of Glutamate and Proline (Birsoy et al, 2015; Sullivan et al, 2015). To test whether Aspartate was also limiting in eye cells upon cI inhibition, the Glutamate oxaloacetate transaminase 2 (*Got2*) was overexpressed to favor Aspartate synthesis from OxaloAcetate. Increased *Got2* activity was found to suppress the *ND-42*^*RNAi*^ MF delay phenotype (Fig. 5L). Thus, Aspartate appeared to be limiting upon cI inhibition for the timely progression of the MF in *Drosophila*. We therefore suggest that increased amino acid import might compensate for reduced amino acid biosynthesis upon ETC inhibition. This in turn raised the possibility that changes in the NADH/NAD^+^ ratio may have an impact on protein synthesis. Using a puromycin pulse labeling approach to monitor the translation rate (Liu et al, 2012), we found that protein synthesis appeared to be upregulated in UPs and down-regulated in DCs upon *ND-42* inhibition (Fig. EV4A–D”). We also noted a relative decrease in protein synthesis at the position of the SMW upon *ND-42* inhibition. Thus, increased NADH/NAD^+^ ratio appeared to correlate with reduced translation rate in DCs but not UPs, hence reflecting differences in the compensatory responses of these two cell populations to a common metabolic perturbation.

Our analysis also identified mitochondria biogenesis and/or activity genes, and several detoxification genes (*GstD1*, *GstD3*, *GstE1*, *GstE6, GstE8, GstT1, Nmdmc*) (Sharma et al, 2004; Yu et al, 2015) were upregulated upon the silencing of *ND-42* (Dataset EV3). GstD1 is a commonly used marker for elevated Radical Oxygen Species (ROS) levels (Sykiotis and Bohmann, 2008). While GstD1 was expressed at higher levels in *ND-42*^*RNAi*^ UPs (log_2_FC = 0.62, Dataset EV3), increased up-regulation was seen for GstE6 (log_2_FC = 2.30, Dataset EV3). We therefore used GstE6 as a ROS reporter and showed that the loss of cI activity led to increased *GstE6* expression (Fig. EV4E–F’), indicative of high ROS levels as observed earlier (Owusu-Ansah et al, 2013). We also noted that a secreted antagonist of Insulin signaling (ImpL2), which mediates tissue wasting (Figueroa-Clarevega and Bilder, 2015), and the relaxin-like hormone dIlp8 were expressed at higher levels upon loss of cI activity. This suggested that a local perturbation in cellular metabolism in the eye triggered a systemic response to slow down tissue growth and developmental progression at the organismal level. This systemic response likely contributed to the developmental delay noted in *mirr* > *ND-42*^*RNAi*^ larvae (see also Fig. 3D).

**Figure EV5:**
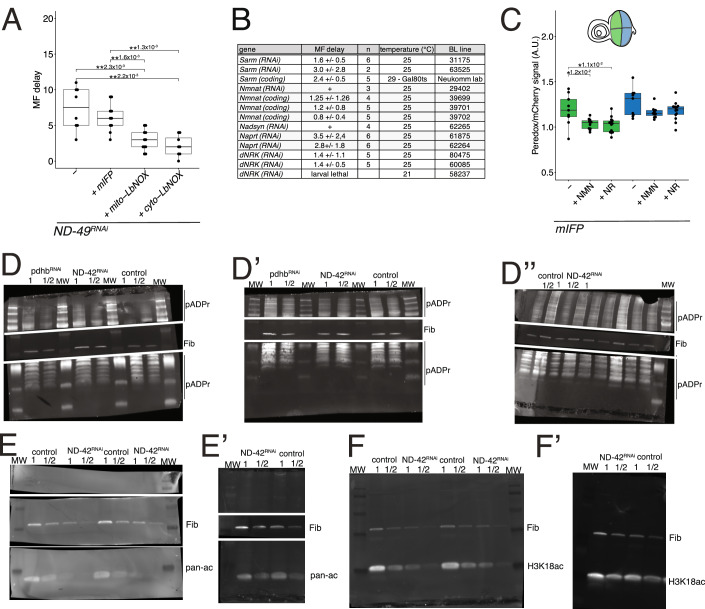
NAD^+^, cellular redox, and speed of progression of the MF. (**A**) MF delay analysis showing that the expression of cytosolic or mitochondrial LbNOX was sufficient to reduce the delay resulting from the silencing of the *ND-49* gene: *mirr* > *ND-49*^*RNAi*^: 7.0 + /−3.4 (*n* = 5); *mirr* > *ND-49*^*RNAi*^ + *mIFP*: 6.1 + /−2.0 (*n* = 10); *mirr* > *ND-49*^*RNAi*^ *+ mito-LbNOX*: 2.9 + /−1.4 (*n* = 11); *mirr* > *ND-49*^*RNAi*^ *+ cyto-LbNOX*: 2.1 + /−1.5 (*n* = 8). (**B**) MF delay analysis of NAD metabolism gene (see Fig. 7C). ‘+’ indicated that the number of ommatidial rows were not counted as discs looked clearly similar to wt. (**C**) Analysis of the Peredox normalized intensity ratio in *mirr* > *mIFP* (*n* = 9) eye discs indicated that addition of NMN (*n* = 8) and NR (*n* = 11) lowered the cellular redox in UPs, but not DCs. (**D**–**D”**) Western blot analysis of PARylation in control and *ND-42*^*RNAi*^ eye discs. Fibrillarin (Fib) was used to normalize the poly-ADPr signal (measured as the sum of the signals from top and bottom parts of the blots). Each sample was loaded twice (with a 2-fold difference in volume; MW, Molecular Weight; same blot as in Fig. 7H). (**E**–**F’**) Western Blot analysis of acetylation in control and *ND-42*^*RNAi*^ eye discs. Fibrillarin (Fib) was used to normalize the pan-acetylation (pan-ac; **E**–**E’**) and H3K18ac signals (**F**–**F’**). Each sample was loaded twice (with a twofold difference in volume; MW, Molecular Weight; same blot as in Fig. 5M–N’). The box plots show the median and the interquartile range from the 25th to 75th percentile; whiskers extend to data points within 1.5× the interquartile range of the lower and upper quartiles, representing the approximate minimum and maximum non-outlier values. Wilcoxon test (**A**, **C**), *P* values: *<0.05; **<0.01.

Finally, we noted that several transposable elements (TEs) were expressed at a higher level in UPs and DCs upon cI inhibition (Fig. EV2G,H; Dataset EV4). Since increased glycolysis has broad impact on histone modifications (Dai et al, 2020) and since TEs are usually silenced epigenetically, we speculate that these changes in TE expression might reflect a global impact of the cellular metabolism on the epigenome. Consistent with a previous study showing that increasing S-Adenosyl Methionine (SAM) levels led to the transcriptional activation of TEs in *Drosophila* (Hayashi et al, 2025), the deregulation of TEs in *ND-42*^*RNAi*^ cells correlated with high levels of SAM (Fig. EV3D). Increased glycolysis was also associated with high levels of Acetyl-CoA (Figs. 5M–N’ and  EV3D). However, and paradoxically, lower levels of Histone acetylation, including H3K18ac, were detected in *ND-42*^*RNAi*^ eye-antenna discs (Figs. 5M–N’ and EV5E–F’). These observations might suggest that cI inhibition primarily led to Acetyl-CoA accumulation in mitochondria (see “Discussion”).

In summary, our analysis showed that *ND-42*^*RNAi*^ cells elicited both local and systemic responses to loss of ETC activity, and that increased glycolysis was a key compensatory response elicited by the proliferative undifferentiated cells, which likely resulted in a strong demand for NAD^+^(Luengo et al, 2021).

### The speed of the MF depends on the metabolic state of the undifferentiated cells

**Figure 6:**
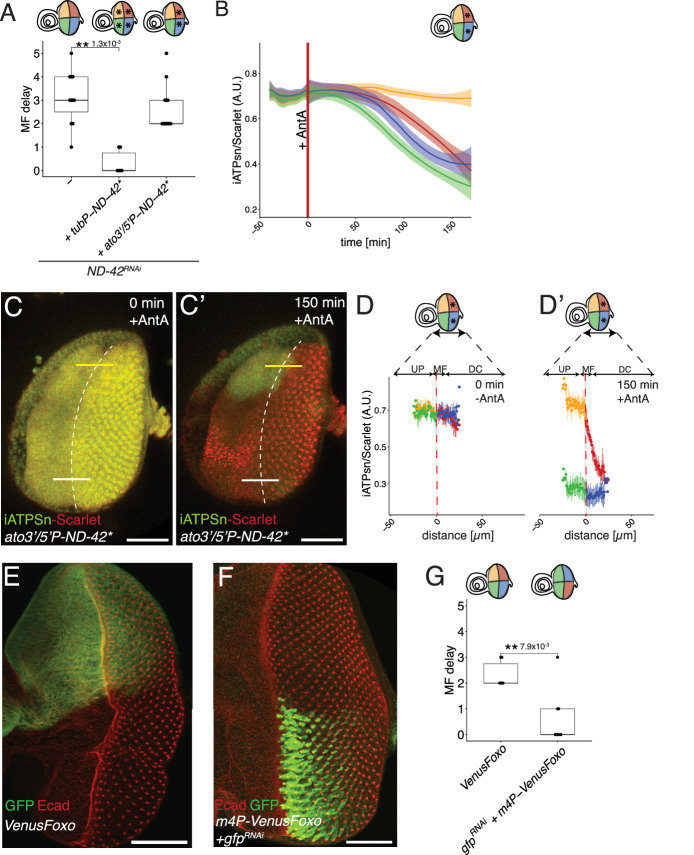
A UP-specific metabolic perturbation slowed down the progression of the front. (**A**) A UP-specific perturbation in energy metabolism was produced in *mirr* > *ND-42*^*RNAi*^ discs by expressing a RNAi-resistant form of ND-42, ND-42*, in DCs only (as indicated with * in DC quadrants). As a positive control, ND-42* rescued the *ND-42*^*RNAi*^ MF delay phenotype when expressed ubiquitously (as indicated with * in all four quadrants of the eye). This UP-specific perturbation resulted in a strong MF delay: *mirr* > *ND-42*^*RNAi*^, 3.9 + /−1.1 (*n* = 11); *mirr* > *ND-42*^*RNAi*^ tubP-ND-42*, 0.3 + /−0.5 (*n* = 6); *mirr* > *ND-42*^*RNAi*^ ato3’/5’P-ND-42*, 2.6 + /−0.9 (*n* = 12). (**B**–**D’**) UP-specific perturbation in *mirr* > *ND-42*^*RNAi*^ ato3’/5’P-ND-42* eye discs. Cells with increased glycolysis were identified using the iATPSn sensor based on their response to AntA. Time course analysis of the normalized iATPSn signal showed that the lack of response to AntA, indicative of increased glycolysis, was restricted to dorsal UPs (**B**; *n* = 4; the data are shown as in Fig. 4M). The iATPSn signal (green; Scarlet, red) is shown on snapshot views of a disc at *t *= 0 (**C**) and t = 150 (**C’**). This indicated that the loss of endogenous *ND-42* activity was efficiently compensated by ectopic ND-42*. The normalized iATPSn signal (mean values, shown as dots, and standard error of the mean) was plotted along the AP axis (yellow line on *d* side and white line for *v* side in (**C**, **C’**); MF, dashed line) at *t* = 0 (D) and *t* = 150 (**D’**; *n* = 4). This showed that the suppression of the *ND-42*^*RNAi*^ phenotype was first detected at the MF. (**E**–**G**) Expression of VenusFoxo in *mirr>VenusFoxo* discs (**E**) led to a MF delay phenotype (**G**; 2.3 + /0.5, *n* = 6). In contrast, restricting the expression of VenusFoxo to ventral DCs in *m4P-VenusFoxo ey-FLP mirr>gfp*^*RNAi*^ discs (**F**; ey-FLP was used to excise the FRT stop cassette of the VenusFoxo transgene) had no effect on MF progression (0.6 + /−1.0, *n* = 9). This indicated that restricting metabolic perturbations to DCs did not slow the progression of the MF. The box plots show the median and the interquartile range from the 25th to 75th percentile; whiskers extend to data points within 1.5× the interquartile range of the lower and upper quartiles, representing the approximate minimum and maximum non-outlier values. Wilcoxon test, *P* values: **<0.01. Scale bars, 50 µm. Source data are available online for this figure.

Our data above showed that metabolic rewiring was particularly noticeable in UPs. This compensatory response to cI inhibition was consistent with the observation that *ND-42*^*RNAi*^ UPs were still actively growing and proliferating despite cI inhibition, however, at a lower rate (Fig. EV1I). This observation suggested that the speed of progression of the MF was dependent on the metabolic state of these cells. To test this, we sought to restrict the RNAi-mediated silencing of *ND-42* to UPs using a RNAi-resistant form of ND-42 expressed specifically in cells posterior to the MF. We therefore designed a RNAi-resistant *ND-42* gene, noted ND-42*, by recoding the region of its Open Reading Frame targeted by the RNAi construct (see Methods). The expression of ND-42* under the control of a constitutive promoter was sufficient to restore the progression of the MF in *mirr* > *ND-42*^*RNAi*^ larvae (Fig. 6A), hence validating this approach. We then used strong enhancers from the *atonal* (*ato*) gene to express ND-42* in MF cells and examined whether these ato-ND-42* transgenes could restore OxPhos activity in the *mirr* > *ND-42*^*RNAi*^ cells posterior to the MF. Using the iATPsn sensor to examine changes in ATP levels upon inhibition of the ETC by AntA, we found that expression of ND-42* in the MF restored production of ATP by the ETC in *mirr* > *ND-42*^*RNAi*^ DCs (Fig. 6B). We conclude that the ND-42* protein restored OxPhos activity upon silencing of the endogenous *ND-42* gene and that the ND-42* protein was stable enough to rescue cI activity in all DCs. Analysis of the iATPsn fluorescence signal along the AP axis showed that expression of ND-42* in the MF rapidly restored OxPhos activity in DCs (Fig. 6C–D’). Thus, RNAi-mediated silencing appeared to be restricted to cells located anterior to, and at the MFs in these discs. We next examined the MF delay in these discs and found that restoring ND-42 activity in cells posterior to the MF did not suppress the delay in MF progression (Fig. 6A). This indicated that the activity of ND-42 was required in UPs and/or MF cells for the timely progression of the MF. Finally, to test whether the metabolic state of the DCs may also be required to set the speed of the MF, we sought to specifically perturb the metabolic state of the DCs. To do so, we restricted the expression of a Venus-tagged version of Foxo, which was found to delay the progression of the MF when expressed in all *d* cells (Fig. 6E,G), in DCs using a strong enhancer from the *E(spl)m4-BFM* gene which is active in the MF (Couturier et al, 2026), and then used *mirr>gfp*^*RNAi*^ to silence VenusFoxo expression in *d* cells, hence restrict the expression of VenusFoxo to *v* cells. In this context, no MF delay was observed in *v* cells (Fig. 6F,G), suggesting that the metabolic state of the DCs does not play a critical role in the progression speed of the MF. We therefore conclude that the metabolic states of the UPs and/or MF cells determine the speed of progression of the MF.

### Cellular NAD^+^ availability appeared to constrain developmental speed

**Figure 7:**
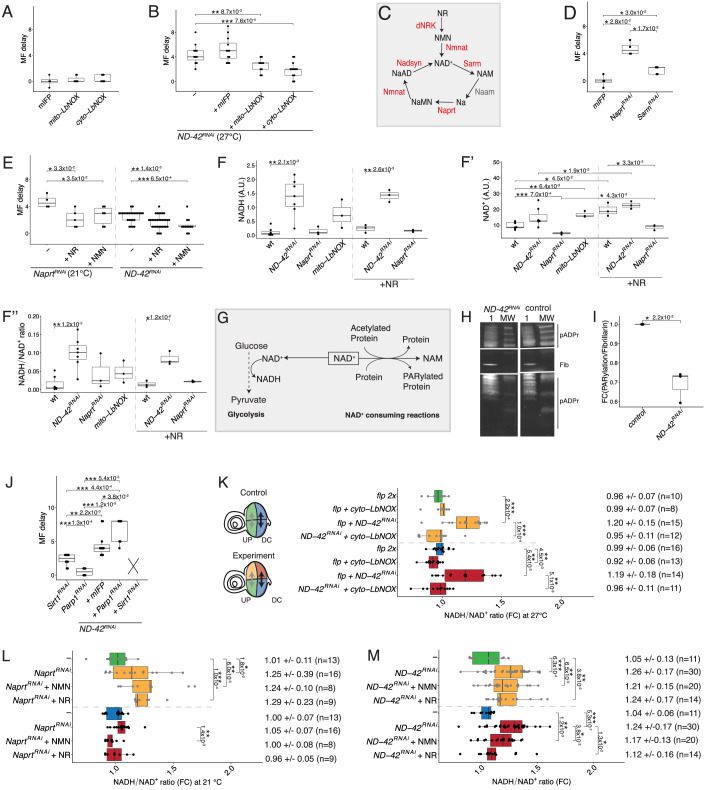
The rate of NAD^+^ regeneration appeared to limit developmental speed. (**A**, **B**) MF delay analysis showing that expression of cyto-LbNOX and mito-LbNOX had no significant effect on MF progression (**A**; control mIFP data are from Fig. 1E) but efficiently suppressed the *ND-42*^*RNAi*^ MF delay phenotype (**B**). The strong MF delay seen in *mirr* > *ND-42*^*RNAi*^ at 27 °C (4.4 + /−1.6, *n* = 11; negative control with mIFP co-expression: 5.3 + /−2.0, *n* = 12) was suppressed by mito-LbNOX (2.6 + /−1.1, *n* = 10) and cyto-LbNOX (1.9 + /−1.3, *n* = 13). Phenotypes were scored blind. (**C**) A simplified view of NAD^+^ metabolic pathway: NAM nicotinamide, Na nicotinic acid, NaMN nicotinic acid mononucleotide, NaAD nicotinic acid adenine dinucleotide. The genes tested for possible MF delay by RNAi-mediated silencing and/or overexpression are indicated in red (**C**). (**D**) Silencing the *Sarm* and *Naprt* genes delayed the progression of the MF (*Sarm*^*RNAi*^*:* 1.6 + /0.5, *n* = 5; *Naprt*^*RNAi*^: 4.8 + /−1.0, *n* = 4). (**E**) The *Naprt*^*RNAi*^ phenotype was suppressed in part by the addition of NR (2.2 + /−1.3, *n* = 5) and NMN (2.6 + /−1.4, *n* = 6). Likewise, the *ND-42*^*RNAi*^ defect (2.6 + /−0.9, *n* = 25) was partially suppressed by adding NR (1.8 + /−1.0, *n* = 33) or NMN (1.25 + /−1.1, *n* = 10). Phenotypes were scored blind. (**F**–**F”**) LC/MS analysis of the NADH (**F**) and NAD^+^ levels (**F’**) and of the NADH/NAD^+^ ratio (**F”**). While addition of NR had no statistically significant effect on NADH levels in control *tub*^*ts*^ > *+* (*n* = 7; +NR, *n* = 3), *tub*^*ts*^ > *ND-42*^*RNAi*^ (*n* = 7; +NR, *n* = 3), and *tub*^*ts*^>*Naprt*^*RNAi*^ eye discs (*n* = 3; +NR, *n* = 3), NR addition led to increased NAD^+^ levels for all three genotypes (**F**, **F’**). A similar increase was also observed upon NR addition in *tub*^*ts*^>*mito-LbNOX* discs (**F**; *n* = 3). As expected, *Naprt*^*RNAi*^ led to a strong reduction in NAD^+^ levels (**F’**). Despite these, no statistically significant changes in NADH/NAD^+^ ratio were observed upon NR addition (**F”**). Pairwise statistical analysis was performed between control and each experimental genotype separately for each food condition, as well as between food conditions for each genotype. (**G**) NAD^+^ has two functions, as a redox cofactor in energy metabolism, e.g., glycolysis, and as a co-substrate in NAD^+^-consuming reactions. For instance, Parp1 and Sirtuins use NAD^+^ to poly-PARylate and deacetylate proteins, respectively. (**H**, **I**) Western blot analysis showing a decrease in PARylation in *tub*^*ts*^ > *ND-42*^*RNAi*^ eye discs relative to control *tub*^*ts*^ > *+* discs (**H**, **I**; full Western Blots shown in Fig. EV5). The PARylation levels normalized to Fibrillarin (*n* = 3). (**J**) The RNAi-mediated silencing of *Parp1* enhanced the MF delay resulting from the silencing of *ND- 42* (*Parp1*^*RNAi*^, 0.5 + /−0.5, *n* = 6; *ND-42*^*RNAi*^ + *mIFP*, 4.2 + /−1.8, *n* = 11; *ND-42*^*RNAi*^+*Parp*^*RNAi*^, 6.8 + /−1.8, *n* = 7). The RNAi-mediated silencing of *Sirt1* slowed down the speed of the MF (*Sirt1*^*RNAi*^, 2.3 + /−0.8, *n* = 10). The double silencing of *ND-42* and *Sirt1* was larval lethal (as indicated by a cross). The *ND-42*^*RNAi*^ + *mIFP* data shown here are from Fig. 5L. (**K**) Analysis of the effect of cyto-LbNox on the NADH/NAD^+^ ratio (plots as in Fig. 4I). The expression of cyto-LbNOX suppressed the effect of *ND-42*^*RNAi*^ in both UPs (green and yellow) and DCs (blue and red; see eye schematics). In all experiments, *d* cells were identified using the infrared signal produced by mIFP (one copy of the UAS-mIFP transgene was present in all genotypes). All genotypes include the same number of UAS-X transgenes (this was achieved using control *UAS-flippase (flp)* transgenes (*flp2x*, *n* = 16; *flp + cyto-LbNOX*), *n* = 13; *flp* + *ND-42*^*RNAi*^, *n* = 11; *ND-42*^*RNAi*^ *+ cyto-LbNOX*, *n* = 14). (**L**) Analysis of the effect of *Naprt*^*RNAi*^ on the NADH/NAD^+^ ratio measured as in (**G**) (this experiment was performed at 21 °C to improve the viability of *Naprt*^*RNAi*^ larvae). Inhibition of the NAD^+^ recycling pathway led to elevated NADH/NAD^+^ ratio in UPs but not DCs. Adding NR and NMN did not change the NADH/NAD^+^ ratio measured in UPs (*Naprt*^*RNAi*^, *n* = 16; *Naprt*^*RNAi*^ + NR, *n* = 11, *Naprt*^*RNAi*^ + NMN, *n* = 10, control, *n* = 13). (**M**) Analysis of the effect of NAD^+^ precursors on the NADH/NAD^+^ ratio measured in *mirr* > *ND-42*^*RNAi*^ discs using Peredox (as in (**F**)). The addition of NR and NMN had no effect on the NADH/NAD^+^ ratio in UPs and reduced this ratio only in DCs (*ND-42*^*RNAi*^ + NR, *n* = 14; *ND-42*^*RNAi*^ + NMN, *n* = 20). Note that the control (*mirr* > *mIFP*) and *ND-42*^*RNAi*^ data were from Fig. 4I. The box plots show the median and the interquartile range from the 25th to 75th percentile; whiskers extend to data points within 1.5× the interquartile range of the lower and upper quartiles, representing the approximate minimum and maximum non-outlier values. Wilcoxon test (**A**–**E**, **H**–**K**) two-sided *t* test (**F**–**F’**, **L**, **M**), *P* values: *<0.05; **<0.01; ***<0.0001. Source data are available online for this figure.

We next examined whether the rate of NAD^+^ regeneration from NADH could be limiting the speed of progression of the MF upon cI inhibition. To test this, we overexpressed a bacterial NADH oxidase, LbNOX, to increase the rate of NAD^+^ regeneration (Titov et al, 2016). Expression of LbNOX in the cytosol, using cyto-LbNOX, had no detectable effect on MF progression (Fig. 7A) and only slightly decreased the NADH/NAD^+^ ratio in differentiated eye disc cells (Fig. 7K; the weak effect measured in UPs was not statistically significant). Likewise, expression of LbNOX in mitochondria, using mito-LbNOX, did not delay the MF and had no significant effect on the NADH/NAD^+^ ratio as measured by LC/MS in eye-antenna disc extracts (Fig. 7F”). However, the levels of both NAD^+^ and NADH appeared to be increased upon mito-LbNOX expression (Fig. 7F,F’), suggesting that increasing the pools of NAD^+^ might compensate for a loss of reduced NADH in mitochondria.

We next combined cyto-LbNOX and mito-LbNOX with *ND-42*^*RNAi*^ and found that both cyto-LbNOX and mito-LbNOX were sufficient to partially suppress the MF delay seen in *mirr* > *ND-42*^*RNAi*^ discs (Fig. 7B). Expression of cyto-LbNOX and mito-LbNOX had similar effects in *mirr* > *ND-49*^*RNAi*^ discs (Fig. EV5A; *ND-49* encodes another cI subunit gene; see Dataset EV1). This showed that increasing the rate of NAD^+^ regeneration partially restored developmental speed in cI-inhibited eye discs. Peredox analysis further showed that expression of cyto-LbNOX fully suppressed the effect of *ND-42*^*RNAi*^ on the NADH/NAD^+^ ratio (Fig. 7K). Thus, the rate of NAD^+^ regeneration from NADH appeared to be limiting upon cI inhibition for the timely progression of the MF.

To further examine the role of NAD^+^ in the progression speed of the MF, we altered the level of cellular NAD^+^ by adding precursors of NAD^+^ biosynthesis in the fly food and/or by silencing genes of the NAD^+^ salvage pathway (Gossmann et al, 2012) (Fig. 7C). Testing five of the six enzymes involved in the NAD^+^ salvage pathway, we found that silencing the *Sterile alpha and Armadillo motif* (*Sarm*) and *Nicotinate phosphoribosyltransferase* (*Naprt*) genes led to a MF delay phenotype (Figs. 7D and EV5B). This MF delay did not appear to be associated with increased ROS levels, monitored here using *GstE6* as a reporter (Fig. EV4G–H’), suggesting that increased ROS did not contribute to the MF delay in these contexts. Adding the NAD^+^ precursors Nicotinamide Riboside (NR) and Nicotinamide MonoNucleotide (NMN) to the fly food increased the level of NAD^+^ in *Naprt*^*RNAi*^ eye-antenna disc cells (Fig. 7F’) and suppressed the *Naprt*^*RNAi*^ MF delay phenotype (Fig. 7E). This suggested that limiting NAD^+^ availability resulted in a MF delay in *mirr>Naprt*^*RNAi*^ discs. To further test whether NAD^+^ could also limit developmental speed in *ND-42*^*RNAi*^ cells, we added NR or NMN to the food of *mirr* > *ND-42*^*RNAi*^ larvae. Adding these NAD^+^ precursors led to a partial suppression of the *ND-42*^*RNAi*^ MF delay phenotype (Fig. 7E). This suggested that NAD^+^ constrained the speed of progression of the MF upon cI inhibition.

Next, we examined the activity of a non-redox NAD^+^-dependent enzyme. Indeed, beyond its role as a co-factor in many redox reactions, including glycolysis, NAD^+^ is also an essential co-substrate for non-redox NAD^+^-dependent enzymes, including Sirtuins and poly(ADP-ribose) polymerases (PARPs; Figs. 7G and EV5D). Protein poly-PARylation by Parp1 consumes NAD^+^. Therefore, lowering the level of free NAD^+^ might lead to decreased protein PARylation. We measured the levels of poly-PARylated proteins in whole disc extract of *tub*^*ts*^ > *ND-42*^*RNAi*^ eye-antenna discs by western blot analysis and observed a strong decrease in protein poly-PARylation upon cI inhibition (Figs. 7H,I and EV5D–D”’). This result was consistent with NAD⁺ being limiting upon cI inhibition. To test whether PARylation could contribute to regulate MF progression, we examined *mirr>Parp1*^*RNAi*^ discs. While the silencing of *Parp1*^*RNAi*^ did not produce a MF delay, the silencing of *Parp1 in ND-42*^*RNAi*^ discs enhanced the *ND-42*^*RNAi*^ MF delay phenotype (Fig.7J). This indicated that the activity of Parp1 becomes limiting in this perturbation context. We next examined the role of Sirtuin1 (Sirt1), a NAD^+^-dependent deacetylase. The silencing of *Sirt1* in *mirr>Sirt1*^*RNAi*^ induced a MF delay (Fig. 7J). It also led to lethality in combination with *ND-42*^*RNAi*^ (Fig. 7J). While the functions of Parp1 and Sirt1 in developmental speed to be further studied, these results supported the view that limiting NAD^+^ contributes to constrain developmental speed.

Studies in mouse cultured cells showed that supplementation of NAD^+^ precursors not only increased the pool of NAD+ but also changed the NADH/NAD+ ratio (Figley et al, 2021; Hu et al, 2021). Likewise, we found that adding NR not only increased the level of NAD^+^ in control eye-antenna discs (Fig. 7F,F’) but also reduced the NADH/NAD^+^ ratio in control UPs (Fig. EV5C; a similar effect was seen with NMN addition; however, no significant change in NADH/NAD^+^ was detected in whole discs by LC/MS, Fig. 7F”). Thus, NADH/NAD^+^ ratio values and cellular NAD^+^ levels appeared to be interdependent. Conversely, downregulating NAD^+^ levels through the inhibition of the salvage pathway in *mirr>Naprt*^*RNAi*^ discs was also associated with an increase in the NADH/NAD^+^ ratio in UPs (Fig. 7L). Despite this, we observed that the suppression of the *Naprt*^*RNAi*^ MF delay by the addition of NR and NMN was not accompanied by detectable changes in the NADH/NAD^+^ ratio in UPs (Fig. 7L). Likewise, the suppression of the *ND-42*^*RNAi*^ MF delay phenotype by NR and NMN was not associated with changes in the NADH/NAD^+^ ratio in UPs (Fig. 7M). In contrast, adding NR and NMN reduced the NADH/NAD^+^ ratio in *ND-42*^*RNAi*^ DCs (Fig. 7M). This suggested that the NR/NMN treatment provides enough NAD^+^ to respond to the moderate demand for NAD^+^ in DCs, but not in UPs, possibly because the energy demand in *ND-42*^*RNAi*^ UPs was higher, hence leading to a persistently high NADH/NAD^+^ ratio. Since a change in the progression of the MF was not associated with a detectable change in cellular redox in UPs, this suggested that limiting NAD^+^ appeared to constrain the speed of progression of the MF in the developing eye disc of *Drosophila*.

## Discussion

The eye imaginal discs of *Drosophila* offer two key advantages to study in vivo the speed of developmental patterning. First, the crystal-like pattern of ommatidia that forms behind the differentiation front permits to easily map time onto space. Second, gene-specific perturbations can be restricted to the dorsal compartment of the developing eye disc, allowing us to study relative changes in developmental speed at the tissue level independently of any associated changes taking place at the organismal level. Here, we made use of these two advantages to develop a simple assay that identified gene-based perturbations leading to a relative change in the progression speed of the differentiation front. The importance of these advantages can be illustrated with the *ND-42*^*RNAi*^ perturbation that induced a developmental delay associated with the up-regulation of dilp8, which is known to reduce the production of the steroid hormone ecdysone, yet produced an easy readout in the MF delay assay. A well-known limitation of RNAi is that this approach often results in partial loss of function (Dietzl et al, 2007). This limitation turned out to be useful for uncovering the role of key metabolic proteins that have essential functions. Moreover, silencing efficiency could be further modulated using the Gal4/Gal80ts system. For instance, while a delay in MF progression was detectable upon conditional silencing of the translation initiation factor eIF2α, a stronger downregulation led to defective eye growth. Finally, the issue of specificity, i.e., off-target, could be addressed using more than one RNAi line per gene, or by targeting different subunits of protein complexes.

Loss-of-function perturbations directly affecting mitochondrial metabolism were found to slow down the progression of the furrow. Thus, our work extends to insects the recent conclusion that ETC activity and mitochondrial activity regulate the rate of development in mammals (Diaz-Cuadros et al, 2023; Iwata et al, 2023). Our study showed that ETC dysfunction in *ND-42*^*RNAi*^ cells led to a loss of mitochondrial ATP production and that this loss was compensated by an increase in glycolysis. While ATP levels remained largely unchanged in *ND-42*^*RNAi*^ cells, it is possible that the rates of ATP synthesis and consumption were altered upon cI perturbation. Indeed, increased ATP consumption was detected upon cI inhibition in cultured human cells (Sercel et al, 2024; Sturm et al, 2023). Whether cI inhibition affects the flux of ATP remains to be tested. Gene expression studies indicated that glycolysis and amino acid import were predominantly upregulated in UPs anterior to the MF, possibly reflecting a higher demand for energy associated with growth and proliferation. Indeed, the costs associated with growth and division, including the cost of protein synthesis (Buttgereit and Brand, 1995), is thought to be one order of magnitude higher than the costs associated with maintenance (Lynch and Marinov, 2015). Also consistent with a strong demand for energy in UPs upon cI inhibition, a strong antioxidant response and an increased rate of protein synthesis were observed in these proliferative cells. In contrast, differentiated cells showed a reduced rate of protein synthesis and a weaker antioxidant response. Thus, the minor difference in energy metabolism, i.e., Glycolysis and PPP, which was seen between UPs and DCs in wild-type discs, appeared to be amplified upon loss of *ND-42* activity in the eye. We further speculate that the increased rate of protein synthesis seen in *ND-42*^*RNAi*^ UPs might result from proteostasis defects associated with increased oxidative stress. In contrast, cells located posterior to the MF showed a reduced rate of protein synthesis, accompanied by a less dramatic shift towards glycolysis and antioxidant response, suggesting that these *ND-42*^*RNAi*^ DCs primarily responded to the loss of ETC by spending less energy to produce proteins. This would be consistent with the idea that inhibition of the ETC activity drives metabolism away from its optimum, leading to inefficient energy consumption, with more energy being spent on maintaining structures (Sturm et al, 2023) and less energy for energetically costly processes, e.g., the synthesis of proteins with rapid turnover.

The loss of ETC activity in eye cells also led to the accumulation of NADH. Increased NADH levels relative to NAD^+^ were seen not only in mitochondria but also in the cytosol and nucleus. High NADH levels were proposed to have detrimental effects on the cellular metabolism and previous studies had shown that these can be alleviated by enhancing the regeneration of NAD^+^ via enhanced production of lactate by Ldh (King and Attardi, 1989) or via the expression of the bacterial NADH oxidase LbNOX (Titov et al, 2016). Consistent with this, expression of LbNox in mitochondria or in the cytosol suppressed in part the effect of *ND-42*^*RNAi*^, and Ldh was overexpressed in the *ND-42*^*RNAi*^ eye UP cells that grow and proliferate. Moreover, while Ldh is expressed at low levels and its activity is largely dispensable in control eye cells, the activity of Ldh became critical upon cI inhibition. Thus, excessive NADH, limiting NAD^+^, and/or elevated NADH/NAD^+^ appeared to have detrimental effects on cellular metabolism and were associated with slow developmental speed in the *Drosophila* eye.

A recent study showed that the NADH/NAD^+^ ratio plays a causal role in the regulation of developmental speed in mammals, with slower developmental speed in human cells than in mouse cells caused by a higher NADH/NAD^+^ ratio (Diaz-Cuadros et al, 2023). Most of our data are consistent with this view. Notably, an elevated cellular NADH/NAD^+^ ratio correlated with a slow progression of the MF upon *ND-42* or *Naprt* knockdown. However, we also noted that an increased NADH/NAD^+^ ratio positively correlated with a high demand for NAD^+^. For instance, limiting NAD^+^ availability was associated with an increased NADH/NAD^+^ ratio upon inhibition of the NAD^+^ recycling pathway. Conversely, increasing NAD^+^ availability by NR addition reduced the NADH/NAD^+^ ratio in growing UP cells of control larvae. Likewise, decreasing the NADH/NAD^+^ ratio by expressing mito-LbNOX led to a compensatory increase in the level of NAD^+^ (this compensatory response could help maintain a normal redox state). Also, increased NADH/NAD^+^ in *ND-42*^*RNAi*^ cells appeared to be associated with limiting NAD^+^, as suggested by (i) the suppression of the *ND-42*^*RNAi*^ MF delay phenotype by NR; (ii) the decreased protein PARylation measured in *ND-42*^*RNAi*^ cells; (iii) our transcriptomic and metabolomic results indicating that reactions regenerating NAD^+^ from NADH were upregulated (see for instance the increased expression of the *Ldh* gene), whereas reactions producing NADH from NAD^+^ were inhibited (as suggested for instance by the increased accumulation of GAP in the glycolysis pathway). These observations strongly suggested that a strong inter-dependency exists between changes in cellular redox and NAD^+^ availability. This in turn implied that disentangling the effects due to changes in NADH/NAD^+^ ratio from those due to reduced NAD^+^ availability may prove to be a complex task. Despite this, two observations suggested that limiting NAD^+^ availability had an impact beyond its effect on cellular redox on the speed of progression of the MF in the fly eye. First, the addition of NAD^+^ precursors restored in part the speed of progression of the MF in *Naprt*^*RNAi*^ cells without changing the NADH/NAD^+^ ratio measured in UP cells. Second, the addition of NAD^+^ precursors partly suppressed the *ND-42*^*RNAi*^ MF delay phenotype, indicative of restored developmental speed, whereas the cellular redox of UPs remained largely unchanged. Since the metabolic state of UPs determined the speed of MF progression, this suggested that a change in developmental speed may not necessarily correlate with a change in cellular redox. We therefore suggest that the availability of NAD^+^ contributes to limit the speed of development in the *Drosophila* eye. NAD^+^ is known to couple energy metabolism with gene expression and cellular organization, and maintaining proper NAD^+^ levels is key in various physiological contexts in mammals (Abdellatif et al, 2022; Beltrà et al, 2023; Brenner, 2022; Hosios and Vander Heiden, 2018; Luengo et al, 2021; Romani et al, 2021; Zhang et al, 2024). In *Drosophila* NAD^+^ levels vary with temperature, with lower levels observed at low and high temperature, and higher levels at optimal temperature for adult physiology and lifespan, therefore suggesting that low NAD^+^ may reflect a departure from optimal cellular homeostasis (Klepsatel et al, 2020). Interestingly, NAD^+^ is not only a key redox cofactor for energy metabolism but is also a substrate for protein post-translational modifications enzymes. Modifications include ADP-ribosylation and acetylation, and protein targets includes glycolytic enzymes, histones, and microtubules (Kulkarni and Brookes, 2019). Interestingly, our analysis provided evidence for reduced protein ADP-ribosylation upon cI inhibition, and loss of *Parp1* activity enhanced the *ND-42*^*RNAi*^ MF delay phenotype. These observations are consistent with a model whereby reduced protein PARylation, possibly due to low ADPr, contributes to constrain developmental speed in the developing eye. In addition, we observed reduced levels of Histone acetylation, including H3K18ac, in *ND-42*^*RNAi*^ eye discs. This result appeared to be paradoxical since inhibition of the cI led to increased levels of total acetyl-CoA. However, our results did not distinguish between nuclear/cytosolic acetyl-CoA and mitochondrial acetyl-CoA, and it is thus unclear whether the levels of nuclear acetyl-CoA were elevated upon cI inhibition. It will thus be interesting to examine whether acetyl-CoA specifically accumulated in mitochondria because of defective Oxphos, and to study whether and how reduced levels of Histone acetylation might contribute to slow down the speed of progression of the MF. Consistent with this hypothesis, a recent study proposed that Histone acetylation positively correlates with the rate of temporal patterning in the mouse brain (Iwata et al, 2025). More generally, future work will address whether reduced NAD^+^ availability may limit the activities of enzymes that depend on NAD^+^ as a cofactor or use NAD+ as a co-substrate, and whether the resulting metabolic perturbations affect developmental speed in the *Drosophila* eye.

Finally, we note that the control of cellular metabolism involves a myriad of intricated regulatory feedback mechanisms which cannot be easily dissected using classical genetic approaches, e.g., epistasis. However, gene-based perturbation approaches are useful to test and identify a wide range of metabolic perturbations associated with a change in developmental speed. Our study showed that this approach is useful to reveal key compensatory mechanisms and to thereby identify key metabolic processes that become limiting under these perturbed conditions, e.g., NAD^+^ regeneration in the case of ETC perturbations, and which can be viewed as key regulatory nodes in the regulation of developmental speed.

## Methods

**Table:** Reagents and tools table

Reagents/resource	Reference or source	Identifier or catalog number
**Experimental models**		
*UAS-ND-42 RNAi: y[1] v[1]; P{y[+t7.7] v[+t1.8]=TRiP.HM05104}attP2 (Drosophila melanogaster)*	Bloomington Drosophila Stock Center	BL-28894
*UAS-Naprt RNAi: y[1] v[1]; P{y[+t7.7] v[+t1.8]=TRiP.HMJ23364}attP40 (Drosophila melanogaster)*	Bloomington Drosophila Stock Center	BL-61875
*UAS-Sarm RNAi: y[1] v[1]; P{y[+t7.7] v[+t1.8]=TRiP.HMJ30091}attP40 (Drosophila melanogaster)*	Bloomington Drosophila Stock Center	BL-63525
*UAS-Sirt1 RNAi: y[1] v[1]; P{y[+t7.7] v[+t1.8]=TRiP.HMJ21708}attP40 (Drosophila melanogaster)*	Bloomington Drosophila Stock Center	BL-53697
UAS-Parp1 RNAi: y[1] sc[*] v[1] sev[21]; P{y[+t7.7] v[+t1.8]=TRiP.HMC04658}attP40 (Drosophila melanogaster)	Bloomington Drosophila Stock Center	BL-57265
UAS-chico RNAi: y[1] sc[*] v[1] sev[21]; P{y[+t7.7] v[+t1.8]=TRiP.HMS01553}attP2/TM3, Sb[1] (Drosophila melanogaster)	Bloomington Drosophila Stock Center	BL-36665
*UAS-NDI1: w[1118]; P{w[+mC]=UAS-NDI1.S}A46 (Drosophila melanogaster)*	Bloomington Drosophila Stock Center	BL-93878
*UAS-Ldh RNAi: y[1] v[1]; P{y[+t7.7] v[+t1.8]=TRiP.HMS00039}attP2 (Drosophila melanogaster)*	Bloomington Drosophila Stock Center	BL-33640
*UAS-ND-B12 RNAi: y[1] v[1]; P{y[+t7.7] v[+t1.8]=TRiP.HMJ23156}attP40 (Drosophila melanogaster)*	Bloomington Drosophila Stock Center	BL-61321
*mirrDE (Drosophila melanogaster)*	Bloomington Drosophila Stock Center	BL-29650
*UAS-mIFP-T2A-HO1* (*Drosophila melanogaster*)	Bloomington Drosophila Stock Center	BL-64181
*P{w[+mC]=UASp-VenusFoxo}2(Drosophila melanogaster)*	Bloomington Drosophila Stock Center	BL-44214
*P{ry[+t7.2]=ey-FLP.N}5(Drosophila melanogaster)*	Bloomington Drosophila Stock Center	BL-5576
*P{y[+t7.7] v[+t1.8]=VALIUM20-EGFP.shRNA.3}attP40 (Drosophila melanogaster)*	Bloomington Drosophila Stock Center	BL-5702
*UAS-Got2 (Drosophila melanogaster)*	Rutter lab, PMID: 41400249	N/A
*w;;ato[GFP] KI (Drosophila melanogaster)*	Hassan lab, PMID: 29576424	N/A
*tubP-Gal4 tubP-Gal80ts (Drosophila melanogaster)*	This study	N/A
*mirr-Gal4 AtoGFP P[mw, tubP-GAL80[ts]]7 (Drosophila melanogaster)*	This study	N/A
*hs-flp/+;; mirr-Gal4 ato5’-FRT-mCherrynls-FRT-GFPnls/+ (Drosophila melanogaster)*	This study	N/A
*PBac{y[+], pUbi-Peredox-mCherrynls attP-3B}VK31 (62E1) (Drosophila melanogaster)*	This study	N/A
*PBac{y[+], pUbi-ATPSnFR-95A.119L-mScarlet attP}VK31 (62E1) (Drosophila melanogaster)*	This study	N/A
*PBac{y[+], pUAS-cyto-LbNOX attP-3B}VK37 (22A3) (Drosophila melanogaster)*	This study	N/A
*PBac{y[+], pUAS-mito-LbNOX attP-3B}VK37 (22A3) (Drosophila melanogaster)*	This study	N/A
*PBac{y[+], ato5’-FRT-mCherrynls-FRT-GFPnls attP-3B}VK31 (62E1) (Drosophila melanogaster)*	This study	N/A
*PBac{y[+], pUbi-mito-SoNar attP-3B}VK31 (62E1) (Drosophila melanogaster)*	This study	N/A
*PBac{y[+], pUbi-cyto-SoNar attP-3B}VK31 (62E1) (Drosophila melanogaster)*	This study	N/A
PBac{y[+], ato5'-ND-42* attP-3B}VK37 (22A3) PBac{y[+], ato3'-ND-42* attP-3B}VK02 (28E7) (Drosophila melanogaster)	This study	N/A
PBac{y[+],pUbi-ND42* attP-3B}VK01 (59D3) (Drosophila melanogaster)	This study	N/A
*PBac{y[+], m4P-FRT-stop-FRT-VenusFoxo attP-3B}VK05 (75A10) (Drosophila melanogaster)*	This study	N/A
**Reagents, chemicals, enzymes, and other reagents**		
Nicotinamide riboside chloride	Sigma	SMB00907
β-Nicotinamide mononucleotide	Sigma	N3501
n-propyl gallate	Sigma	P3130
Trypan blue	Gibco	15250-061
Grace’s medium	Sigma	G9771
Penicillin/Streptomycin 0.5%	Sigma	P4333
20-Hydroxyecdysone	Sigma	H5142
Fibrinogen	Sigma	11424246
Thrombin	Sigma	11407522
Bromopyruvic acid	Sigma	16490
DTAB	Sigma	D8638
NADH	Sigma	N8129
NAD^+^	Sigma	N7004
1:1000 Click-iT OPP reagent	Invitrogen	C10458
NAD^+/^NADH-Glo assay	Promega	G9071
Pierce BCA assay kit	Fisher Scientific	18064090
Mini-PROTEAN TGX Stain-Free Gels, 8-16%	Bio-Rad	4568103
Nitrocellulose membranes	Bio-Rad	1620112
Antimycin A	Sigma	A8674
**Antibodies**		
Goat anti-GFP	Abcam	ab5450
Rabbit anti-dsRed	Takara	NC9580775
Rabbit anti-pH3	Upstate	H0412
Cy3 anti-rat	Jackson Lab	712165150
Cy3 anti-rabbit	Jackson Lab	711165152
Cy2 anti-goat	Jackson Lab	705545147
Cy5 anti-rat	Jackson Lab	712165153
Anti-Puromycin coupled to AF-647	Millipore	MABE343-AF647
Rabbit anti-poly-ADPr	Millipore	MABE1031, RRID: AB_2665467
Rabbit anti-H3K18ac	Abcam	ab1191
Rabbit anti-pan-acetylation (1D5F2)	Proteintech	66289-1-Ig
Rabbit anti-Fibrillarin	Abcam	ab5821
Anti-rabbit StarBright 700	Bio-Rad	12004161
**Others**		
Chamlide magnetic imaging chamber	LCI	CM-B25-1
Tecan INFINITE 200 PRO	Tecan	N/A
HESI II probe	Thermo Fisher	N/A
QExactive Plus Orbitrap	Thermo Fisher	N/A
Dionex UltiMate 3000 UPLC system	Thermo Fisher	N/A
ZIC-pHILIC column with guard column	Millipore	20 mm × 2.1 mm; i.d. 5 µm
Bio-Rad Chemi-Doc MP Imager	Bio-Rad	N/A
Zeiss LSM780 microscope	Zeiss	N/A
×20 Objective	Zeiss	PL APO, N.A. 0.8
×40 Objective	Zeiss	PL APO, N.A. 1.32
Nikon Ti2-E AX-NSPARC	Nikon	N/A
×40 Objective	Nikon	PL APO, N.A. 1.25
**Software, R packages**		
Thermo TraceFinder software	Thermo Fisher	N/A
Thermo Xcalibur software	Thermo Fisher	N/A
MetaboAnalyst v6.0	PMID: 38693118	N/A
Bio-Rad Image Lab software	Bio-Rad	N/A
Seurat package (v5.1.0)	PMID: 37231261, PMID: 25867923	N/A
EnrichR package	PMID: 23586463, PMID: 27141961, PMID: 33780170	N/A
Calculate Pathway Scores for Each Cell in scRNA-Seq Data (SiPSiC) R-package	PMID: 38981682	N/A
Metabolic Pathway Report in Flybase	Flybase	N/A
Fiji	PMID: 22743772	https://fiji.sc
ImageJ	PMID: 22743772	https://imagej.net/ij
R Studio v R4.4.2	R Core Team	https://www.R-project.org/

### Flies

A *mirr-Gal4* driver, *mirr*^*DE*^, was used to drive gene-based perturbations in *d* cells. To easily mark the differentiation front, *ato*^*GFP*^ was recombined with *mirr*^*DE*^ [*ato*^*GFP*^ is a functional GFP knock-in allele of *ato* (Mora et al, 2018)]. Unless noted otherwise, all crosses and flies were maintained at 25 °C for analysis. Tested lines were obtained from the Vienna Drosophila Resource Center (VDRC) and the Bloomington Drosophila Stock Center (BDSC) for flies (see Dataset EV1). The UAS-Got2 line was from the Rutter lab (Toshniwal et al, 2025). The MF phenotypes were scored in eye discs with 20 + /−10 rows of ommatidia.

Conditional silencing was achieved at 29 °C for 3 days using a *mirr-Gal4 tubP-Gal80*^*ts*^ *ato*^*GFP*^ driver for MF delay analysis, and a *tubP-Gal4 tubP-Gal80*^*ts*^ driver for NAD^+^/NADH-glo assay analysis as well as for the LC/MS experiments. NMN (β-Nicotinamide mononucleotide, N3501, Sigma) and NR (Nicotinamide riboside chloride, SMB00907, Sigma) were dissolved in water and added to the fly food (NMN, 25 mM; NR, 5 mM) before introducing first (L1) and second (L2) instar larvae. After 3-4 days at 25 °C, mid-L3 larvae were collected for analysis. The *UAS-mIFP-T2A-HO1* transgene (BL-64181) that encodes an infrared marker was recombined with *mirr*^*DE*^, Peredox sensor, and SoNar sensor analysis was performed using this driver line.

The speed of progression of the MF was measured in eye discs dissected from *hs-flp*/+;; *mirr-Gal4 ato5’-FRT-mCherrynls-FRT-GFPnls*/+ third instar larvae (L3), which were subjected to a mild heat-shock (hs; 36 °C for 30 min) to induce flp-out recombination and then dissected after a chase period of 4 to 16 h post-hs. The following transgenic lines were obtained by phiC31-mediated integration: *PBac{y[+], pUbi-Peredox-mCherrynls attP-3B}VK31 (62E1); PBac{y[+], pUbi-ATPSnFR-95A.119L-mScarlet attP}VK31 (62E1); PBac{y[+], pUAS-cyto-LbNOX attP-3B}VK37 (22A3); PBac{y[+], pUAS-mito-LbNOX attP-3B}VK37 (22A3); PBac{y[+], ato5’-FRT-mCherrynls-FRT-GFPnls attP-3B}VK31 (62E1), PBac{y[+], pUbi-cyto-SoNar}VK31 (62E1), PBac{y[+], pUbi-mito-SoNar attP-3B}VK31 (62E1)*, PBac{y[+],ato5'-ND-42* attP-3B}VK37 (22A3), PBac{y[+],ato3'-ND-42* attP-3B}VK02 (28E7), PBac{y[+], pUbi-ND42* attP-3B}VK37 (22A3), PBac{y[+], m4P-FRT-stop-FRT-VenusFoxo attP-3B}VK05 (75A10). The ato3’/5’-ND-42* chromosome used in Fig. 6 was generated by recombination between the ato5'-ND-42* and ato3'-ND-42* transgenes. Injection was performed by BestGene Inc. (Chinmo, USA). All new fly lines are available upon request.

Flies were grown on a rich medium: corn flour (60 g/L), yeast extract (60 g/L), agar (6 g/L), nipagin (4 g/L), propionic acid (0.4%).

### Transgenes

The nuclear Peredox NADH/NAD^+^ ratio sensor was obtained by cloning a PCR-amplified fragment encoding the Peredox-mCherrynls sensor (Addgene #3238) into a pUbi-attB vector using Gibson Assembly. The Cyto-SoNar sensor was obtained by cloning a PCR-amplified fragment encoding SoNar obtained from the genome of UAS-SoNar flies (Bonnay et al, 2020) into a pUbi-attB vector using Gibson Assembly. The mito-SoNar sensor was similarly obtained, adding 2 N-terminal copies of the mitochondria targeting signal from the human *cox8A* gene and a GDPPVAT linker which were obtained by the annealing of two partially overlapping oligonucleotides followed by PCR amplification. The ATP sensor was similarly obtained using a PCR-amplified fragment encoding the iATPsnFR-A95A.A119L.mScarlet sensor (Plasmid is available at Janelia Farm: https://janeliamaterials-constructs.azurewebsites.net/) (Marvin et al, 2024). A 1986 nucleotide (nt)-long fragment starting 5’ to the ATG of the *Ubi-p63E* gene (*CG11624*) and encoding its promoter was used to direct the constitutive expression of a Peredox-mCherrynls fusion protein.

The UAS-cyto-LbNOX plasmid was obtained by cloning a PCR-amplified fragment encoding cyto-LbNOX (Addgene #7528) into a pUAS-attB vector using Gibson Assembly. A short DNA fragment encoding one copy of the mitochondria targeting signal from the human *cox8A* gene and a GDPPVAT linker was obtained via the annealing of two partially overlapping oligonucleotides, followed by PCR amplification, and was cloned into the UAS-cyto-LbNOX plasmid by Gibson assembly to generate the UAS-mito-LbNOX transgene.

To measure the speed of progression of the front, we designed a recombination-based mCherry-to-GFP switch plasmid, noted ato5'-FRT-mCherrynls-FRT-GFPnls. This plasmid, produced in three Gibson assembly steps, consists in the ato5’Eye enhancer of the *atonal* (*ato*) gene (a 2918 nt fragment located 7471 nt 5’ to the *ato* start codon), the *ato* basal promoter (a 1058 nt fragment located 58 nt 5’ to the *ato* start codon), a FRT-mCherrynls-hsp70 stop-FRT cassette followed by a GFPnls obtained from pattB-neur-GFPnls-SV403’UTR plasmid (Aerts et al, 2010).

A RNAi-resistant ND-42 gene, ND-42*, was obtained by recoding the 300nt fragment targeted by dsRNA-HM05104 using gene synthesis (Integrated DNA Technologies). A DNA fragment encoding ND-42* was PCR-amplified to produce the pUbi-ND-42* transgene, that directs the constitutive expression of ND-42* under the control of the *Ubi-p63E* promoter, and the ato3’-ND-42*, that direct the expression of ND-42* under the control of an ato3’ enhancer (a 1828 nt DNA fragment located 2824 nt 3’ to the *ato* stop codon (Couturier et al, 2026)). The two plasmids above were obtained by Gibson assembly. The m4P-FRT-stop-FRT-VenusFoxo Bacterial Artificial Chromosome (BAC) transgene, expressing Venus-tagged Foxo under the control of the regulatory sequences of the *E(spl)m4-BFM* (*m4*) gene, was obtained by Gibson assembly and BAC recombineering in *E. coli*. Starting with a BAC encoding a 6078 nt genomic fragment encoding the m4 gene, including 4345 nt of upstream sequence, a FRT-stop-FRT fragment was first introduced in the 5’UTR, then the *m4* Open Reading Frame (ORF) was replaced by the Venus-Foxo sequence, which was PCR-amplified from DNA extracted from BL-44214 flies.

All DNA constructs produced by PCR and/or Gibson assembly were verified by sequencing. Primers and cloning details are available upon request.

### smiFISH

smiFISH probes were prepared as previously described (Tsanov et al, 2016). In short, FLAP-X oligonucleotide were annealed with the smiFISH probe set mix in Tris-HCl 50 mM pH = 7.5, NaCl 100 mM, MgCl2 10 mM using a thermocycler (85 °C, 3 min; 65 °C, 3 min; 25 °C 3 min). smi FISH probes were designed using Stellaris Inc., probe designer. The sequence of each probe set is available upon request. FISH was performed as described in (Trcek et al, 2017). Briefly, brain complexes were dissected from third instar larvae in phosphate-buffered saline (PBS) and fixed in 4% paraformaldehyde (in PBS) for 20 min at room temperature (RT). After, samples were permeabilized using 0.5% Triton-X in PBS and transferred into 4 M Urea in 2×SSC. Then the samples were hybridized at 37 °C overnight in SSC 2×, Urea 4 M, Dextrane 10%, Vanydyl complex 10 mM, 0.15 mg/ml salmon sperm DNA, 100 μM smiFISH probe and anti-GFP (1:1000) and rinsed with 4 M Urea in 2×SSC and after with 2×SSC. The secondary antibody was incubated 2 h in PBS with 0.1% Triton-X in (PBT) at RT.

### Immunostaining

Dissected brain complexes were fixed in 4% paraformaldehyde (in PBS) for 20 min, washed in PBT, incubated 1–2 h at RT in PBT with first antibodies, washed 3 × 10 min in PBT, incubated 1–2 h at RT with secondary antibodies, washed with PBT, and mounted in mounting medium (90% glycerol in PBS, 0.1% n-propyl gallate, Sigma P3130). The following primary antibodies were used: rat anti-Ecad (DSHB, 1:100); goat anti-GFP (Abcam, ab5450, 1:1000); rabbit anti-dsRed (Takara, 1:1000); rabbit anti-pH3 (Upstate, 1:1000). The following secondary antibodies were used: Cy3 anti-rat (1:1000); Cy3 anti-rabbit (1:1000); Cy2 anti-goat (1:1000); Cy5 anti-rat (1:1000). Secondary antibodies were from Jackson Laboratories. After incubation, samples were briefly washed with PBT, followed by three 10 min washes in 0.1% PBT. Samples were calibrated in 50% Glycerol/PBS, before eye-antennal imaginal discs were dissected and mounted in mounting media (10% PBS 10X, 90% glycerol (ACS grade 99-100% purity), 0.1% n-propyl gallate (20% (w /v), Sigma P3130).

### Ex vivo culturing of eye discs

Third instar larvae were briefly washed in water, rinsed in PBS 1× and dissected in Grace’s medium (Sigma G9771) at pH 6.7 supplemented with 5% fetal bovine serum (FBS), Penicillin/Streptomycin 0.5% (Sigma P4333) and 20 nM 20-Hydroxyecdysone (Sigma H5142). Eye-antenna imaginal discs were transferred into a magnetic imaging chamber (Chamlide CM-B25-1, LCI) with a drop of culture medium. Discs were then embedded using a fibrinogen-thrombin mix (Sigma 11424246 and 11407522), then cultured 20 min at 25 °C in supplemented Grace’s medium before imaging at 23–25 °C. Antimycin A (10 µM in 99% EtOH) and bromopyruvic acid (3BP, 100 µM in imaging medium) were added to the medium directly in the imaging chamber.

### Analysis of the protein synthesis rate

Third instar larvae were rinsed in PBS 1× and dissected in supplemented Grace’s medium (as described above). Eye-antenna discs were transferred into fresh medium containing 1:1000 Click-iT OPP reagent (Invitrogen, Click-iT^Tm^ Plus OPP). Discs were incubated at RT under constant shaking for 10 min and, after a quick rinse with 1×PBS, fixed for 20 min in 4% PFA. After permeabilization with 0.5% Triton X-100 in PBS, incorporated Puromycin was detected using mouse anti-Puromycin coupled to AF-647 (1:1000, Millipore, MABE343-AF647). After incubation, samples were briefly washed with PBT, followed by three 10 min washes in 0.1% PBT, and mounted as described above.

### Microscopy

Images were acquired on a confocal Zeiss LSM780 microscope equipped with ×20 (PL APO, N.A. 0.8) and ×40 (PL APO, N.A. 1.32) objectives and a Nikon Ti2-E AX-NSPARC microscope with a ×40 (PL APO, N.A. 1.25) objective. The iATPsnFR sensor was excited using the 488 and 561 nm lasers, and the emitted light was collected in the 490–534 and 579–641 nm intervals. The Peredox-mCherrynls sensor was excited using the 405 and 561 nm lasers, and the emitted light was collected between 409–540 nm and 597–659 nm, respectively. SoNar was excited using 488 nm and 564 nm laser and light was collected around 530 nm. The signals of these metabolic sensors were measured in UPs and DCs over user-defined regions of interest on average z-projected images using ImageJ.

Scanning electron microscopy (SEM) was performed on dehydrated adult flies coated with gold palladium following standard procedures.

### NAD^+^/NADH-glo assay

Total cellular NADH and NAD^+^ were measured using NAD^+/^NADH-glo assay (Promega). Around 40 eye discs were dissected in PBS and lysed using 1% DTAB (Sigma, D8638) in base solution (0.2 N NaOH). Lysate fractions corresponding to 5 and 10 eye discs were pipetted into a 96-well plate (Corning 96 Flat Bottom white Polystyrene). The remaining lysate was stored at -20 °C and used for protein quantification, in which a standard curve was included for each experiment (BCA assay, Pierce). The manufacturer’s instructions were followed thereafter. Briefly, 50 µl of the lysate was then transferred to a second well containing 25 µl of 0.4 M HCl (for NAD+ measurement) or to an empty well (for NADH measurement). Both samples were heated at 60 °C for 15 min to deplete NAD+ or NADH. Samples were then left on the bench for 10 min to cool down to room temperature before adding neutralizing solutions (25 µl of 0.5 M Tris base to the NAD+ sample, and 50 µl of 1:1 0.4 M HCl:0.5 M Tris base to the NADH sample), followed by 100 µl of NADH-Glo detection reagent. Following incubation for 1 h at room temperature, the produced luminescence signal was measured on a microplate reader (Tecan INFINITE 200 PRO). Absolute concentrations of NADH and NAD+ were determined against standard curves using NADH (Sigma, N8129) and NAD^+^ (Sigma N7004). Measured concentrations were normalized by protein concentration values.

### Targeted LC/MS metabolomics analyses

For each sample, eye-antenna discs from 30 female larvae were rapidly dissected in PBS at 4 °C and frozen using dry ice. Metabolites were extracted using 0.1 mL of a solution composed of 50% methanol, 30% acetonitrile (ACN), and 20% water. Samples were then vortexed for 5 min at 4 °C and centrifuged at 16,000× *g* for 15 min at 4 °C. The supernatants were collected and stored at −80 °C until analysis. LC/MS analyses were conducted on a QExactive Plus Orbitrap mass spectrometer equipped with an Ion Max source and a HESI II probe coupled to a Dionex UltiMate 3000 UPLC system (Thermo Fisher). The 5 µl samples were injected onto a ZIC-pHILIC column (150 mm × 2.1 mm; i.d. 5 µm) with a guard column (20 mm × 2.1 mm; i.d. 5 µm) (Millipore) for LC separation. Buffer A was 20 mM ammonium carbonate, 0.1% ammonium hydroxide (pH 9.2), and buffer B was ACN. The chromatographic gradient was run at a flow rate of 0.2 µl min−1 as follows: 0–20 min, linear gradient from 80% to 20% of buffer B; 20–20.5 min, linear gradient from 20 to 80% of buffer B; 20.5–28 min, 80% buffer B. The mass spectrometer was operated in full scan, polarity switching mode with the spray voltage set to 2.5 kV and the heated capillary held at 320 °C. The sheath gas flow was set to 20 units, the auxiliary gas flow to 5 units, and the sweep gas flow to 0 units. The metabolites were detected across a mass range of 75–1000 *m/z* at a resolution of 35,000 (at 200 *m/z*) with the automatic gain control target at 106 and the maximum injection time at 250 ms. Lock masses were used to ensure mass accuracy below 5 ppm. Data were acquired with Thermo Xcalibur software (Thermo Fisher). The peak area of metabolites was determined using Thermo TraceFinder software (Thermo Fisher), identified by the exact mass of each singly charged ion and by the known retention time on the HPLC column. To perform protein normalization, the remaining protein pellet was diluted in lysis buffer (1% DTAB (Sigma, D8638) in base solution (0.2 N NaOH)) and used for protein quantification (BCA assay, Pierce). Data was analyzed using the MetaboAnalyst v6.0 (Pang et al, 2024).

### Western blot analysis

Eye-antenna discs from 20 female larvae were lysed using NuPAGE LDS sample buffer (Invitrogen). Inhibitors of PARG (1 μM; PDD00017273, Sigma), and PARP activities (1 μM; Olaparib, LKT LABS) were added in the lysis buffer. Following denaturation (100 °C for 10 min), proteins were separated on Mini-PROTEAN TGX Stain-Free Gels, 8-16% (Bio-Rad) and transferred to nitrocellulose membranes (Bio-Rad) overnight at 4 °C. The blotted membranes were blocked with 10% Bovine Serum Albumine (w/v) in Tris Buffer Saline (with 0.1% Tween 20 (TBS-Tw, 1 h at room temperature). The top (> 55 kDa) and bottom (< 35 kDa) parts of the membranes were incubated with rabbit anti-poly-ADPr (MABE1031, Millipore, 1:1000, RRID: AB_2665467), rabbit anti-H3K18ac (ab1191, Abcam, 1:1000) and rabbit anti-pan-acetylation (1D5F2, Proteintech, 66289-1-Ig, 1:1000), while the central part (35-55 kDa) was incubated with rabbit anti-Fibrillarin (ab5821, Abcam, 1:1000) for 1 h at room temperature. After washing in TBS-Tw, blots were incubated with anti-rabbit StarBright 700 (Bio-Rad). Signals were detected using a Bio-Rad Chemi-Doc MP Imager and analyzed with the Bio-Rad Image Lab software.

### scRNAseq

Brain complexes from Oregon-R and *mirr* > *ND-42*^*RNAi*^ larvae were dissected in Dulbecco’s phosphate-buffered saline (DPBS; ThermoFisher) and then dissociated in 0.25% trypsin-EDTA solution to a single-cell suspension at 37 °C for 10 min. These cells were then washed with DPBS twice and passed through a 35μm filter before library preparation. Construction of 10x single-cell libraries and sequencing on the Illumina Hiseq platform were performed by Novogene. Raw data mapping was performed using the standard Cell Ranger pipeline (v 2.2.0) to generate UMI count matrices. The NCBI GEO accession number for this dataset is GSE294725. We performed read alignment and annotation using the BDGP6 genome reference fastaq file and the BDGP6.91.gtf file.

The cluster comprising eye field cells was identified based on eya expression. Cells from this cluster were further clustered using the Seurat package (v5.1.0) (Hao et al, 2021; Satija et al, 2015), and peripodial cells, UPs, and DCs were identified based on previously described markers (Bravo González-Blas et al, 2020; Yeung et al, 2022). UPs and DCs were further annotated as v and d cells based on the expression of the *mirr*, *ara* and/or *caup* genes. Differentially expressed genes (DEGs) were defined using the FindMarkers function. The d vs v DEGs found in Oregon-R (control) were removed from the list of DEGs found in *mirr* > *ND-42*^*RNAi*^ eye discs. Differentially expressed transposable elements were processed separately. GO term analysis was performed using the EnrichR package (Chen et al, 2013; Kuleshov et al, 2016; Xie et al, 2021) using the “GO_Biological_Process_2018” database. The Calculate Pathway Scores for Each Cell in scRNA-Seq Data (SiPSiC) R-package was used to assign a pathway score to each cell (Davis et al, 2024). Gene members of each pathway were identified using the Metabolic Pathway Report in Flybase. Differences in pathway scores were considered significant for a Cohen’s d effect size (noted s herein) superior to 0.2 (Sullivan and Feinn, 2012).

### Design and statistics

For the RNAi perturbation screen, we found that a sample size of ~5 discs was sufficient to identify positive hits. For scRNAseq and metabolomics, the sample size was determined based on cost and time (triplicates were studied). For phenotypic analysis, >10 discs per condition were studied (all sample sizes indicated in the legends). We performed blind counting for key experiments, as indicated in the legends. All replicates were biological in nature. Measured values, including MF delay values, are reported as mean + /− standard deviation. Statistics was performed using R Studio; all tests are indicated in the legends.

## Supplementary information

Peer Review File

Dataset EV1

Dataset EV2

Dataset EV3

Dataset EV4

Source data Fig. 1

Source data Fig. 2

Source data Fig. 3

Source data Fig. 4

Source data Fig. 5

Source data Fig. 6

Source data Fig. 7

Expanded View Figures

## Data Availability

RNA-Seq data: Gene Expression Omnibus GSE294725. The source data of this paper are collected in the following database record: biostudies:S-SCDT-10_1038-S44318-026-00801-4.

## References

[CR1] Abdellatif M, Bugger H, Kroemer G, Sedej S (2022) NAD+ and vascular dysfunction: from mechanisms to therapeutic opportunities. J Lipid Atheroscler 11:111–132 35656147 10.12997/jla.2022.11.2.111 PMC9133775

[CR2] Aerts S, Quan X-J, Claeys A, Naval Sanchez M, Tate P, Yan J, Hassan BA (2010) Robust target gene discovery through transcriptome perturbations and genome-wide enhancer predictions in Drosophila uncovers a regulatory basis for sensory specification. PLoS Biol 8:e1000435 20668662 10.1371/journal.pbio.1000435 PMC2910651

[CR3] Beltrà M, Pöllänen N, Fornelli C, Tonttila K, Hsu MY, Zampieri S, Moletta L, Corrà S, Porporato PE, Kivelä R et al (2023) NAD+ repletion with niacin counteracts cancer cachexia. Nat Commun 14:1849 37012289 10.1038/s41467-023-37595-6 PMC10070388

[CR4] Birsoy K, Wang T, Chen WW, Freinkman E, Abu-Remaileh M, Sabatini DM (2015) An essential role of the mitochondrial electron transport chain in cell proliferation is to enable aspartate synthesis. Cell 162:540–551 26232224 10.1016/j.cell.2015.07.016 PMC4522279

[CR5] Bonnay F, Veloso A, Steinmann V, Köcher T, Abdusselamoglu MD, Bajaj S, Rivelles E, Landskron L, Esterbauer H, Zinzen RP et al (2020) Oxidative metabolism drives immortalization of neural stem cells during tumorigenesis. Cell 182:1490–1507.e19 32916131 10.1016/j.cell.2020.07.039

[CR6] Bravo González-Blas C, Quan X, Duran-Romaña R, Taskiran II, Koldere D, Davie K, Christiaens V, Makhzami S, Hulselmans G, de Waegeneer M et al (2020) Identification of genomic enhancers through spatial integration of single-cell transcriptomics and epigenomics. Mol Syst Biol 16:e9438 32431014 10.15252/msb.20209438 PMC7237818

[CR7] Brenner C (2022) Viral infection as an NAD+ battlefield. Nat Metab 4:2–3 34980922 10.1038/s42255-021-00507-3 PMC10155260

[CR8] Buttgereit F, Brand MD (1995) A hierarchy of ATP-consuming processes in mammalian cells. Biochem J 312(Pt 1):163–167 7492307 10.1042/bj3120163 PMC1136240

[CR9] Cambronne XA, Stewart ML, Kim D, Jones-Brunette AM, Morgan RK, Farrens DL, Cohen MS, Goodman RH (2016) Biosensor reveals multiple sources for mitochondrial NAD. Science 352:1474–1477 27313049 10.1126/science.aad5168 PMC6530784

[CR10] Cassidy JJ, Bernasek SM, Bakker R, Giri R, Peláez N, Eder B, Bobrowska A, Bagheri N, Nunes Amaral LA, Carthew RW (2019) Repressive gene regulation synchronizes development with cellular metabolism. Cell 178:980–992.e17 31353220 10.1016/j.cell.2019.06.023 PMC6865806

[CR11] Chen EY, Tan CM, Kou Y, Duan Q, Wang Z, Meirelles GV, Clark NR, Ma’ayan A (2013) Enrichr: interactive and collaborative HTML5 gene list enrichment analysis tool. BMC Bioinforma 14:128 10.1186/1471-2105-14-128 PMC3637064 23586463

[CR12] Cho J, Hur JH, Graniel J, Benzer S, Walker DW (2012) Expression of yeast NDI1 rescues a Drosophila complex I assembly defect. PLoS ONE 7:e50644 23226344 10.1371/journal.pone.0050644 PMC3511326

[CR13] Couturier L, Luna-Escalante J, Mazouni K, Mestdagh C, Phan M-S, Tinevez J-Y, Schweisguth F, Corson F (2026) Pulsatile dynamics propagate crystalline order in the developing Drosophila eye. Dev Cell 61:372–384 41192428 10.1016/j.devcel.2025.10.007

[CR14] Dai Z, Ramesh V, Locasale JW (2020) The evolving metabolic landscape of chromatin biology and epigenetics. Nat Rev Genet 21:737–753 32908249 10.1038/s41576-020-0270-8 PMC8059378

[CR15] Davis D, Wizel A, Drier Y (2024) Accurate estimation of pathway activity in single cells for clustering and differential analysis. Genome Res 34:925–936 38981682 10.1101/gr.278431.123 PMC11293543

[CR16] Diaz-Cuadros M, Miettinen TP, Skinner OS, Sheedy D, Díaz-García CM, Gapon S, Hubaud A, Yellen G, Manalis SR, Oldham WM et al (2023) Metabolic regulation of species-specific developmental rates. Nature 613:550–557 36599986 10.1038/s41586-022-05574-4 PMC9944513

[CR17] Dietzl G, Chen D, Schnorrer F, Su K-C, Barinova Y, Fellner M, Gasser B, Kinsey K, Oppel S, Scheiblauer S et al (2007) A genome-wide transgenic RNAi library for conditional gene inactivation in Drosophila. Nature 448:151–156 17625558 10.1038/nature05954

[CR18] Ebisuya M, Briscoe J (2018) What does time mean in development? Development 145:dev164368 29945985 10.1242/dev.164368 PMC6031406

[CR19] Feng S, Zacharioudaki E, Millen K, Bray SJ (2020) The SLC36 transporter Pathetic is required for neural stem cell proliferation and for brain growth under nutrition restriction. Neural Dev 15:10 32741363 10.1186/s13064-020-00148-4 PMC7398078

[CR20] Figley MD, Gu W, Nanson JD, Shi Y, Sasaki Y, Cunnea K, Malde AK, Jia X, Luo Z, Saikot FK et al (2021) SARM1 is a metabolic sensor activated by an increased NMN/NAD+ ratio to trigger axon degeneration. Neuron 109:1118–1136.e11 33657413 10.1016/j.neuron.2021.02.009 PMC8174188

[CR21] Figueroa-Clarevega A, Bilder D (2015) Malignant Drosophila tumors interrupt insulin signaling to induce cachexia-like wasting. Dev Cell 33:47–55 25850672 10.1016/j.devcel.2015.03.001 PMC4390765

[CR22] Ganapathy-Kanniappan S, Geschwind J-FH, Kunjithapatham R, Buijs M, Vossen JA, Tchernyshyov I, Cole RN, Syed LH, Rao PP, Ota S et al (2009) Glyceraldehyde-3-phosphate dehydrogenase (GAPDH) is pyruvylated during 3-bromopyruvate mediated cancer cell death. Anticancer Res 29:4909–4918 20044597 PMC3743725

[CR23] Ghosh S, Körte A, Serafini G, Yadav V, Rodenfels J (2023) Developmental energetics: Energy expenditure, budgets and metabolism during animal embryogenesis. Semin Cell Dev Biol 138:83–93 35317962 10.1016/j.semcdb.2022.03.009

[CR24] Gillooly JF, Charnov EL, West GB, Savage VM, Brown JH (2002) Effects of size and temperature on developmental time. Nature 417:70–73 11986667 10.1038/417070a

[CR25] Gossmann TI, Ziegler M, Puntervoll P, de Figueiredo LF, Schuster S, Heiland I (2012) NAD+ biosynthesis and salvage—a phylogenetic perspective. FEBS J 279:3355–3363 22404877 10.1111/j.1742-4658.2012.08559.x

[CR26] Greenwood S, Struhl G (1999) Progression of the morphogenetic furrow in the Drosophila eye: the roles of hedgehog, decapentaplegic and the Raf pathway. Development 126:5795–5808 10572054 10.1242/dev.126.24.5795

[CR27] Hao Y, Hao S, Andersen-Nissen E, Mauck WM, Zheng S, Butler A, Lee MJ, Wilk AJ, Darby C, Zager M et al (2021) Integrated analysis of multimodal single-cell data. Cell 184:3573–3587.e29 34062119 10.1016/j.cell.2021.04.048 PMC8238499

[CR28] Hayashi Y, Hino S, Sato T, Kashio S, Otsubo K, Saito K, Sato B, Kawano N, Saito D, Miura M et al (2025) Repressive S-adenosylmethionine biosynthesis status inhibits transcription of HeT-A retrotransposon in the germline of Drosophila. J Biochem 178:217–228 40599133 10.1093/jb/mvaf041

[CR29] Hosios AM, Vander Heiden MG (2018) The redox requirements of proliferating mammalian cells. J Biol Chem 293:7490–7498 29339555 10.1074/jbc.TM117.000239 PMC5961062

[CR30] Hu Q, Wu D, Walker M, Wang P, Tian R, Wang W (2021) Genetically encoded biosensors for evaluating NAD+/NADH ratio in cytosolic and mitochondrial compartments. Cell Rep Methods 1:100116 34901920 10.1016/j.crmeth.2021.100116 PMC8659198

[CR31] Hung YP, Albeck JG, Tantama M, Yellen G (2011) Imaging cytosolic NADH-NAD(+) redox state with a genetically encoded fluorescent biosensor. Cell Metab 14:545–554 21982714 10.1016/j.cmet.2011.08.012 PMC3190165

[CR32] Iwata R, Casimir P, Erkol E, Boubakar L, Planque M, Gallego López IM, Ditkowska M, Gaspariunaite V, Beckers S, Remans D et al (2023) Mitochondria metabolism sets the species-specific tempo of neuronal development. Science 379:eabn4705 36705539 10.1126/science.abn4705

[CR33] Iwata R, Gallego-Lopez IM, Erkol E, Limame R, Vandekeere A, Benac N, Turner-Bridger B, Planque M, Ditkowska M, Lein V et al (2025) Species-specific rates of fatty acid metabolism set the scale of temporal patterning of corticogenesis through protein acetylation dynamics. Preprint at https://www.biorxiv.org/content/10.1101/2025.08.27.672586v1

[CR34] Jarman AP, Grell EH, Ackerman L, Jan LY, Jan YN (1994) Atonal is the proneural gene for Drosophila photoreceptors. Nature 369:398–400 8196767 10.1038/369398a0

[CR35] Kampjut D, Sazanov LA (2022) Structure of respiratory complex I–an emerging blueprint for the mechanism. Curr Opin Struct Biol 74:102350 35316665 10.1016/j.sbi.2022.102350 PMC7613608

[CR36] Kim W, Jang Y-G, Yang J, Chung J (2017) Spatial activation of TORC1 is regulated by hedgehog and E2F1 signaling in the Drosophila eye. Dev Cell 42:363–375.e4 28829944 10.1016/j.devcel.2017.07.020

[CR37] King MP, Attardi G (1989) Human cells lacking mtDNA: repopulation with exogenous mitochondria by complementation. Science 246:500–503 2814477 10.1126/science.2814477

[CR38] Klepsatel P, Girish TN, Gáliková M (2020) Acclimation temperature affects thermal reaction norms for energy reserves in Drosophila. Sci Rep 10:21681 33303846 10.1038/s41598-020-78726-z PMC7729904

[CR39] Kuleshov MV, Jones MR, Rouillard AD, Fernandez NF, Duan Q, Wang Z, Koplev S, Jenkins SL, Jagodnik KM, Lachmann A et al (2016) Enrichr: a comprehensive gene set enrichment analysis web server 2016 update. Nucleic Acids Res 44:W90–W97 27141961 10.1093/nar/gkw377 PMC4987924

[CR40] Kulkarni CA, Brookes PS (2019) Cellular compartmentation and the redox/nonredox functions of NAD. Antioxid Redox Signal 31:623–642 30784294 10.1089/ars.2018.7722 PMC6657305

[CR41] Lázaro J, Costanzo M, Sanaki-Matsumiya M, Girardot C, Hayashi M, Hayashi K, Diecke S, Hildebrandt TB, Lazzari G, Wu J et al (2023) A stem cell zoo uncovers intracellular scaling of developmental tempo across mammals. Cell Stem Cell 30:938–949.e7 37343565 10.1016/j.stem.2023.05.014 PMC10321541

[CR42] Li J, Pan J, Liu Y, Luo X, Yang C, Xiao W, Li Q, Yang L, Zhang X (2022) 3-Bromopyruvic acid regulates glucose metabolism by targeting the c-Myc/TXNIP axis and induces mitochondria-mediated apoptosis in TNBC cells. Exp Ther Med 24:520 35837063 10.3892/etm.2022.11447 PMC9257941

[CR43] Liu J, Xu Y, Stoleru D, Salic A (2012) Imaging protein synthesis in cells and tissues with an alkyne analog of puromycin. Proc Natl Acad Sci USA 109:413–418 22160674 10.1073/pnas.1111561108 PMC3258597

[CR44] Lobas MA, Tao R, Nagai J, Kronschläger MT, Borden PM, Marvin JS, Looger LL, Khakh BS (2019) A genetically encoded single-wavelength sensor for imaging cytosolic and cell surface ATP. Nat Commun 10:711 30755613 10.1038/s41467-019-08441-5 PMC6372613

[CR45] Luengo A, Li Z, Gui DY, Sullivan LB, Zagorulya M, Do BT, Ferreira R, Naamati A, Ali A, Lewis CA et al (2021) Increased demand for NAD+ relative to ATP drives aerobic glycolysis. Mol Cell 81:691–707.e6 33382985 10.1016/j.molcel.2020.12.012 PMC8315838

[CR46] Lynch M, Marinov GK (2015) The bioenergetic costs of a gene. Proc Natl Acad Sci USA 112:15690–15695 26575626 10.1073/pnas.1514974112 PMC4697398

[CR47] Ma C, Zhou Y, Beachy PA, Moses K (1993) The segment polarity gene hedgehog is required for progression of the morphogenetic furrow in the developing Drosophila eye. Cell 75:927–938 8252628 10.1016/0092-8674(93)90536-y

[CR48] Marvin JS, Kokotos AC, Kumar M, Pulido C, Tkachuk AN, Yao JS, Brown TA, Ryan TA (2024) iATPSnFR2: a high-dynamic-range fluorescent sensor for monitoring intracellular ATP. Proc Natl Acad Sci 121:e2314604121 38748581 10.1073/pnas.2314604121 PMC11126915

[CR50] Matsuda M, Hayashi H, Garcia-Ojalvo J, Yoshioka-Kobayashi K, Kageyama R, Yamanaka Y, Ikeya M, Toguchida J, Alev C, Ebisuya M (2020) Species-specific segmentation clock periods are due to differential biochemical reaction speeds. Science 369:1450–1455 32943519 10.1126/science.aba7668

[CR51] Matsuda M, Lázaro J, Ebisuya M (2025) Metabolic activities are selective modulators for individual segmentation clock processes. Nat Commun 16:845 39833174 10.1038/s41467-025-56120-5 PMC11746943

[CR52] McNeill H, Craig GM, Bateman JM (2008) Regulation of neurogenesis and epidermal growth factor receptor signaling by the insulin receptor/target of rapamycin pathway in Drosophila. Genetics 179:843–853 18505882 10.1534/genetics.107.083097 PMC2429878

[CR53] Mora N, Oliva C, Fiers M, Ejsmont R, Soldano A, Zhang T-T, Yan J, Claeys A, De Geest N, Hassan BA (2018) A temporal transcriptional switch governs stem cell division, neuronal numbers, and maintenance of differentiation. Dev Cell 45:53–66 29576424 10.1016/j.devcel.2018.02.023

[CR54] Nakanoh S, Stamataki D, Garcia-Perez L, Azzi C, Carr HL, Pokhilko A, Yu L, Howell S, Skehel M, Oxley D et al (2024) Protein degradation shapes developmental tempo in mouse and human neural progenitors. Preprint at https://www.biorxiv.org/content/10.1101/2024.08.01.604391v110.1016/j.devcel.2026.07.01442594861

[CR55] Owusu-Ansah E, Song W, Perrimon N (2013) Muscle mitohormesis promotes longevity via systemic repression of insulin signaling. Cell 155:699–712 24243023 10.1016/j.cell.2013.09.021 PMC3856681

[CR56] Pang Z, Lu Y, Zhou G, Hui F, Xu L, Viau C, Spigelman AF, MacDonald PE, Wishart DS, Li S et al (2024) MetaboAnalyst 6.0: towards a unified platform for metabolomics data processing, analysis and interpretation. Nucleic Acids Res 52:W398–W406 38587201 10.1093/nar/gkae253 PMC11223798

[CR57] Porte A de la, Schröder J, Thomas M, Geuder J, Sterr M, Pastor X, Sanderson LE, Barakat TS, Enard W, Marr C et al (2024) Single-cell multiome uncovers differences in glycogen metabolism underlying species-specific speed of development. Preprint at https://www.biorxiv.org/content/10.1101/2024.09.03.610938v3

[CR58] Rayon T, Stamataki D, Perez-Carrasco R, Garcia-Perez L, Barrington C, Melchionda M, Exelby K, Lazaro J, Tybulewicz VLJ, Fisher EMC et al (2020) Species-specific pace of development is associated with differences in protein stability. Science 369:eaba7667 32943498 10.1126/science.aba7667 PMC7116327

[CR59] Ready DF, Hanson TE, Benzer S (1976) Development of the Drosophila retina, a neurocrystalline lattice. Dev Biol 53:217–240 825400 10.1016/0012-1606(76)90225-6

[CR60] Rebelo AR, Homem CCF (2023) dMyc-dependent upregulation of CD98 amino acid transporters is required for Drosophila brain tumor growth. Cell Mol Life Sci 80:30 36609617 10.1007/s00018-022-04668-6 PMC9823048

[CR61] Roignant J-Y, Treisman JE (2009) Pattern formation in the Drosophila eye disc. Int J Dev Biol 53:795–804 19557685 10.1387/ijdb.072483jr PMC2713679

[CR62] Romani M, Sorrentino V, Oh C-M, Li H, de Lima TI, Zhang H, Shong M, Auwerx J (2021) NAD+ boosting reduces age-associated amyloidosis and restores mitochondrial homeostasis in muscle. Cell Rep 34:108660 33472069 10.1016/j.celrep.2020.108660 PMC7816122

[CR63] Sanz A, Soikkeli M, Portero-Otín M, Wilson A, Kemppainen E, McIlroy G, Ellilä S, Kemppainen KK, Tuomela T, Lakanmaa M et al (2010) Expression of the yeast NADH dehydrogenase Ndi1 in Drosophila confers increased lifespan independently of dietary restriction. Proc Natl Acad Sci USA 107:9105–9110 20435911 10.1073/pnas.0911539107 PMC2889079

[CR64] Satija R, Farrell JA, Gennert D, Schier AF, Regev A (2015) Spatial reconstruction of single-cell gene expression data. Nat Biotechnol 33:495–502 25867923 10.1038/nbt.3192 PMC4430369

[CR65] Seo BB, Kitajima-Ihara T, Chan EK, Scheffler IE, Matsuno-Yagi A, Yagi T (1998) Molecular remedy of complex I defects: rotenone-insensitive internal NADH-quinone oxidoreductase of *Saccharomyces cerevisiae* mitochondria restores the NADH oxidase activity of complex I-deficient mammalian cells. Proc Natl Acad Sci USA 95:9167–9171 9689052 10.1073/pnas.95.16.9167 PMC21310

[CR66] Sercel AJ, Sturm G, Gallagher D, St-Onge M-P, Kempes CP, Pontzer H, Hirano M, Picard M (2024) Hypermetabolism and energetic constraints in mitochondrial disorders. Nat Metab 6:192–195 38337097 10.1038/s42255-023-00968-8 PMC12066245

[CR67] Sharma R, Yang Y, Sharma A, Awasthi S, Awasthi YC (2004) Antioxidant role of glutathione S-transferases: protection against oxidant toxicity and regulation of stress-mediated apoptosis. Antioxid Redox Signal 6:289–300 15025930 10.1089/152308604322899350

[CR68] Spratford CM, Kumar JP (2015) Inhibition of daughterless by extramacrochaetae mediates notch-induced cell proliferation. Development 142:2058–2068 25977368 10.1242/dev.121855 PMC4460741

[CR69] Sturm G, Karan KR, Monzel AS, Santhanam B, Taivassalo T, Bris C, Ware SA, Cross M, Towheed A, Higgins-Chen A et al (2023) OxPhos defects cause hypermetabolism and reduce lifespan in cells and in patients with mitochondrial diseases. Commun Biol 6:1–22 36635485 10.1038/s42003-022-04303-x PMC9837150

[CR70] Sullivan GM, Feinn R (2012) Using effect size—or why the P value is not enough. J Grad Med Educ 4:279–282 23997866 10.4300/JGME-D-12-00156.1 PMC3444174

[CR71] Sullivan LB, Gui DY, Hosios AM, Bush LN, Freinkman E, Vander Heiden MG (2015) Supporting aspartate biosynthesis is an essential function of respiration in proliferating cells. Cell 162:552–563 26232225 10.1016/j.cell.2015.07.017 PMC4522278

[CR72] Sykiotis GP, Bohmann D (2008) Keap1/Nrf2 signaling regulates oxidative stress tolerance and lifespan in Drosophila. Dev Cell 14:76–85 18194654 10.1016/j.devcel.2007.12.002 PMC2257869

[CR73] Titov DV, Cracan V, Goodman RP, Peng J, Grabarek Z, Mootha VK (2016) Complementation of mitochondrial electron transport chain by manipulation of the NAD+/NADH ratio. Science 352:231–235 27124460 10.1126/science.aad4017 PMC4850741

[CR74] Toshniwal AG, Lam G, Bott AJ, Cluntun AA, Skabelund R, Nam H-J, Wisidagama DR, Thummel CS, Rutter J (2025) The fate of pyruvate dictates cell growth by modulating cellular redox potential. eLife 13:RP103705 41400249 10.7554/eLife.103705 PMC12707817

[CR75] Trcek T, Lionnet T, Shroff H, Lehmann R (2017) mRNA quantification using single-molecule FISH in Drosophila embryos. Nat Protoc 12:1326–1348 28594816 10.1038/nprot.2017.030 PMC6668020

[CR76] Tsanov N, Samacoits A, Chouaib R, Traboulsi A-M, Gostan T, Weber C, Zimmer C, Zibara K, Walter T, Peter M et al (2016) smiFISH and FISH-quant—a flexible single RNA detection approach with super-resolution capability. Nucleic Acids Res 44:e165 27599845 10.1093/nar/gkw784 PMC5159540

[CR77] Tsao C-K, Ku H-Y, Lee Y-M, Huang Y-F, Sun YH (2016) Long term ex vivo culture and live imaging of Drosophila larval imaginal discs. PLoS ONE 11:e0163744 27685172 10.1371/journal.pone.0163744 PMC5042436

[CR78] Wartlick O, Jülicher F, Gonzalez-Gaitan M (2014) Growth control by a moving morphogen gradient during Drosophila eye development. Development 141:1884–1893 24757005 10.1242/dev.105650

[CR79] Wirth C, Brandt U, Hunte C, Zickermann V (2016) Structure and function of mitochondrial complex I. Biochim et Biophys Acta (BBA) - Bioenerg 1857:902–914 10.1016/j.bbabio.2016.02.013 26921811

[CR80] Xia D, Yu CA, Kim H, Xia JZ, Kachurin AM, Zhang L, Yu L, Deisenhofer J (1997) Crystal structure of the cytochrome bc1 complex from bovine heart mitochondria. Science 277:60–66 9204897 10.1126/science.277.5322.60 PMC12235523

[CR81] Xie Z, Bailey A, Kuleshov MV, Clarke DJB, Evangelista JE, Jenkins SL, Lachmann A, Wojciechowicz ML, Kropiwnicki E, Jagodnik KM et al (2021) Gene set knowledge discovery with enrichr. Curr Protoc 1:e90 33780170 10.1002/cpz1.90 PMC8152575

[CR82] Yeung K, Bollepogu Raja KK, Shim Y-K, Li Y, Chen R, Mardon G (2022) Single cell RNA sequencing of the adult Drosophila eye reveals distinct clusters and novel marker genes for all major cell types. Commun Biol 5:1–18 36517671 10.1038/s42003-022-04337-1 PMC9751288

[CR83] Yu S, Jang Y, Paik D, Lee E, Park J-J (2015) Nmdmc overexpression extends Drosophila lifespan and reduces levels of mitochondrial reactive oxygen species. Biochem Biophys Res Commun 465:845–850 26319556 10.1016/j.bbrc.2015.08.098

[CR84] Zhang Q, Li Z, Li Q, Trammell SA, Schmidt MS, Pires KM, Cai J, Zhang Y, Kenny H, Boudina S et al (2024) Control of NAD+ homeostasis by autophagic flux modulates mitochondrial and cardiac function. EMBO J 43:362–390 38212381 10.1038/s44318-023-00009-w PMC10897141

[CR85] Zhao Y, Hu Q, Cheng F, Su N, Wang A, Zou Y, Hu H, Chen X, Zhou H-M, Huang X et al (2015) SoNar, a highly responsive NAD+/NADH sensor, allows high-throughput metabolic screening of anti-tumor agents. Cell Metab 21:777–789 25955212 10.1016/j.cmet.2015.04.009 PMC4427571

