# Supplementary figures and images for: NAD+ supply and redox state limit developmental speed in the Drosophila eye
Source: EMBO J. 2026 May 13;45(12):4094–123. doi: 10.1038/s44318-026-00801-4 (PMC13270060; doi:10.1038/s44318-026-00801-4)

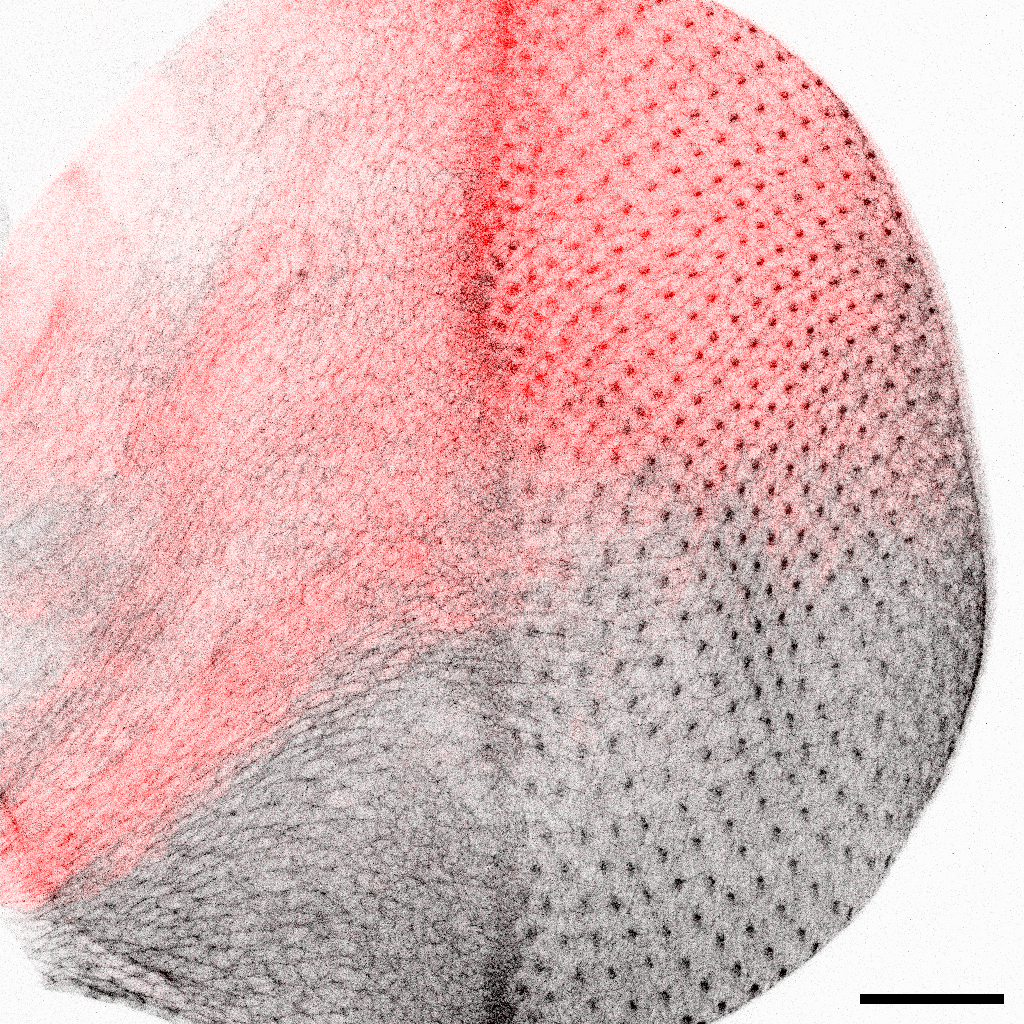

Supplement: Supplementary file 6 — Source data Fig. 1 [file 44318_2026_801_MOESM6_ESM.zip › Fig1/C/240104_14 MAXz1-10_flipped_vertically (RGB).tif]

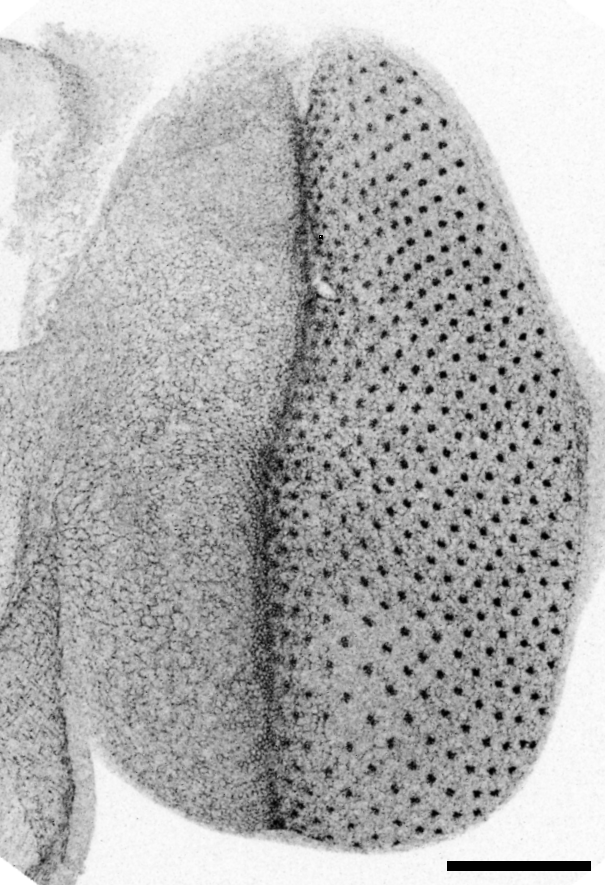

Supplement: Supplementary file 6 — Source data Fig. 1 [file 44318_2026_801_MOESM6_ESM.zip › Fig1/D/210705_yuting_cropped_MAX_3-20_flipped90vertical_rotate-40_Image 22.tif (RGB).tif]

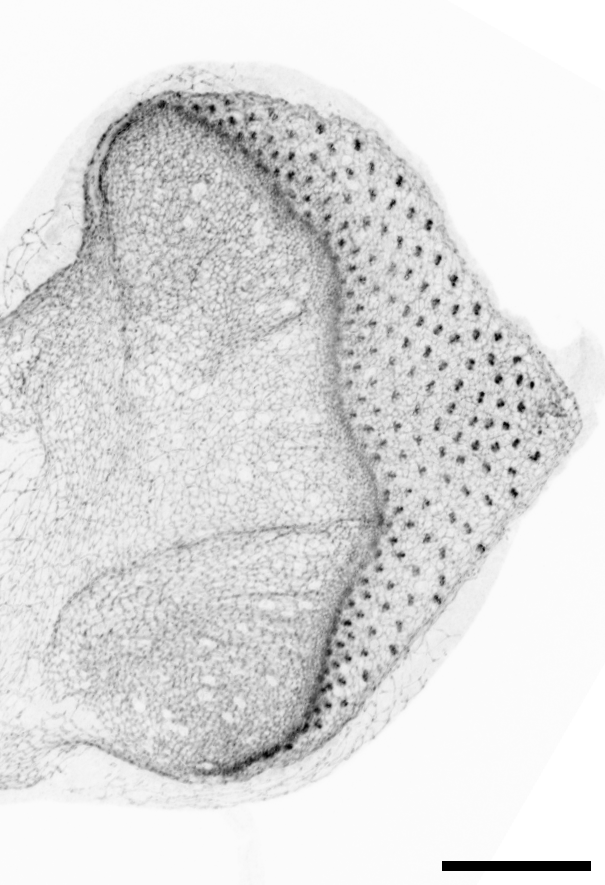

Supplement: Supplementary file 6 — Source data Fig. 1 [file 44318_2026_801_MOESM6_ESM.zip › Fig1/E/250226_MAX_z11-24_rotate110_pten_001.tif]

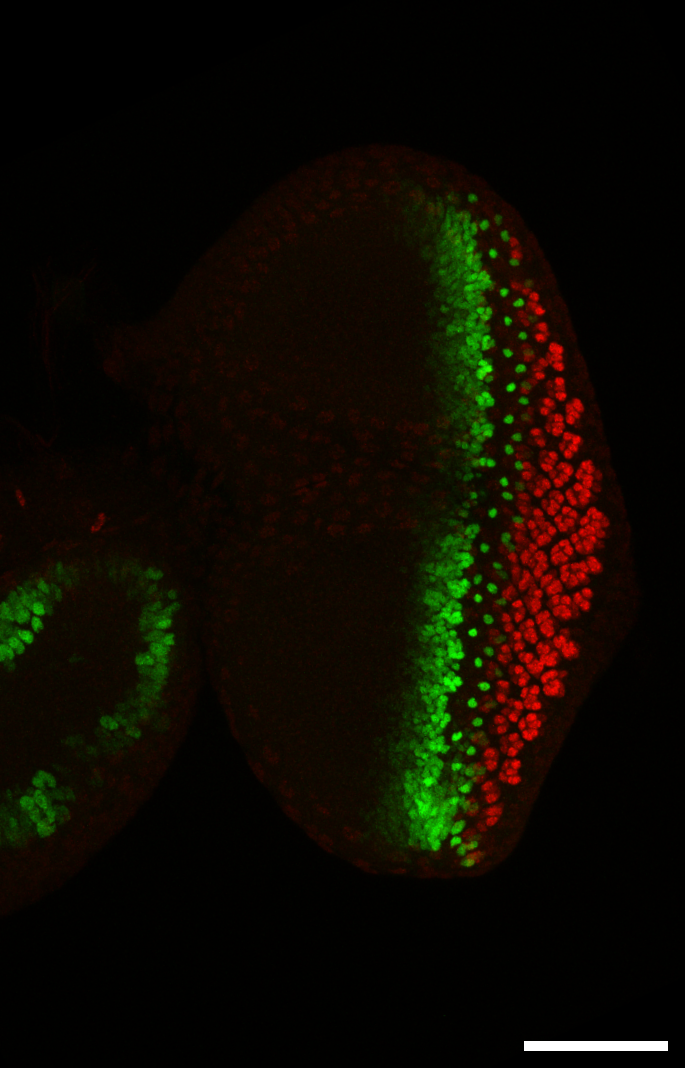

Supplement: Supplementary file 6 — Source data Fig. 1 [file 44318_2026_801_MOESM6_ESM.zip › Fig1/B/201022_3 Yuting Maxz3-25.tif (RGB).tif]

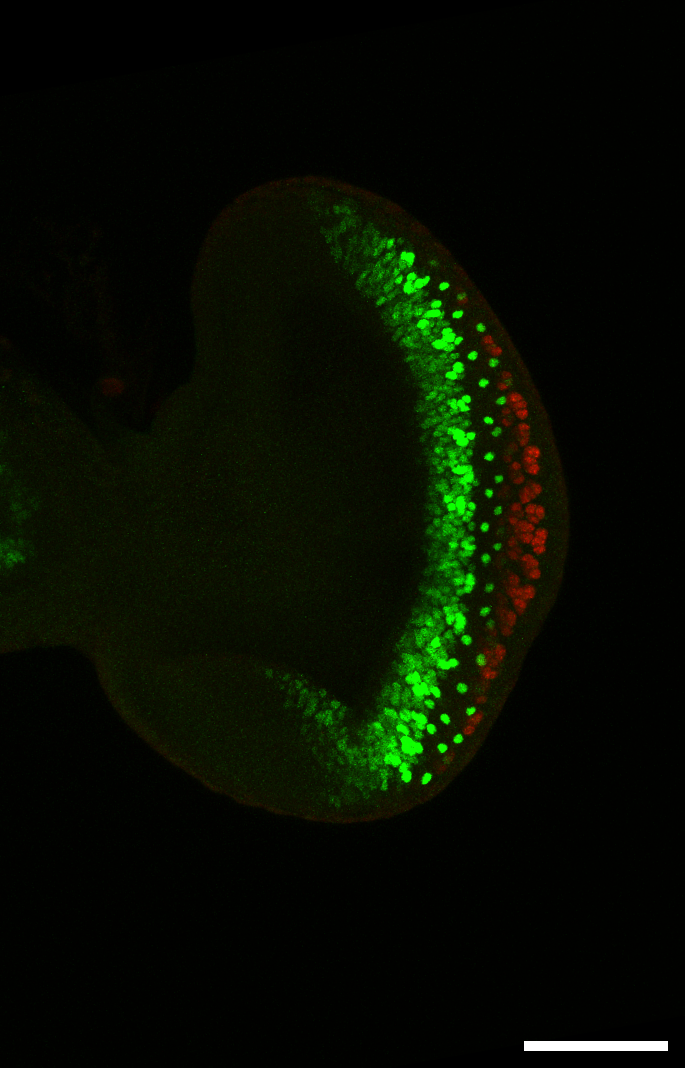

Supplement: Supplementary file 6 — Source data Fig. 1 [file 44318_2026_801_MOESM6_ESM.zip › Fig1/B/201022_6 Yuting MAXz14-33.tif (RGB).tif]

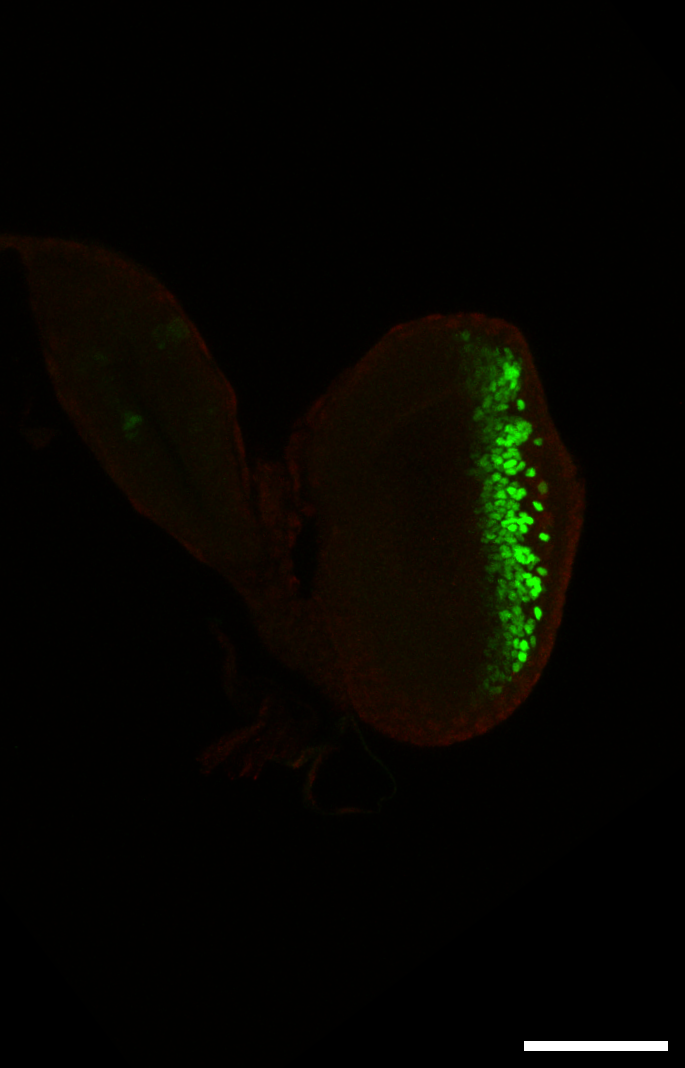

Supplement: Supplementary file 6 — Source data Fig. 1 [file 44318_2026_801_MOESM6_ESM.zip › Fig1/B/201022_7 Yuting MAX19_28.tif (RGB).tif]

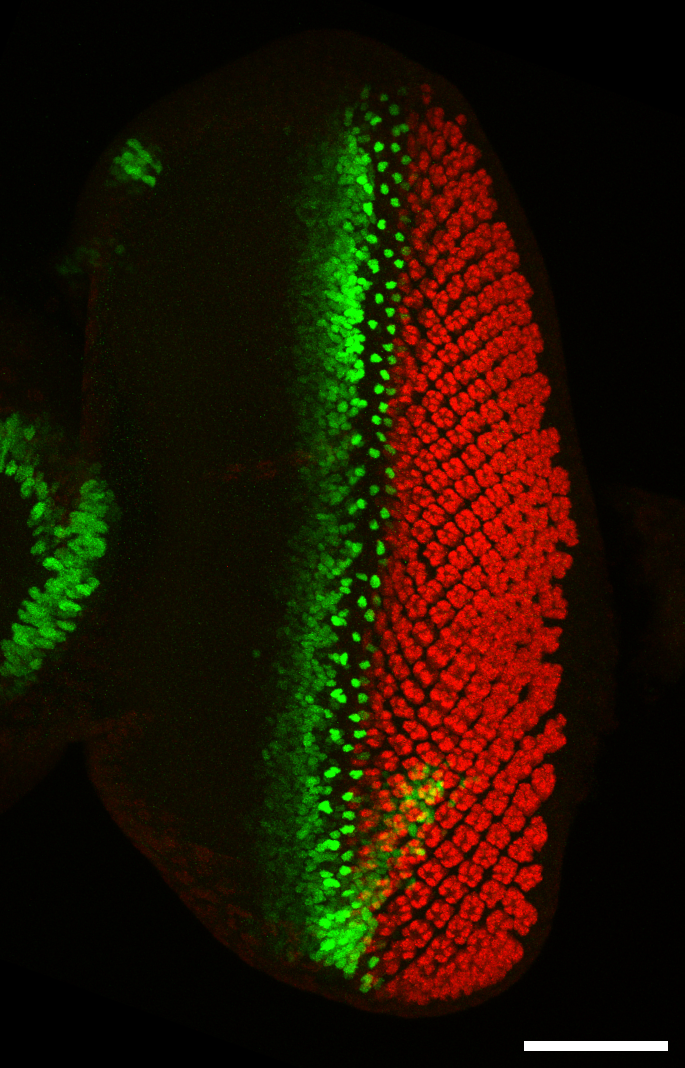

Supplement: Supplementary file 6 — Source data Fig. 1 [file 44318_2026_801_MOESM6_ESM.zip › Fig1/B/201022_2 Yuting MAXz3-31.tif (RGB).tif]

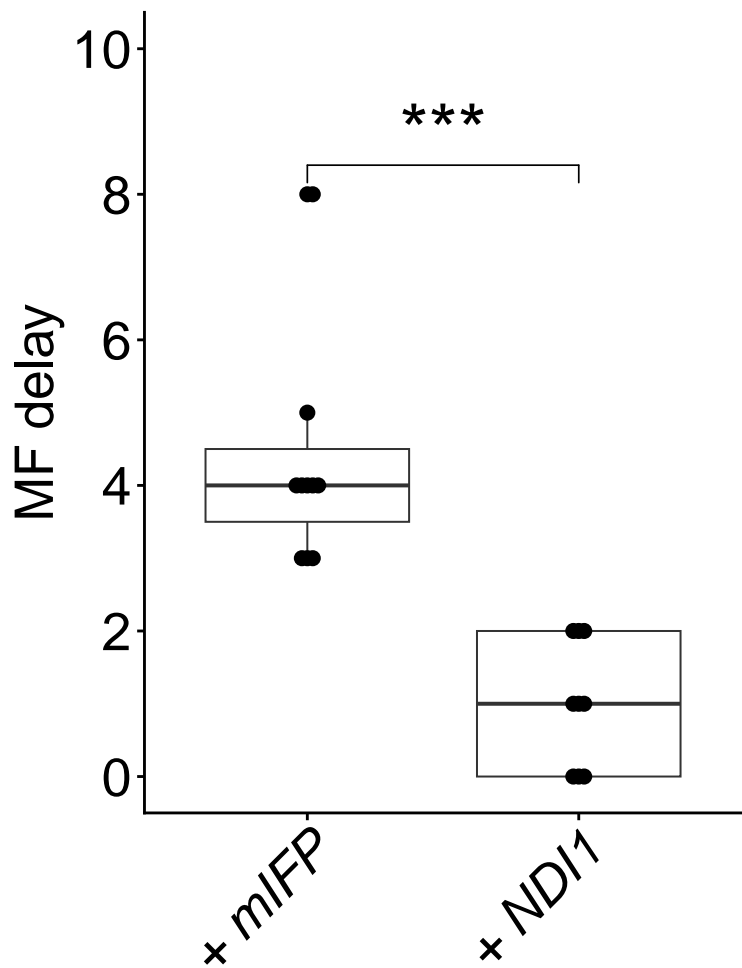

Supplement: Supplementary file 7 — Source data Fig. 2 [file 44318_2026_801_MOESM7_ESM.zip › Fig2/D/NDi1_revision.pdf]

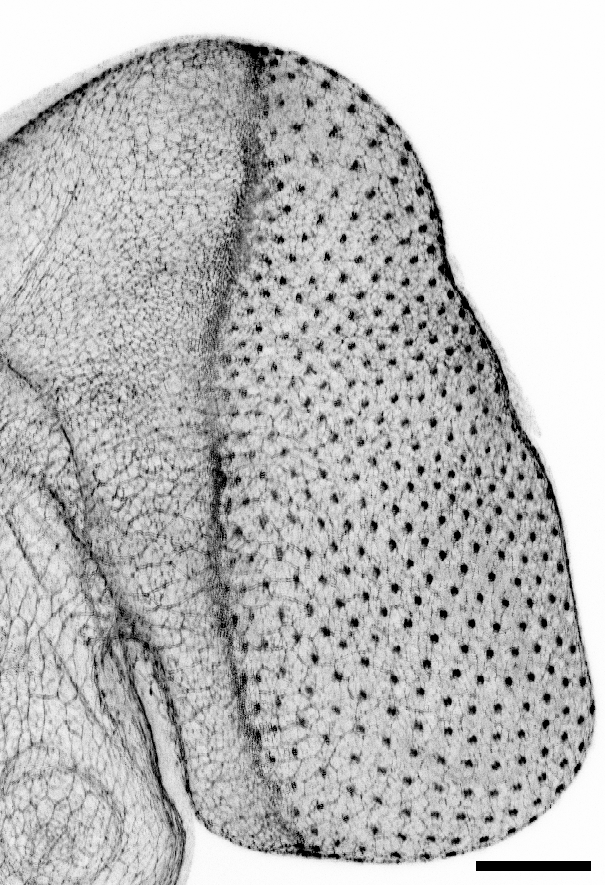

Supplement: Supplementary file 7 — Source data Fig. 2 [file 44318_2026_801_MOESM7_ESM.zip › Fig2/B/251022_ND42_002_rotate90_flip horizontal-MAX9-17_IFP_ND-42_002-2.tif]

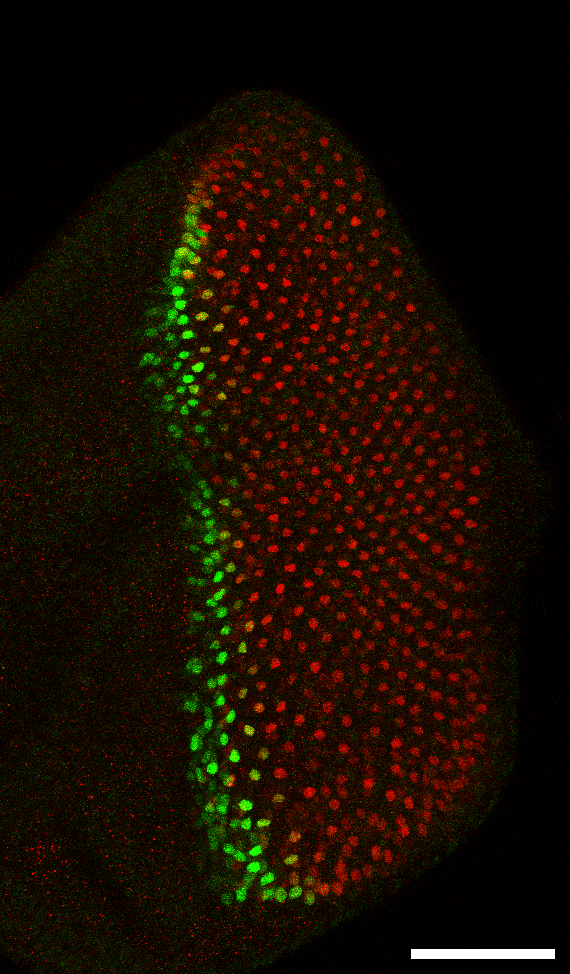

Supplement: Supplementary file 8 — Source data Fig. 3 [file 44318_2026_801_MOESM8_ESM.zip › Fig3/B/Yuting_210922_Image36_MAX1-49_fliphorizontal.tif]

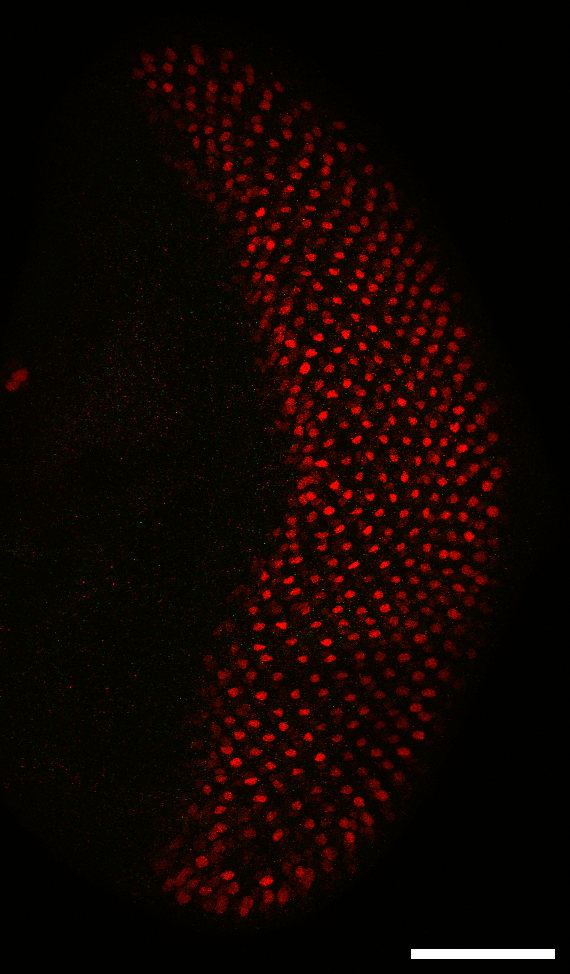

Supplement: Supplementary file 8 — Source data Fig. 3 [file 44318_2026_801_MOESM8_ESM.zip › Fig3/B/Yuting_210616_MAX1-37_Image 2(RGB).tif]

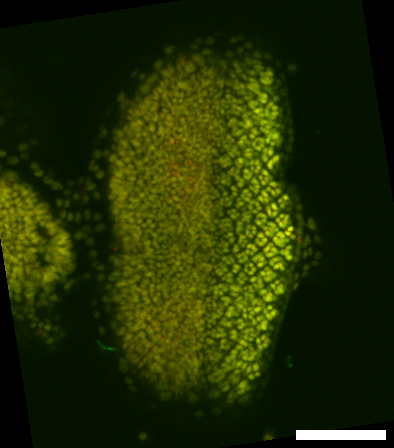

Supplement: Supplementary file 9 — Source data Fig. 4 [file 44318_2026_801_MOESM9_ESM.zip › Fig4/G/230711_w_AVG_Image10_slice7-17_rotated90_cropped_roi0001-0264-0295 (RGB).tif]

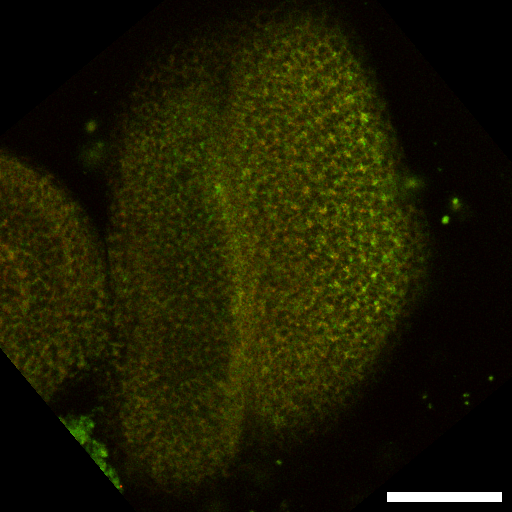

Supplement: Supplementary file 9 — Source data Fig. 4 [file 44318_2026_801_MOESM9_ESM.zip › Fig4/A/250320_AVG_wt_011_z12-22.nd2 (RGB).tif]

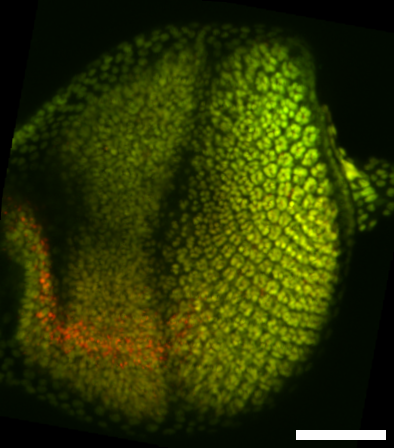

Supplement: Supplementary file 9 — Source data Fig. 4 [file 44318_2026_801_MOESM9_ESM.zip › Fig4/H/230713_ND42_AVG_Image20_slice9-19_rotated90_flippedhorizental_cropped_roi0001-0264-0295 (RGB).tif]

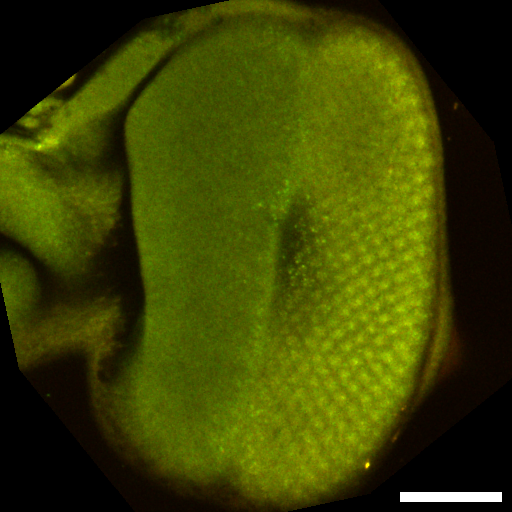

Supplement: Supplementary file 9 — Source data Fig. 4 [file 44318_2026_801_MOESM9_ESM.zip › Fig4/D/NV_250324_AVG_wt_001_cyto_z17-27.nd2 (RGB).tif]

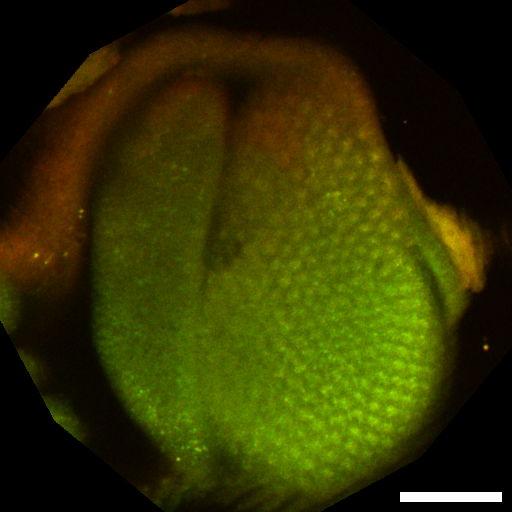

Supplement: Supplementary file 9 — Source data Fig. 4 [file 44318_2026_801_MOESM9_ESM.zip › Fig4/E/NV+250320_AVG_ND42_002-1_z27-37.nd2 (RGB).tif]

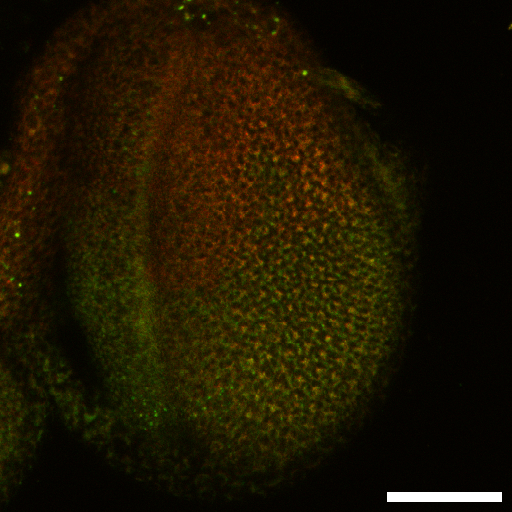

Supplement: Supplementary file 9 — Source data Fig. 4 [file 44318_2026_801_MOESM9_ESM.zip › Fig4/B/250320_AVG_ND-42_004_z34-44.nd2 (RGB).tif]

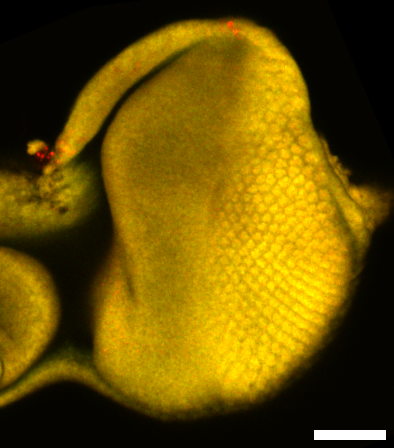

Supplement: Supplementary file 9 — Source data Fig. 4 [file 44318_2026_801_MOESM9_ESM.zip › Fig4/N/N/230703_AVG_Image1_slice23-33_rotated90_flippedhorizontal_cropped.tif (RGB).tif]

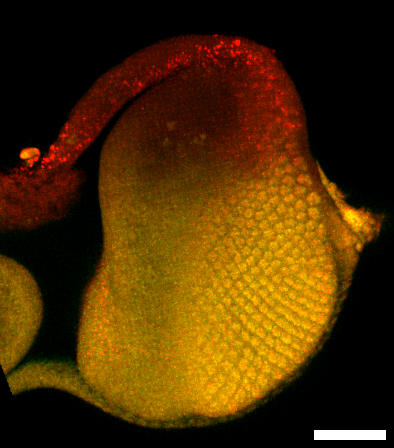

Supplement: Supplementary file 9 — Source data Fig. 4 [file 44318_2026_801_MOESM9_ESM.zip › Fig4/N/N'/230703_AVG_Image46_slice22-32_rotated90_flippedhorizontal_cropped_roi0001-0266-0210.tif (RGB)-1.tif]

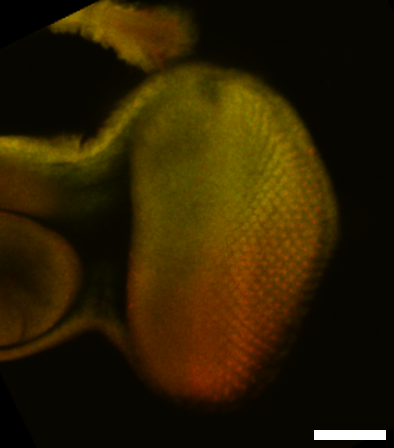

Supplement: Supplementary file 9 — Source data Fig. 4 [file 44318_2026_801_MOESM9_ESM.zip › Fig4/L/L'/230629_AVG_Image107_slice64-74_rotated47.8_cropped.tif (RGB)-1.tif]

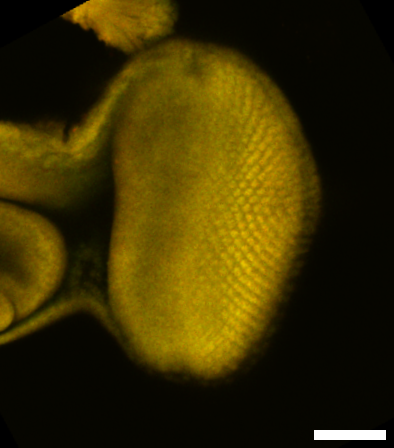

Supplement: Supplementary file 9 — Source data Fig. 4 [file 44318_2026_801_MOESM9_ESM.zip › Fig4/L/L/230629_AVG_Image38_slice60-70_rotated47.8_cropped.tif (RGB).tif]

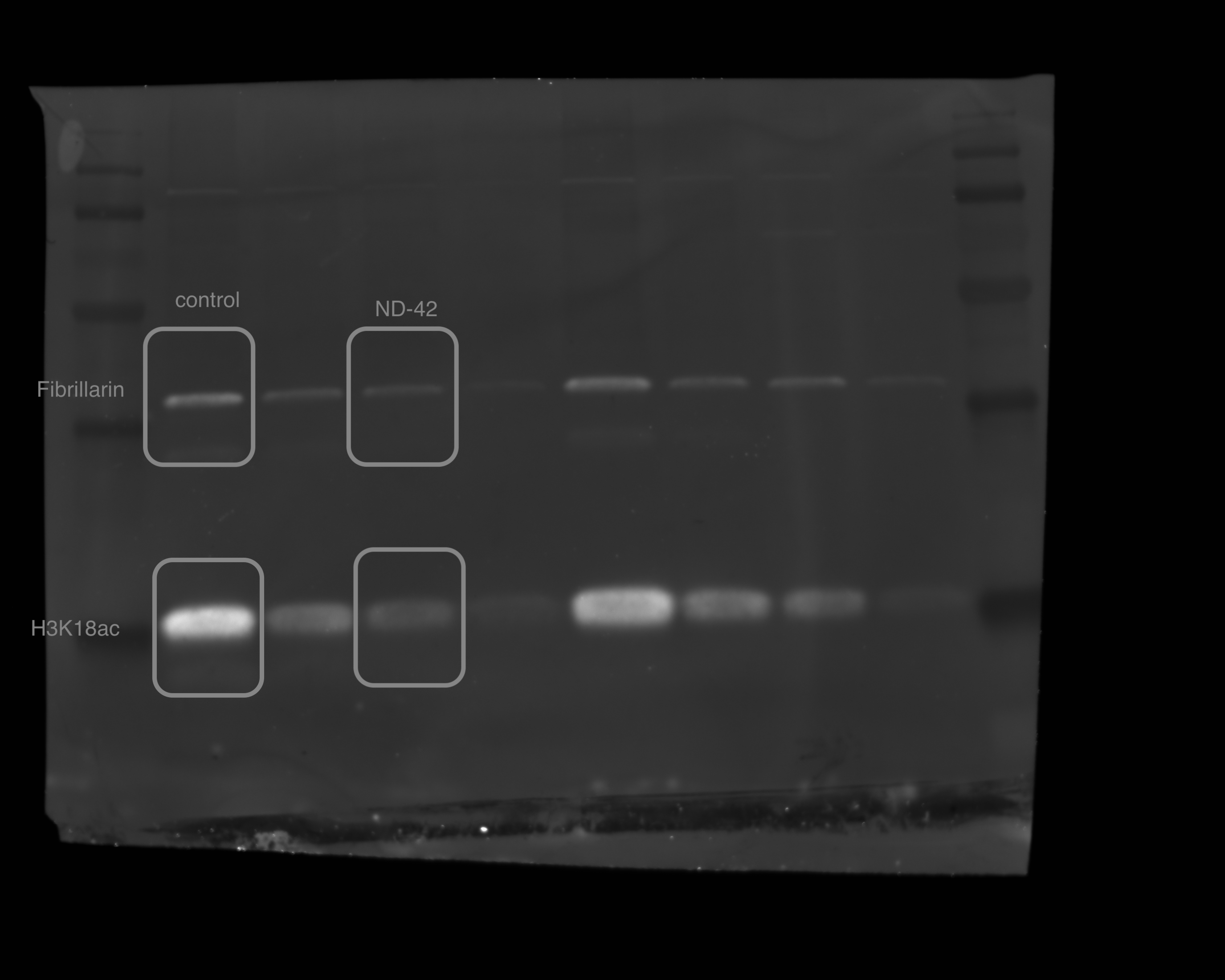

Supplement: Supplementary file 10 — Source data Fig. 5 [file 44318_2026_801_MOESM10_ESM.zip › Fig5/N/khallil 2025-12-12 12h07m21srevision.tiff]

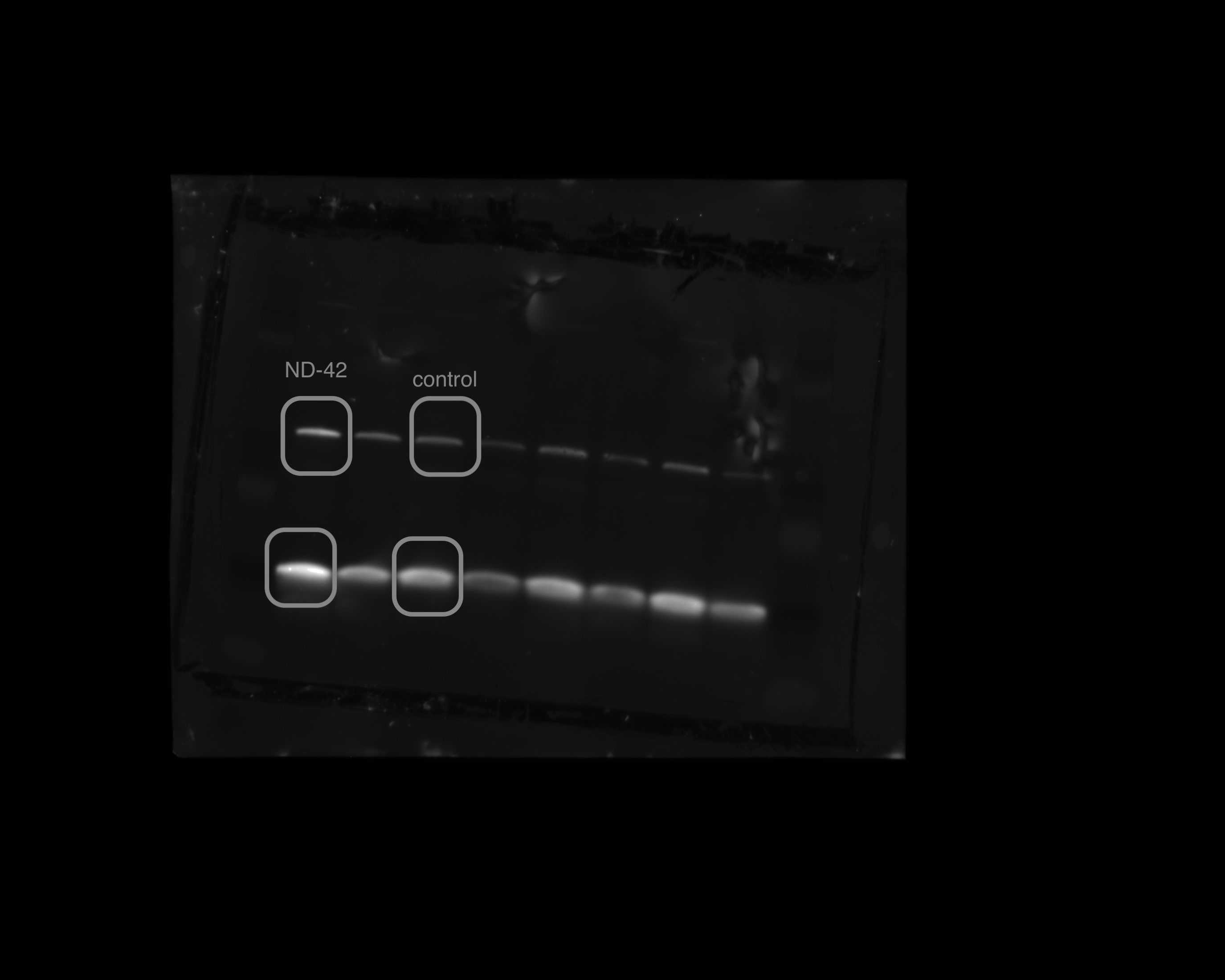

Supplement: Supplementary file 10 — Source data Fig. 5 [file 44318_2026_801_MOESM10_ESM.zip › Fig5/N/13h24m09s(StarBright B700)_revision.tiff]

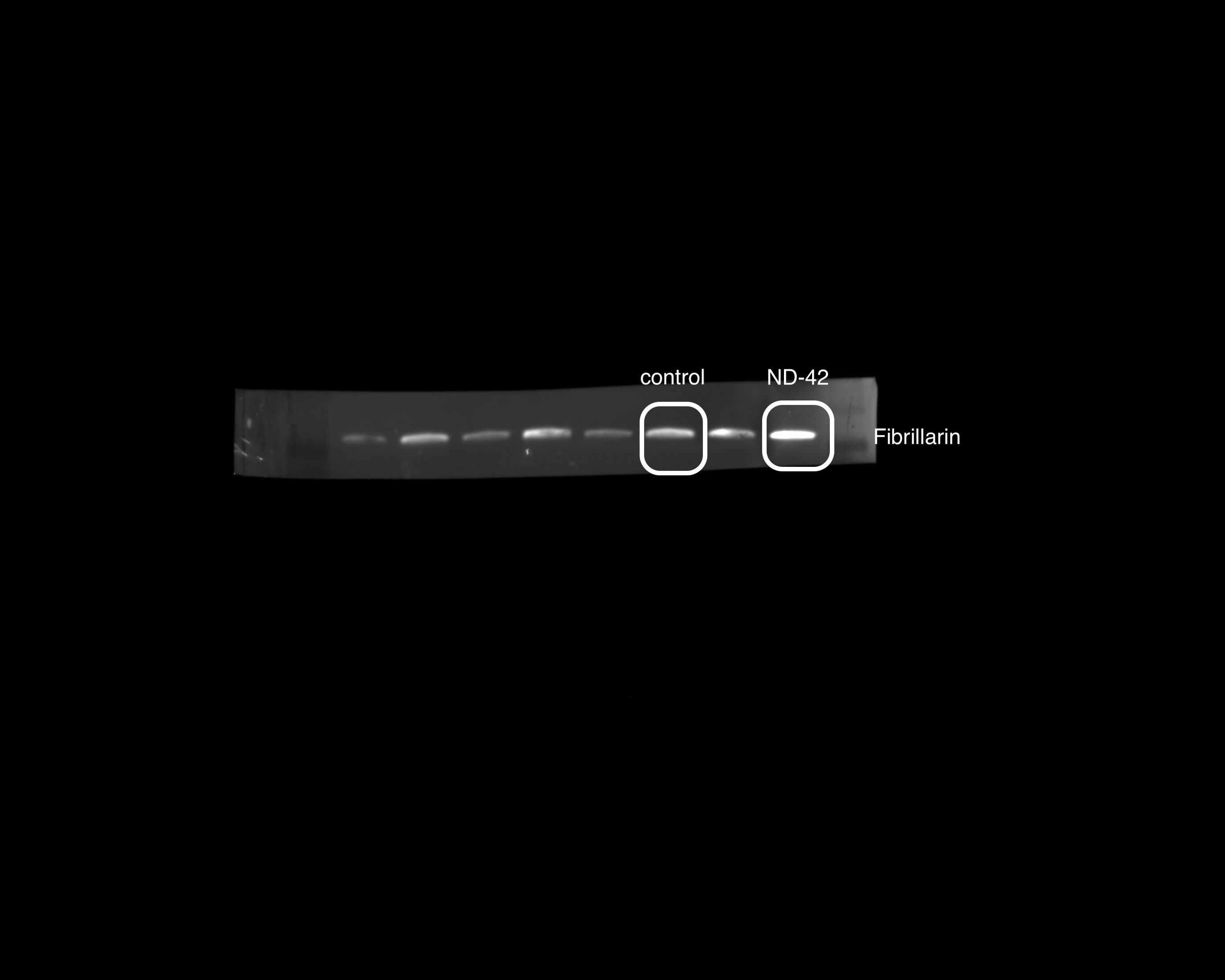

Supplement: Supplementary file 10 — Source data Fig. 5 [file 44318_2026_801_MOESM10_ESM.zip › Fig5/M/13h18m08s(StarBright B700)._revision.tiff]

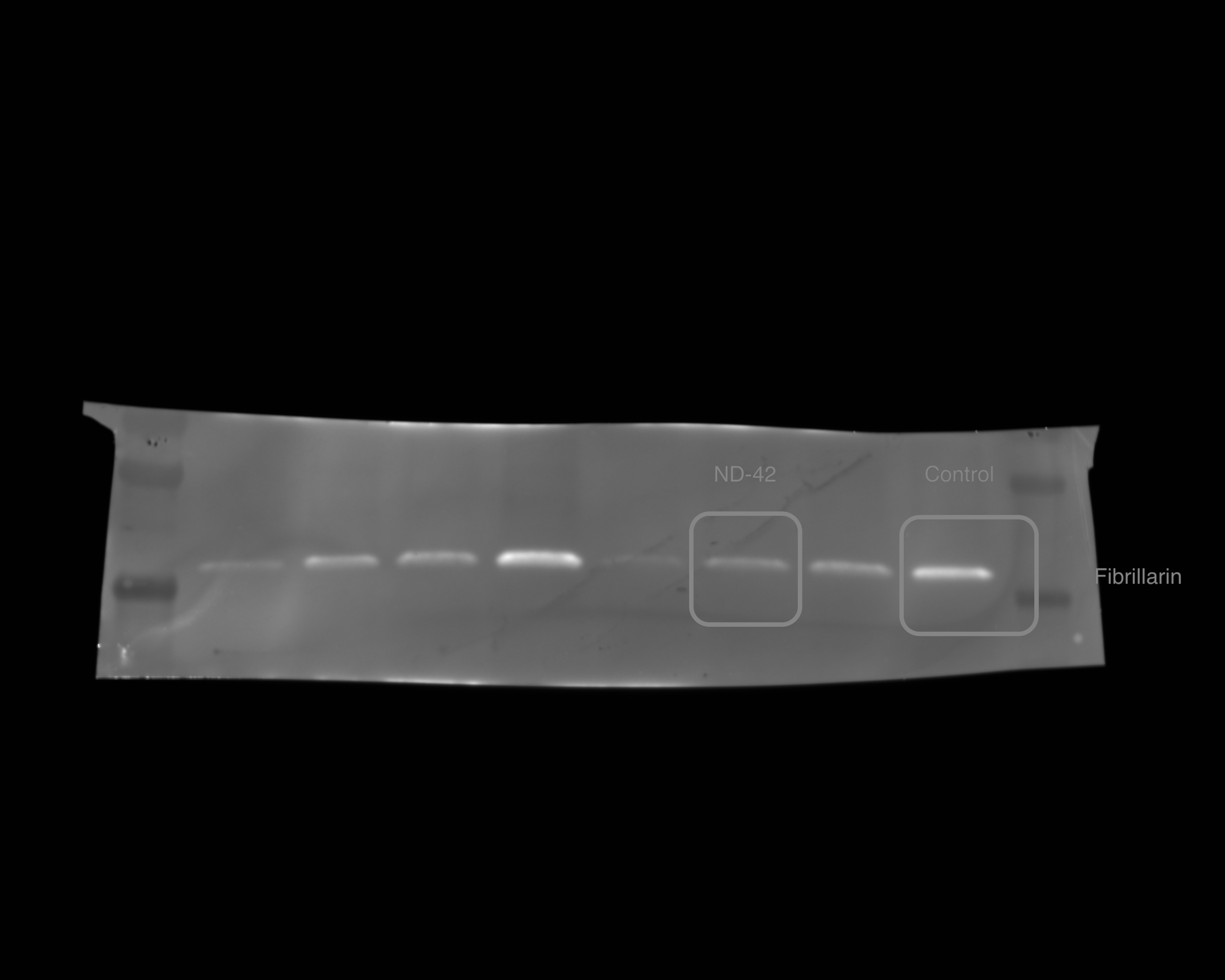

Supplement: Supplementary file 10 — Source data Fig. 5 [file 44318_2026_801_MOESM10_ESM.zip › Fig5/M/khallil 2025-12-12 12h09m52s(StarBright B700).raw16.tif]

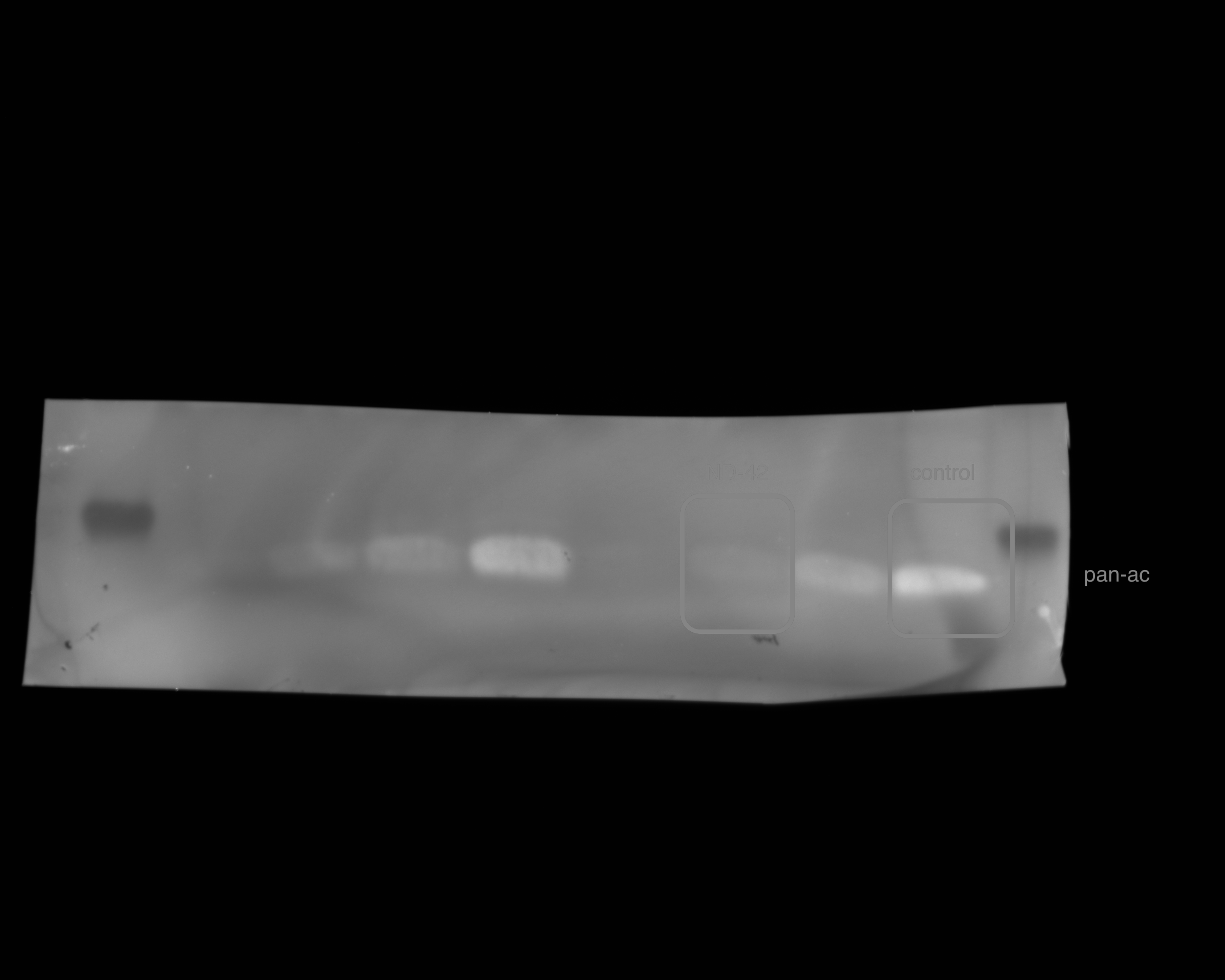

Supplement: Supplementary file 10 — Source data Fig. 5 [file 44318_2026_801_MOESM10_ESM.zip › Fig5/M/flipped vertically_khallil 2025-12-12 12h13m03s(StarBright B700)_revision.raw16.tiff]

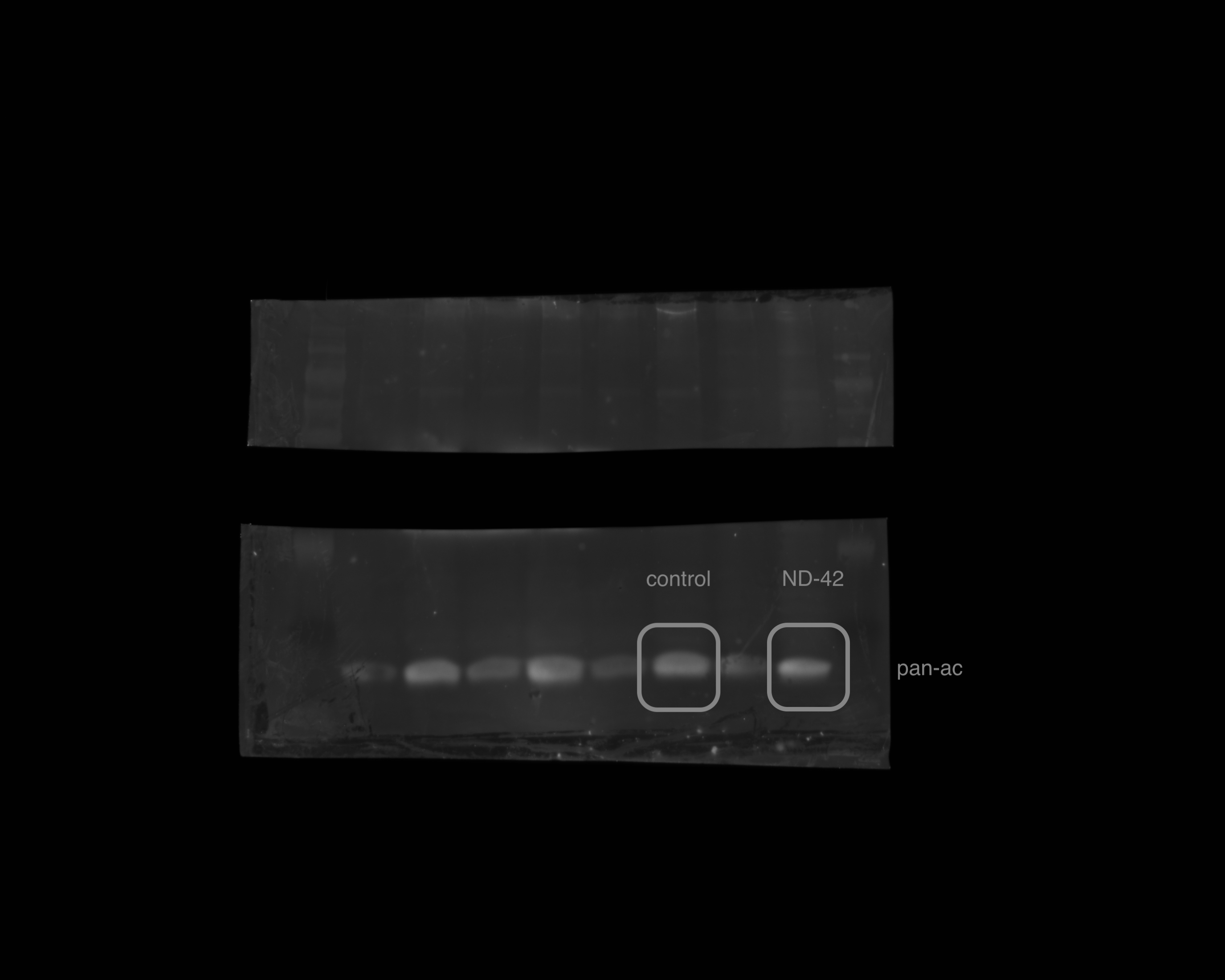

Supplement: Supplementary file 10 — Source data Fig. 5 [file 44318_2026_801_MOESM10_ESM.zip › Fig5/M/ 13h21m24s(StarBright B700)revision.tiff]

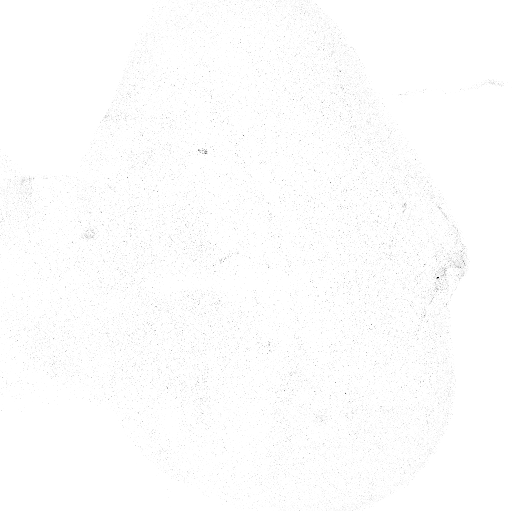

Supplement: Supplementary file 10 — Source data Fig. 5 [file 44318_2026_801_MOESM10_ESM.zip › Fig5/I/I''/240320_8 MAXz11-34 wt path oriented.tif]

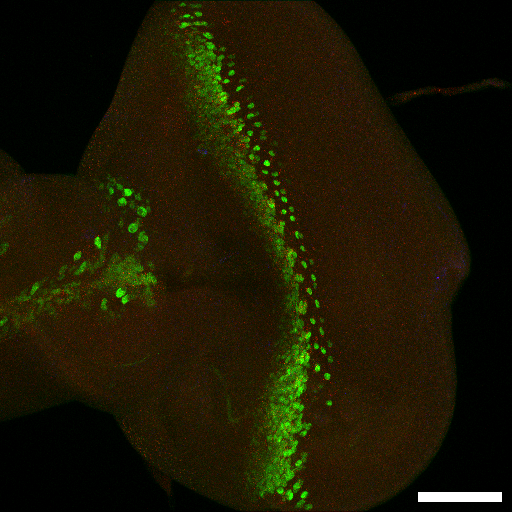

Supplement: Supplementary file 10 — Source data Fig. 5 [file 44318_2026_801_MOESM10_ESM.zip › Fig5/I/I/240320_8 MAXz11-34 wt merged(RGB).tif]

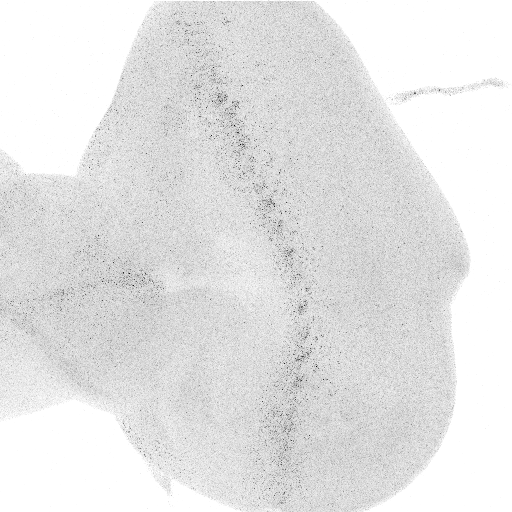

Supplement: Supplementary file 10 — Source data Fig. 5 [file 44318_2026_801_MOESM10_ESM.zip › Fig5/I/I'/240320_8 MAXz11-34ldh_oriented.tif (RGB).tif]

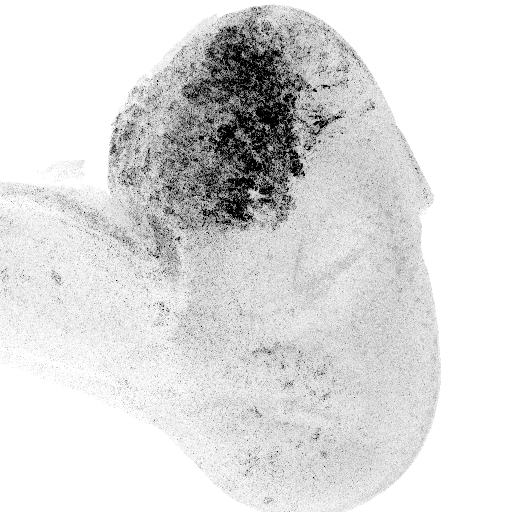

Supplement: Supplementary file 10 — Source data Fig. 5 [file 44318_2026_801_MOESM10_ESM.zip › Fig5/J/J''/240320_21 MAXz4-16 ND-42RNAi path_oriented.tif]

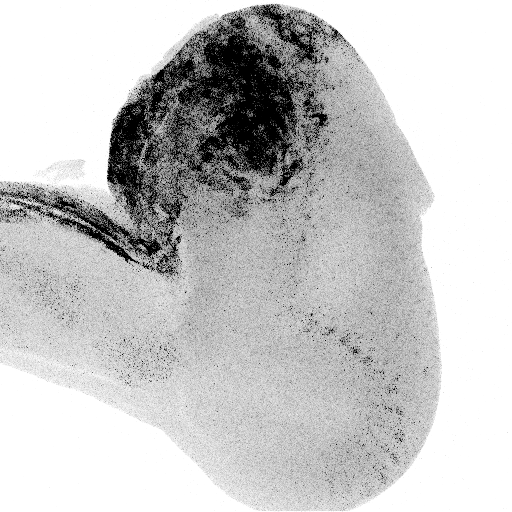

Supplement: Supplementary file 10 — Source data Fig. 5 [file 44318_2026_801_MOESM10_ESM.zip › Fig5/J/J'/240320_21 MAXz4-16 ND-42RNAi Ldh_oriented.tif]

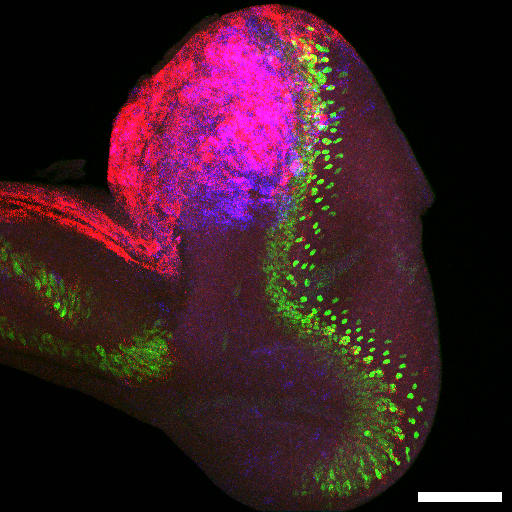

Supplement: Supplementary file 10 — Source data Fig. 5 [file 44318_2026_801_MOESM10_ESM.zip › Fig5/J/J/240320_21 MAXz4-16 ND-42RNAi merged(RGB).tif]

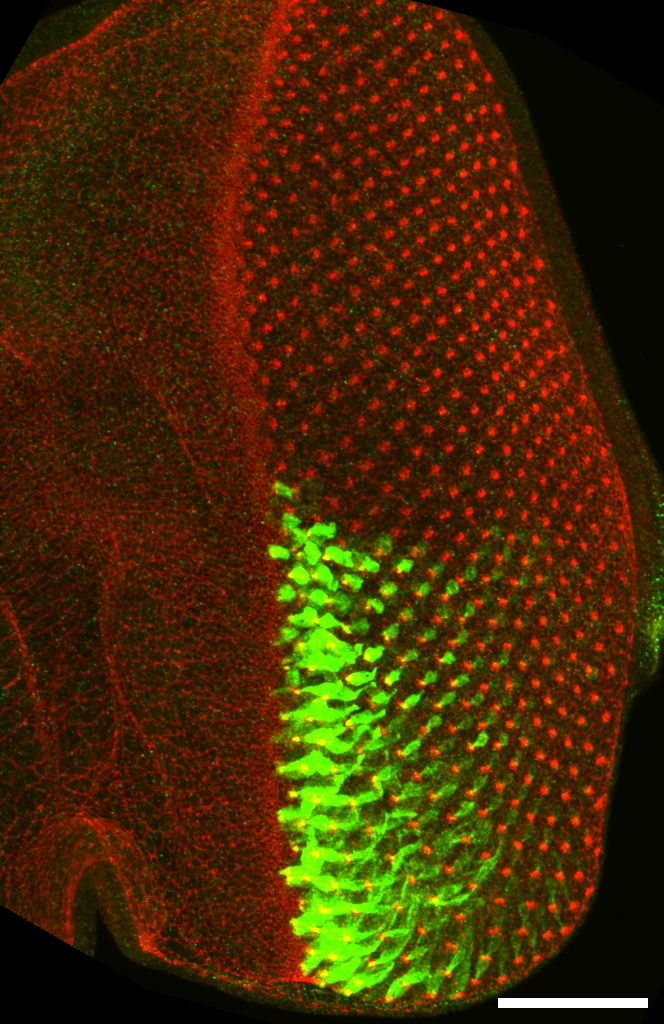

Supplement: Supplementary file 11 — Source data Fig. 6 [file 44318_2026_801_MOESM11_ESM.zip › Fig6/F/250513_010_MAX5-16_fliphorizental_32degree_Foxo_Ecad_GFP_-1.nd2 (RGB).tif]

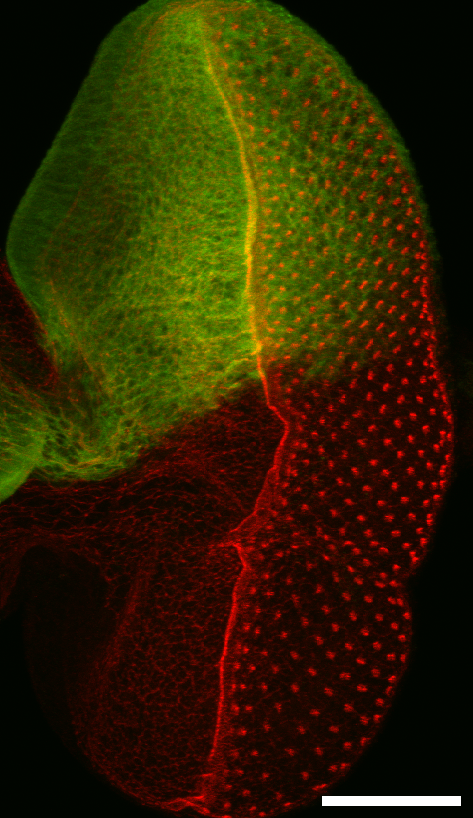

Supplement: Supplementary file 11 — Source data Fig. 6 [file 44318_2026_801_MOESM11_ESM.zip › Fig6/E/250319_007_MAX13-25_Foxo_GFP_.nd2 (RGB).tif]

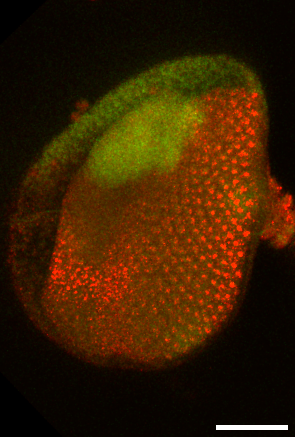

Supplement: Supplementary file 11 — Source data Fig. 6 [file 44318_2026_801_MOESM11_ESM.zip › Fig6/C/C'/240607_8_MAX_18-32_Image 21.czi - Image 21 #8-1 (RGB).tif]

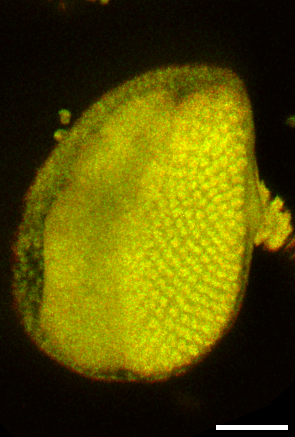

Supplement: Supplementary file 11 — Source data Fig. 6 [file 44318_2026_801_MOESM11_ESM.zip › Fig6/C/C/240607_8_MAX_beforedrug_ND42_rescue.czi - beforedrug_ND42_rescue #8-1 (RGB).tif]

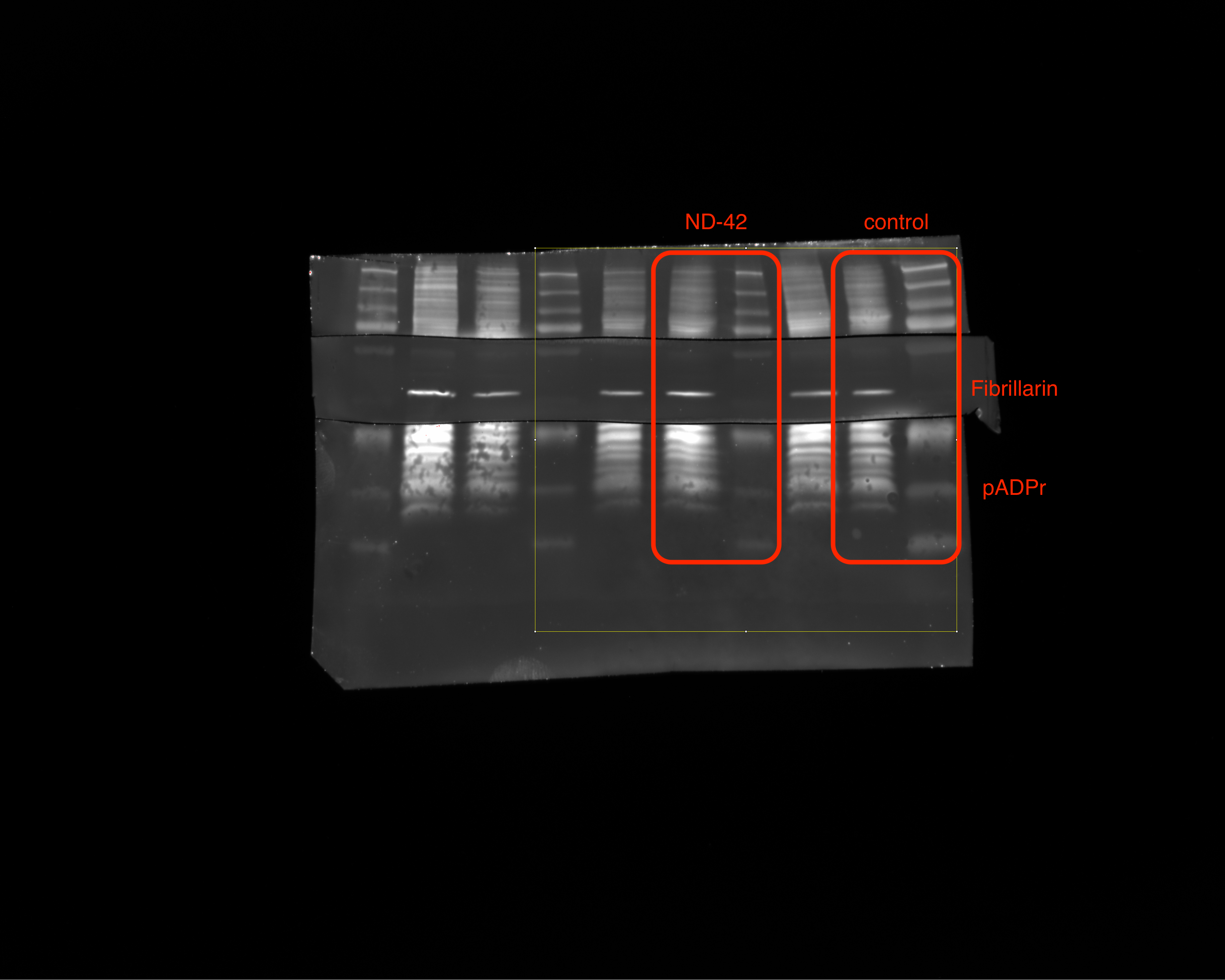

Supplement: Supplementary file 12 — Source data Fig. 7 [file 44318_2026_801_MOESM12_ESM.zip › Fig7/H/khallil 2025-04-08 16h04m01s revision.tiff]
