# Supplementary material for: NAD+ supply and redox state limit developmental speed in the Drosophila eye
Source: EMBO J. 2026 May 13;45(12):4094–123. doi: 10.1038/s44318-026-00801-4 (PMC13270060; doi:10.1038/s44318-026-00801-4)
Supplement: Supplementary file 13 — Expanded View Figures [file 44318_2026_801_MOESM13_ESM.pdf]

Expanded View Figures

Figure EV1. Patterning speed analysis, conditional silencing of the *ND-42* gene, and co-expression of the *ara*, *caup*, and *mirr* genes.

(**A**) The MF delay in *mirr>chico^{RNAi}* eye discs increased with developmental age, as determined here using the total number of rows measured in the *v* compartment (the regression line is shown; $R^2 = 0.5$). (**B**, **C**) Scanning Electron Microscopy (SEM) images of adult fly eyes. A regular crystal-like array of ommatidia was observed in control (**A**), whereas minor defects suggestive of interrupted rows were seen in the dorsal part of the eye in *mirr>chico^{RNAi}* flies (**B**; white circles indicate where non-consecutive rows appeared to merge; the star indicates a partial interruption; orange and blue dots provide a visualization tool to follow the regular rows of ommatidia). (**D**) The speed of progression of the MF was measured in control *mirr > mIFP* discs from larvae grown at 18 °C (orange) and 25 °C (blue) using a recombination-based RFP-to-GFP switch. The MF moved twice as slowly at 18 °C (0.23 rows/h, $n = 32$) than at 25 °C (0.51 rows/h, $n = 22$). Error bars show standard deviation. (**E–H**) Dachsund (Dac, red in (**E**, **F**)) and Elav (red, in (**G**, **H**)) showed similar patterns of expression in control *mirr-Gal4 ato^{GFP}* (**D**, **F**) and *mirr > ND-42^{RNAi} ato^{GFP}* eye discs (**E**, **G**). AtoGPP (green) marked the position of the differentiation front. Dac is a transcription cofactor expressed in a patterned manner along the AP axis, and Elav is an RNA-binding protein expressed in photoreceptors. Dac and Elav are used here as AP patterning markers. (**I**) The silencing of *ND-42* led to a decrease in proliferation (number of pH3-positive cells per surface area) in UPs and to a loss of the SMW (plotted as DCs, $n = 9$). (**J–L**) To conditionally silence the *ND-42* gene in all eye disc cells, we used a *tub-Gal4 tub-Gal80^{ts}* driver (*tub^{ts}*) and grew larvae at a restrictive temperature (29 °C). To monitor the downregulation of *ND-42* gene activity, we used *Ldh* mRNA accumulation as a reporter for reduced *ND-42* activity (see Fig. 5I–J″). We observed strong *Ldh* expression in UPs, peripodial, and antenna cells after 3 days (3 d) of conditional knockdown of *ND-42* (**K**). This effect appeared to be stronger than the one observed using *mirr-Gal4* (**J**). The *Ldh* signal was very weak after 2 days at 29 °C (**L**). The position of the MF was indicated by a white dotted line (disc outlines, gray dotted lines). (**M**, **N**) Expression pattern of the *ara* (red in (**N**, **N″**)), *caup* (red in (**M**, **M″**)) and *mirr* genes (green in (**M–N′**)) showing that all three genes were largely co-expressed in *d* eye field and peripodial cells (MF position indicated by white dotted line). The box plots show the median and the interquartile range from the 25th to 75th percentile; whiskers extend to data points within 1.5× the interquartile range of the lower and upper quartiles, representing the approximate minimum and maximum non-outlier values. Wilcoxon test, *P* values: **<0.01. Scale bars, 50 µm (**E–N″**) and 100 µm (**B**, **C**).

© The Author(s)

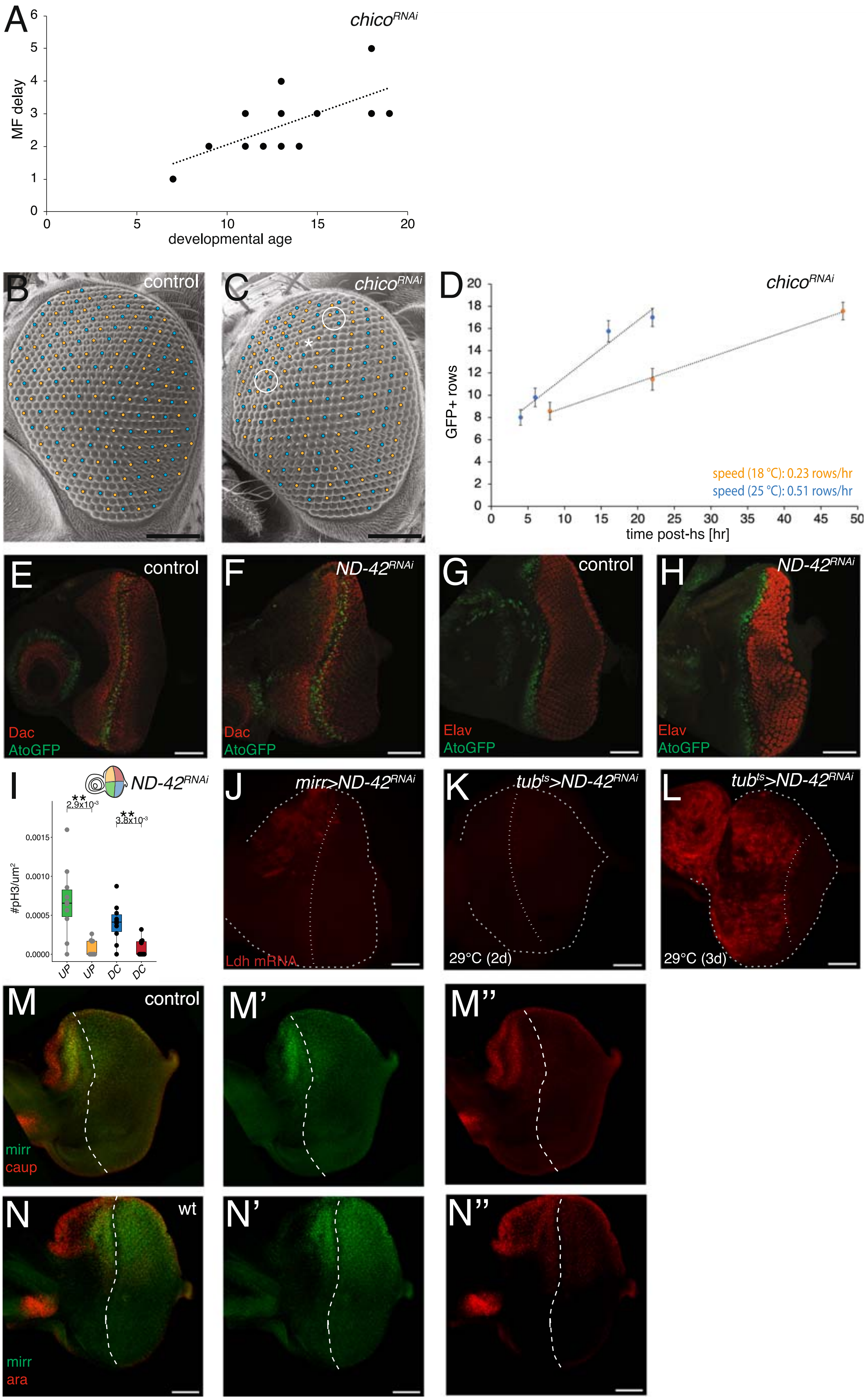
A
chico^RNAi
MF delay
developmental age
B control
C chico^RNAi
D chico^RNAi
GFP+ rows
time post-hs [hr]
speed (18 °C): 0.23 rows/hr
speed (25 °C): 0.51 rows/hr
E control
Dac
AtoGFP
F ND-42^RNAi
Dac
AtoGFP
G control
Elav
AtoGFP
H ND-42^RNAi
Elav
AtoGFP
I ND-42^RNAi
2.9x10^-3
3.8x10^-3
#pH3/um²
UP
UP
DC
DC
J mirr>ND-42^RNAi
Ldh mRNA
K tub^ts>ND-42^RNAi
29°C (2d)
L tub^ts>ND-42^RNAi
29°C (3d)
M control
mirr
caup
M'
M''
N wt
mirr
ara
N'
N''

 © The Author(s)

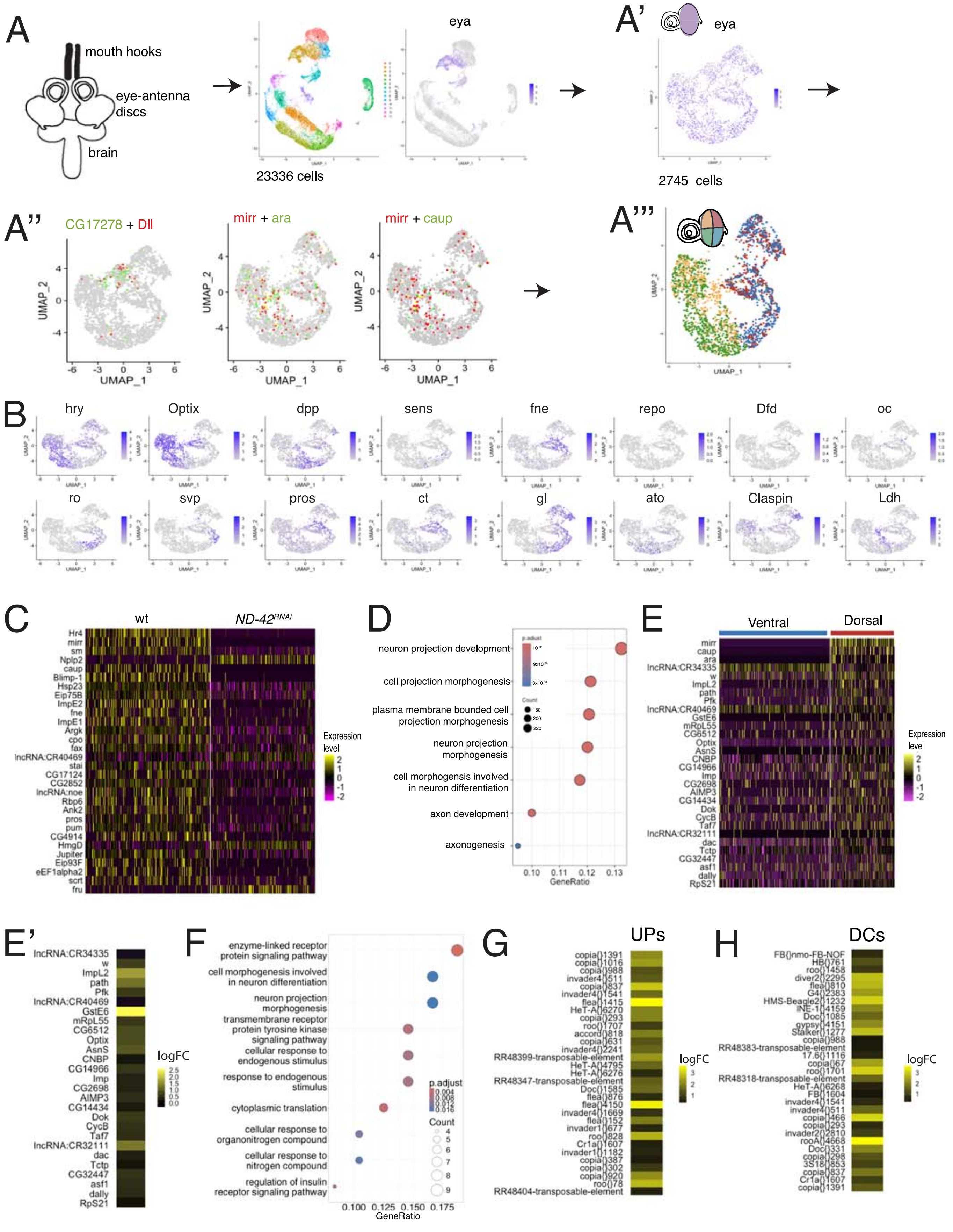
A
mouth hooks
eye-antenna discs
brain
23336 cells
eya
A'
eya
2745 cells
A''
CG17278 + Dll
mirr + ara
mirr + caup
UMAP_1
UMAP_2
A'''
B
hry
Optix
dpp
sens
fne
repo
Dfd
oc
ro
svp
pros
ct
gl
ato
Claspin
Ldh
C
wt
ND-42RNAi
Hr4
mirr
sm
Nplp2
caup
Blimp-1
Hsp23
Eip75B
ImpE2
fne
ImpE1
Argk
cpo
fax
lncRNA:CR40469
stai
CG17124
CG2852
lncRNA:noe
Rbp6
Ank2
pros
pum
CG4914
HmgD
Jupiter
Eip93F
eEF1alpha2
scrt
fru
Expression level
D
neuron projection development
cell projection morphogenesis
plasma membrane bounded cell projection morphogenesis
neuron projection morphogenesis
cell morphogenesis involved in neuron differentiation
axon development
axonogenesis
GeneRatio
E
Ventral
Dorsal
mirr
caup
ara
lncRNA:CR34335
w
ImpL2
path
Pfk
lncRNA:CR40469
GstE6
mRpL55
CG6512
Optix
AsnS
CNBP
CG14966
Imp
CG2698
AIMP3
CG14434
Dok
CycB
Taf7
lncRNA:CR32111
dac
Tctp
CG32447
asf1
dally
RpS21
Expression level
E'
logFC
F
enzyme-linked receptor protein signaling pathway
cell morphogenesis involved in neuron differentiation
neuron projection morphogenesis
transmembrane receptor protein tyrosine kinase signaling pathway
cellular response to endogenous stimulus
response to endogenous stimulus
cytoplasmic translation
cellular response to organonitrogen compound
cellular response to nitrogen compound
regulation of insulin receptor signaling pathway
p.adjust
Count
GeneRatio
G
UPs
logFC
H
DCs
logFC

Figure EV2. A scRNAseq analysis of the DC response to ETC inhibition.

(**A**, **B**) scRNAseq was performed on cells from dissected eye-brain complexes from control Oregon-R larvae (30,045 cells) and *mirr* > *ND-42*^{RNAi} larvae (23,336 cells). Clustering analysis identified one cluster marked by *eya*+ cells corresponding to the eye primordium (**A**, **A′**). Cells from this cluster (2745 and 2112 cells for the *mirr* > *ND-42*^{RNAi} and Oregon-R samples, respectively) were further clustered (**A″**), leading to the identification of a peripodial cell cluster based on *Dll* and *CG17278* gene expression (**A″**). This cluster was removed in all further analysis. The remaining UPs (1275 cells; color-coded green and orange) and DCs (1127 cells; color-coded red and blue) were identified based on marker gene expression (**A‴**, **B**). These eye cells were also categorized as *d* and *v* cells based on the expression of the *mirr, ara*, or *caup* genes (triple-negative cells were identified as *v* cells; see schematic in (**A‴**) for the color code). (**C**) Heatmap of the top 30 differentially expressed genes between wild-type *v* and *d* cells (wt, Oregon-R) and *mirr* > *ND-42*^{RNAi} control *v* cells (gene expression level color-coded as indicated). Genes were ranked by adjusted *P* values. A few ecdysone-regulated genes and differentiation markers appeared to be differentially expressed between these two populations of control cells. (**D**) Gene Ontology (GO) term enrichment analysis for biological pathways for the top 1781 genes. (**E**, **E′**) The top 30 differentially expressed genes between *ND-42*^{RNAi} *d* DCs ($n = 414$) and control *v* DCs ($n = 713$). DCs from *mirr* > *ND-42*^{RNAi} discs were ranked by adjusted *P* values, and expression levels were color-coded (**E**; see heatmap). Relative changes in averaged expression levels are shown as log2 Fold Change values (logFC; **E′**). The three genes used for clustering (*mirr*, *ara*, and *caup*) were removed from the analysis shown in (**E′**). (**F**) GO term enrichment analysis for biological pathways for the top 60 genes with *P* value < 0.05 in DCs. (**G**, **H**) The top 30 differentially expressed transposable elements (TE) in DCs ($n = 73$ TEs) and UPs ($n = 53$ TEs) were ranked by *P* values, and expression levels (logFC values) were color-coded as indicated. Two-sided *t* test (**D**, **F**).

 © The Author(s)

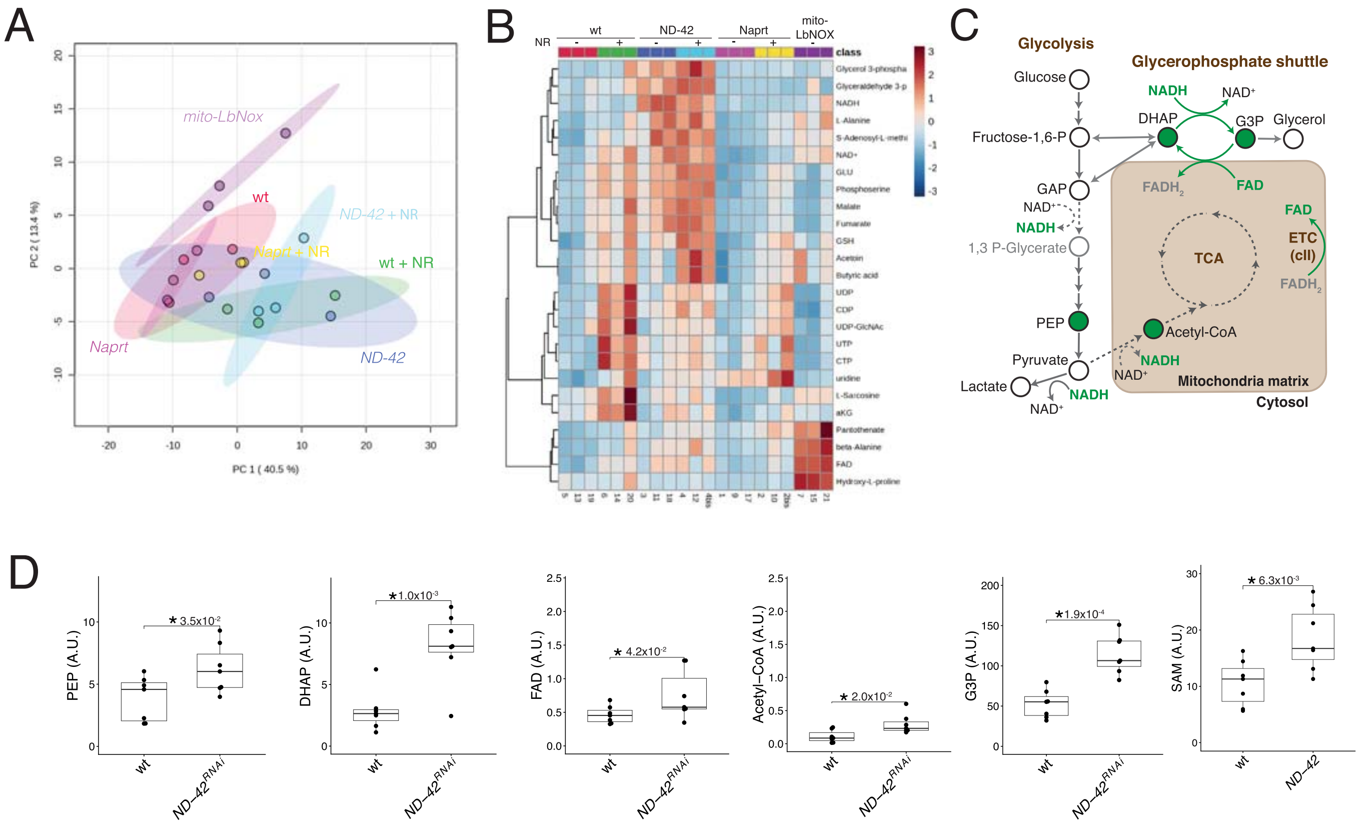

Figure EV3. A metabolomic analysis of cI inhibition and reduced NAD$^+$ recycling by LC/MS.

(**A**) PCA analysis of the metabolome (149 metabolites) of whole cell extracts prepared from *tub*^{ts} > +, *tub*^{ts} > *ND-42*^{RNAi}, *tub*^{ts}>*Naprt*^{RNAi}, and *tub*^{ts}>*mito-LbNOX* eye discs. These discs were dissected from larvae grown in fly food supplemented or not with NR (as indicated). Clustering analysis indicated that the *tub*^{ts}>*Naprt*^{RNAi} metabolome was distinct from those of all other samples. Also, the *tub*^{ts} > *ND-42*^{RNAi} metabolome differed from the *tub*^{ts} > + control metabolome in the absence of NR supplementation. In the presence of NR, the effect of *ND-42*^{RNAi} appeared to be more restricted. (**B**) Clustering analysis based on the top 25 differentially detected metabolites (abundance plotted as a heatmap). Differences in NAD, glycolysis, and TCA cycle metabolism were detected in the *tub*^{ts} > *ND-42*^{RNAi} metabolome relative to the *tub*^{ts} > + control metabolome. (**C**) Proposed changes in energy metabolism pathways based on the LC/MS results. Metabolites that showed a statistically significant increase upon *ND-42*^{RNAi} relative to wild-type condition appear in green (see Dataset EV2 for FC values). Enzymatic reactions proposed to be upregulated are shown as green arrows; those proposed to be down-regulated appear as dotted arrows. Limiting NAD$^+$ levels are proposed to result in substrate accumulation in reactions coupled to NAD$^+$ reduction, e.g., GAP. Increased DHAP and GAP levels are interpreted to suggest increased Glycerol Phosphate shuttle activity, promoting NAD$^+$ regeneration in the cytosol and reduction of Flavin Adenine Dinucleotide (FAD) in mitochondria ($FADH_2$ could then serve to fuel the ETC via the cII). High levels of Acetyl-CoA are proposed to accumulate due to defective TCA activity. (**D**) Plots showing the LC/MS results for the PhosphoEnolPyruvic acid (PEP), DHAP, FAD, Acetyl-CoA, G3P, and SAM ($n = 7$). The box plots show the median and the interquartile range from the 25th to 75th percentile; whiskers extend to data points within 1.5× the interquartile range of the lower and upper quartiles, representing the approximate minimum and maximum non-outlier values. Two-sided *t* test (**D**), *P* values: **<0.01.

© The Author(s)

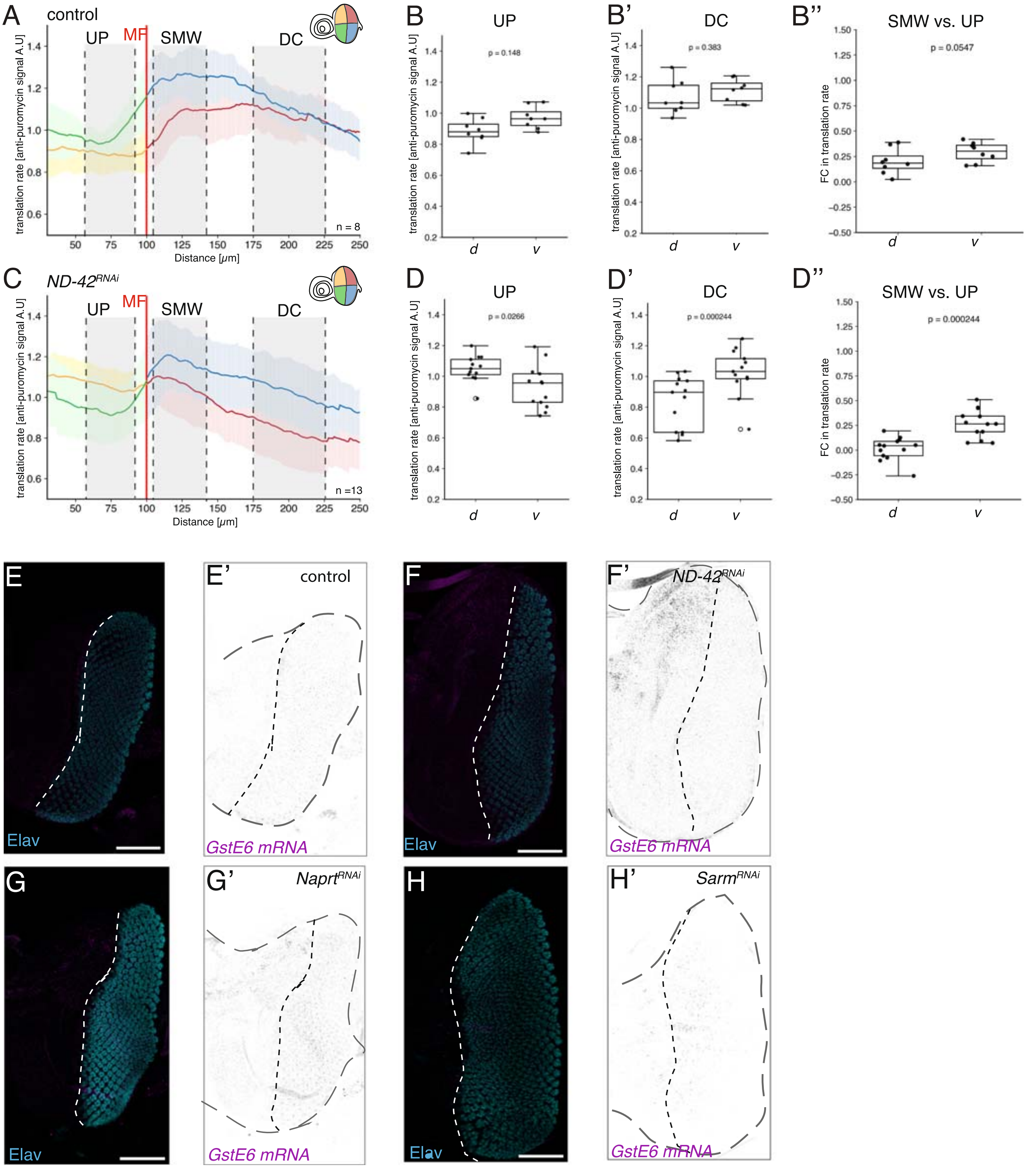
A
control
UP
MF
SMW
DC
translation rate [anti-puromycin signal A.U]
Distance [µm]
n = 8
B
UP
p = 0.148
translation rate [anti-puromycin signal A.U]
d
v
B'
DC
p = 0.383
B''
SMW vs. UP
p = 0.0547
FC in translation rate
C
ND-42RNAi
n =13
D
UP
p = 0.0266
D'
DC
p = 0.000244
D''
SMW vs. UP
p = 0.000244
E
Elav
E'
control
GstE6 mRNA
F
Elav
F'
ND-42RNAi
GstE6 mRNA
G
Elav
G'
NaprtRNAi
GstE6 mRNA
H
Elav
H'
SarmRNAi
GstE6 mRNA

Figure EV4. Translation rate analysis and ROS expression.

(**A–D″**) Translation rates were inferred by measuring the intensity of the anti-puromycin signal in *mirr* > + (control; $n = 8$) and *mirr* > *ND-42*^{RNAi} ($n = 13$) eye discs (**A**, **C**). An increased rate of translation was detected posterior to the MF in control discs (**A**), where InR-dependent cell growth was associated with the SMW (Kim et al, 2017); this region is defined here as SMW ([105–145] in (**A**, **C**)). No statistically significant differences were observed in control discs between *d* and *v* cells in UPs (**B**; defined here as [60–90]) and DCs (**B′**; [175–225]). Likewise, a similar increase in translation from UPs to the SMW region was detected in both *d* and *v* cells (**B″**). In contrast, a mild increase in protein synthesis in UPs (**D**), but a decrease in DCs (**D′**), were noted in *d* versus *v* cells in *ND-42*^{RNAi} eye discs. We further found that loss of cI activity abolished the increase in protein translation in the SMW region (**D″**), consistent with reduced cell proliferation (Fig. EV1I). The *P* values from the Wilcoxon are indicated. (**E–H′**) The silencing of the *ND-42* gene led to increased *GstE6* expression in the dorsal UPs of *mirr* > *ND-42*^{RNAi} discs (**F′**; smiFISH signal in black; compare with control discs in (**E**, **E′**), and to the *v* cells in (**F′**)), indicative of increased ROS. In contrast, the silencing of the *Naprt and Sarm* genes had little effect of *GstE6* expression (**G–H′**), suggesting that these perturbations have a minor effect on ROS levels. Nevertheless, a clear MF delay phenotype was seen in all three RNAi conditions (Elav, cyan; the MF and the disc outline are indicated by dotted lines), suggesting that *GstE6* expression, i.e., ROS levels, did not correlate well with MF delay. The box plots show the median and the interquartile range from the 25th to 75th percentile; whiskers extend to data points within 1.5× the interquartile range of the lower and upper quartiles, representing the approximate minimum and maximum non-outlier values. Two-sided *t* test.

© The Author(s)

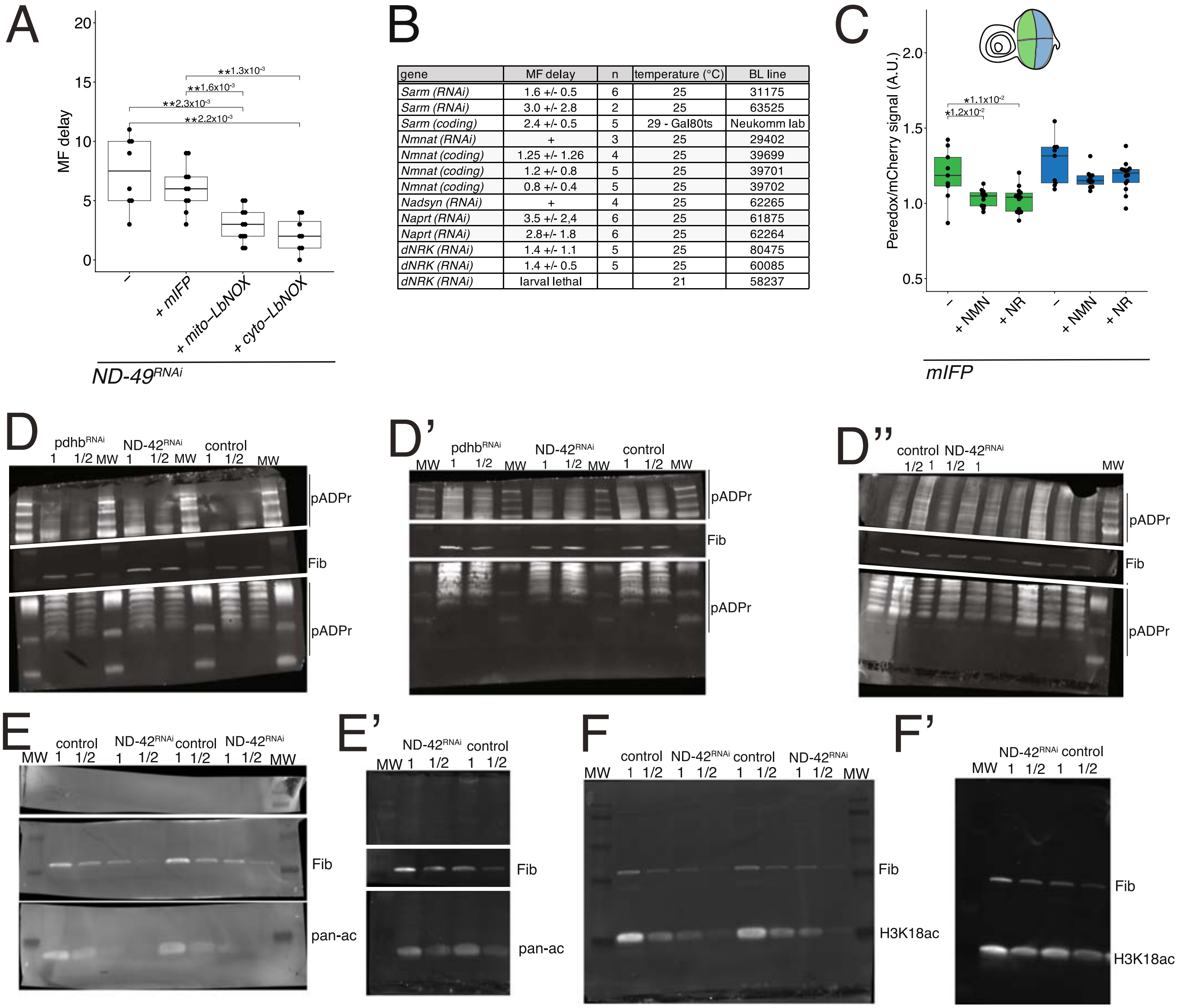

gene	MF delay	n	temperature (°C)	BL line
Sarm (RNAi)	1.6 +/- 0.5	6	25	31175
Sarm (RNAi)	3.0 +/- 2.8	2	25	63525
Sarm (coding)	2.4 +/- 0.5	5	29 - Gal80ts	Neukomm lab
Nmnat (RNAi)	+	3	25	29402
Nmnat (coding)	1.25 +/- 1.26	4	25	39699
Nmnat (coding)	1.2 +/- 0.8	5	25	39701
Nmnat (coding)	0.8 +/- 0.4	5	25	39702
Nadsyn (RNAi)	+	4	25	62265
Naprt (RNAi)	3.5 +/- 2,4	6	25	61875
Naprt (RNAi)	2.8+/- 1.8	6	25	62264
dNRK (RNAi)	1.4 +/- 1.1	5	25	80475
dNRK (RNAi)	1.4 +/- 0.5	5	25	60085
dNRK (RNAi)	larval lethal		21	58237

Figure EV5. NAD$^+$, cellular redox, and speed of progression of the MF.

(**A**) MF delay analysis showing that the expression of cytosolic or mitochondrial LbNOX was sufficient to reduce the delay resulting from the silencing of the *ND-49* gene: *mirr > ND-49^{RNAi}*: 7.0 + / −3.4 ($n = 5$); *mirr > ND-49^{RNAi} + mIFP*: 6.1 + / −2.0 ($n = 10$); *mirr > ND-49^{RNAi} + mito-LbNOX*: 2.9 + / −1.4 ($n = 11$); *mirr > ND-49^{RNAi} + cyto-LbNOX*: 2.1 + / −1.5 ($n = 8$). (**B**) MF delay analysis of NAD metabolism gene (see Fig. 7C). '+' indicated that the number of ommatidial rows were not counted as discs looked clearly similar to wt. (**C**) Analysis of the Peredox normalized intensity ratio in *mirr > mIFP* ($n = 9$) eye discs indicated that addition of NMN ($n = 8$) and NR ($n = 11$) lowered the cellular redox in UPs, but not DCs. (**D–D''**) Western blot analysis of PARylation in control and *ND-42^{RNAi}* eye discs. Fibrillarin (Fib) was used to normalize the poly-ADPr signal (measured as the sum of the signals from top and bottom parts of the blots). Each sample was loaded twice (with a 2-fold difference in volume; MW, Molecular Weight; same blot as in Fig. 7H). (**E–F'**) Western Blot analysis of acetylation in control and *ND-42^{RNAi}* eye discs. Fibrillarin (Fib) was used to normalize the pan-acetylation (pan-ac; **E–E'**) and H3K18ac signals (**F–F'**). Each sample was loaded twice (with a twofold difference in volume; MW, Molecular Weight; same blot as in Fig. 5M–N'). The box plots show the median and the interquartile range from the 25th to 75th percentile; whiskers extend to data points within 1.5× the interquartile range of the lower and upper quartiles, representing the approximate minimum and maximum non-outlier values. Wilcoxon test (**A**, **C**), *P* values: *<0.05; **<0.01.

 © The Author(s)
