# Supplementary material for: NAD+ supply and redox state limit developmental speed in the Drosophila eye
Source: EMBO J. 2026 May 13;45(12):4094–123. doi: 10.1038/s44318-026-00801-4 (PMC13270060; doi:10.1038/s44318-026-00801-4)
Supplement: Supplementary file 1 — Peer Review File [file 44318_2026_801_MOESM1_ESM.pdf]

THE EMBO JOURNAL

NAD+ supply and redox state limit developmental speed in the Drosophila eye

nisha veits, Yuting Guo, Jingjing He, Khallil Mazouni, Ivan Nemazanyy, Martin Bres, cara picciotto, Claire Mestdagh, Yan Yan, and Francois Schweisguth

Corresponding author(s): Francois Schweisguth (fschweis@pasteur.fr)

Review Timeline:

Submission Date:	1st Sep 25
Editorial Decision:	29th Sep 25
Revision Received:	3rd Mar 26
Editorial Decision:	13th Apr 26
Revision Received:	14th Apr 26
Accepted:	24th Apr 26

Editor: Ieva Gailite

Transaction Report:

(Note: With the exception of the correction of typographical or spelling errors that could be a source of ambiguity, letters and reports are not edited. Depending on transfer agreements, referee reports obtained elsewhere may or may not be included in this compilation. Referee reports are anonymous unless the Referee chooses to sign their reports.)

1st Editorial Decision 29th Sep 2025

Dear Dr. Schweisguth,

Thank you for submitting your manuscript for consideration by the EMBO Journal. We have now received comments from three reviewers, which are included below for your information.

As you will see, reviewers #1 and #2 are generally positive in their assessment and appreciate the contribution of the study to the research field, while reviewer #3 is more critical on this aspect. Additionally, all reviewers indicate a number of concerns that would need to be addressed in a revised version. Based on the positive assessments of reviewers #1 and #2, I would like to invite you to address the comments of all reviewers in a revised manuscript. I realise that some of the points, e.g., points 6-8 by reviewer #3, are more far-reaching and can be addressed with appropriate discussion. I think that it would be useful to discuss the revision in more detail via email or phone/videoconferencing - please let me know which option you prefer.

We generally allow three months as standard revision time, which can be extended to six months in the case of major revisions. Should you foresee a problem in meeting this deadline, please let us know in advance to discuss an extension.

As a matter of policy, competing manuscripts published during this period will not negatively impact on our assessment of the conceptual advance presented by your study. However, please contact me as soon as possible upon publication of any related work to discuss the appropriate course of action.

When preparing your letter of response to the referees' comments, please bear in mind that this will form part of the Review Process File and will therefore be available online to the community. For more details on our Transparent Editorial Process, please visit our website: https://www.embopress.org/page/journal/14602075/authorguide#transparentprocess. Please also see the attached instructions for further guidelines on preparation of the revised manuscript.

Please feel free to contact me if have any further questions regarding the revision. Thank you for the opportunity to consider your work for publication, and I look forward to discussing your revision with you.

With best wishes,

Ieva Gailite

Ieva Gailite, PhD
Senior Scientific Editor
The EMBO Journal
Meyerhofstrasse 1
D-69117 Heidelberg
Tel: +4962218891309
i.gailite@embojournal.org

Instructions for preparing your revised manuscript:

Please make sure you upload a letter of response to the referees' comments together with the revised manuscript.

Please also check that the title and abstract of the manuscript are brief, yet explicit, even to non-specialists.

When assembling figures, please refer to our figure preparation guideline in order to ensure proper formatting and readability in print as well as on screen:
https://bit.ly/EMBOPressFigurePreparationGuideline
See also guidelines for figure legends: https://www.embopress.org/page/journal/14602075/authorguide#figureformat

At EMBO Press we ask authors to provide source data for the main manuscript figures. You will receive a separate email with instructions for providing source data with your revised manuscript, including how to upload and organize the files.

IMPORTANT: When you send the revision we will require
- a point-by-point response to the referees' comments, with a detailed description of the changes made (as a word file).
- a word file of the manuscript text.
- individual production quality figure files (one file per figure)
- a complete author checklist, which you can download from our author guidelines (https://www.embopress.org/page/journal/14602075/authorguide).
- Expanded View files (replacing Supplementary Information)

Please see out instructions to authors
https://www.embopress.org/page/journal/14602075/authorguide#expandedview
- a Reagents and Tools Table as part of the Methods section, which can be downloaded from our author guidelines (https://www.embopress.org/page/journal/14602075/authorguide#structuredmethods)

Please remember: Digital image enhancement is acceptable practice, as long as it accurately represents the original data and conforms to community standards. If a figure has been subjected to significant electronic manipulation, this must be noted in the figure legend or in the 'Materials and Methods' section. The editors reserve the right to request original versions of figures and the original images that were used to assemble the figure.

Further information is available in our Guide For Authors: https://www.embopress.org/page/journal/14602075/authorguide

We realize that it is difficult to revise to a specific deadline. In the interest of protecting the conceptual advance provided by the work, we recommend a revision within 3 months (28th Dec 2025). Please discuss the revision progress ahead of this time with the editor if you require more time to complete the revisions. Use the link below to submit your revision:

https://emboj.msubmit.net/cgi-bin/main.plex

Referee #1:

Veits et al. investigate the mechanisms that regulate developmental timing using the developing eye of Drosophila. They develop an assay taking advantage of the unique spatial and temporal distribution of differentiating cells in the eye imaginal disc, the so-called migration morphogenetic furrow. This enables them to use the number of differentiated ommatidial furrows as a faithful proxy of the speed of progression of differentiation, which can be manipulated genetically in two independent dorsal and ventral subdomains. Using this system they RNAi screen for genes involved in developmental timing, focusing on mitochondria/metabolic genes given their recent implication in other systems. They find that mitochondria ETC genes and other genes involved in NAD/NADH balance to be required for normal speed of the MF migration. The using further genetic manipulations and metabolic profiling they identify NAD as the main candidate metabolite limiting the process (instead of redox balance).

Overall this is a very elegant and original piece of work. It focuses on a timely and important topic, and constitutes the first clear in vivo demonstration of the requirement of mitochondria metabolism in the scaling of developmental timing.

I have only a few comments/questions:

1. The authors quantify the delay by counting the difference in number of ommatidia in dorsal vs ventral compartments. From the data shown it seems that the total amount of ommatidia is not always constant across conditions (Fig 1C,D,E for instance): did the authors try to account for this by normalizing the difference in ommatidia to their total number? Does that change the results in any conditions?

2. The authors should show the Western blots of PARylation that were quantified in Figure 7H. It would be interesting to also look for acetylation patterns in the same way, which should be easily feasible with ad hoc antibody.

3. The levels of Acetyl-CoA appear to be increased in the ND-42RNAi condition. Are these mitochondrial or cytosolic levels? Was this result expected and how could it relate to acetylation patterns? These questions should be discussed more thoroughly.

Referee #2:

Veits and colleagues report a critical role for cellular metabolism, and specifically the levels of NAD+, in regulating the developmental speed of neuronal differentiation using the fly retina as a model system. The topic is important and timely (no pun intended) and the work is elegant leveraging the unique power of fly genetics. The data are convincing, clear and well presented. The conclusions represent an important advance in our understanding of the role of cellular metabolism regulates developmental tempo. I support publication in the EMBO J without significant reservations.

Having said this, I have a few suggestions for the authors to consider. I believe addressing these concerns by textual changes, and new data where possible, will strengthen the conclusions. These points do not detract from the excellence of the work or my enthusiasm for its publication.

Key points:

1- Given that the evidence supports and extends recent models for the role of NAD+ and/or the NADH/NAD+ in regulating developmental speeds across species and tissues, the abstract merits ending with a more general statement about the generality of the findings.

2- The authors state that "eye patterning" is unaffected. However, the data shown reflect global patterning. It would be interesting to examine whether the initial "temporal patterning" of ommatidial development is affected (R8>R2/5>R3/4). Are the cell fate of photoreceptors correctly specified and in the direct temporal order? Is there a delay in the second mitotic wave and the generation of late born photoreceptors (R1, 6 and 7)? Or does everything simply scale with the speed of the furrow. This can be easily examined by simple marker analysis. If there is no effect, this would show that the global timing of neurogenesis can be uncoupled from the temporal progression of sequential cell fate specification.

3- The main concern I have is some confusion with regards to the compensatory role of Glycolysis and the consequently the potential role of ATP production.
3.1: The authors show that ETC inhibition via cI kick-down does not reduce ATP levels because glycolysis "kicks in" to compensate. Why then does glycolysis not compensate for cIII inhibition by AntA?
3.2: Along the same lines, how can ND42 re-expression rescue the effects of AntA given that ND-42 acts in cI and AntaA inhibits cIII, which should be downstream, as far as I understand?
3.3: Finally, if the delay observed upon cI inhibition is explainable by reduced protein synthesis and other consequences of reduction in NAD+, how certain are the authors that this is not due to reduced ATP production, given that when glycolysis is inhibited the phenotype is exasperated? Is it not possible that the rate - rather than total amounts - of ATP production plays a critical role?
Addressing these issues, at least by explicitly discussing them would alleviate some of the confusion.

Additional points:
page 4: The statement that Atonal is required for eye differentiation should cite the original work by Jarman and colleagues.
Page 9: "bearkdowns" should be "breaks down"

Referee #3:

In this manuscript the authors used mitochondrial complex I inhibition induced MF delay as an in vivo setting to address the important question of how developmental rate is controlled. They conclude that NAD+ availability and redox state constraint developmental speed in Drosophila eye. This conclusion is consistent with previous studies explaining the species-specific development rates in humans and mice. Although this study uses the Drosophila eye as an in vivo system to investigate mechanisms of developmental rate control, the novel insights is limited compared to what is already in the literature, and the results need further clarification.

1. The key system used in the manuscript is regional/cell type specific RNAi of ND-42, a key component of mitochondrial complex I. The organization of the photoreceptors is disorganized (Fig. 2B). Is it not clear how the rows of the photoreceptors were accurately counted, and if the loss of photoceptors is caused by cell death or other defects instead of developmental delay.

2. Inhibition of complex I is known to generates excess ROS. The potential role of ROS is not addressed. Although the study focuses on NAD+ availability, NAD+/NADH metabolism is known to affect ROS as well.

3. The main conclusion that NAD+ availability is a key constraint of developmental speed is not well supported. In some assays, for example Fig. 4K', NAD+ level in ND-42 RNAi is not reduced but rather shows an obvious trend of increase. In other assays, only NAD+/NADH was presented. In all figures showing NAD+/NADH ratio, the NAD+ and NADH levels should be individually presented.

4. It is not clearly explained why NR NMN treatments have different effects on NAD+/NADH ratio in UP and DC cells.

5. In many experiments, for example Fig. 2D, 5H, 5I, 7I, the number of UAS transgenes is not equal between genotypes, making it difficult to compare the study outcome, as the attenuation of ND42 RNAi effect could be caused by dilution of GAL4 by the extra UAS transgenes.

6. It is not clear in what in vivo physiological/stress condition the mechanism of ND42 RNAi-induced developmental delay is relevant.

7. It is not clear what downstream pathways depend on NAD+ availability to control developmental rate. In previous studies in mammals, protein synthesis was established as a key mechanism. Is this conserved in flies? Are Sirtuins or PARPs involved?

8. Why the MF delay induced during development has no consequence on adult eye morphology. What mechanisms compensate for this developmental defect?

Referee #1:

Veits et al. investigate the mechanisms that regulate developmental timing using the developing eye of Drosophila. They develop an assay taking advantage of the unique spatial and temporal distribution of differentiating cells in the eye imaginal disc, the so-called migration morphogenetic furrow. This enables them to use the number of differentiated ommatidial furrows as a faithful proxy of the speed of progression of differentiation, which can be manipulated genetically in two independent dorsal and ventral subdomains. Using this system they RNAi screen for genes involved in developmental timing, focusing on mitochondria/metabolic genes given their recent implication in other systems. They find that mitochondria ETC genes and other genes involved in NAD/NADH balance to be required for normal speed of the MF migration. The using further genetic manipulations and metabolic profiling they identify NAD as the main candidate metabolite limiting the process (instead of redox balance).

Overall this is a very elegant and original piece of work. It focuses on a timely and important topic, and constitutes the first clear in vivo demonstration of the requirement of mitochondria metabolism in the scaling of developmental timing.

We thank reviewer #1 for her/his very positive appreciation of our work.

I have only a few comments/questions:

1. The authors quantify the delay by counting the difference in number of ommatidia in dorsal vs ventral compartments. From the data shown it seems that the total amount of ommatidia is not always constant across conditions (Fig 1C,D,E for instance): did the authors try to account for this by normalizing the difference in ommatidia to their total number? Does that change the results in any conditions?

The referee is correct: we did not normalize the difference by the total number of ommatidia and, as the referee may expect, the difference varies with the total number of ommatidial rows (consistent with a speed defect). The difference was routinely scored in wandering larvae in which eye discs showed 20+/-10 rows of ommatidia, so normalization should have a minimal impact. Indeed, the same genotype (*ND-42^{RNAi}*) scored by three different student/post-docs gave very similar results. Nevertheless, the measured difference should only be considered as an approximation of the severity of the phenotype. We clarified this in the text (methods) and added a panel to Figure S1 to show that the measured difference increases with the total number of rows in *chico^{RNAi}* discs (Fig S1A)

2. The authors should show the Western blots of PARylation that were quantified in Figure 7H. It would be interesting to also look for acetylation patterns in the same way, which should be easily feasible with ad hoc antibody.

We now show the PARylation WBs in Fig 7H (all blots are shown in Fig S8D-D'').

We agree with the reviewer about protein acetylation. We studied Histone acetylation by Western Blots using an anti-H3K18ac and pan-acetylation antibodies. Despite, the increase of total acetyl-CoA levels, we observed a decrease level of Histone acetylation (see Fig 5M-N' and in Fig S8E-F'). While the lack of correlation between acetyl-CoA levels and Histone acetylation may seem paradoxical, we suggest that this might reflect an improper balance of acetyl-CoA between mitochondria and nuclei (a point raised by the referee below). Further studies (and tools!) will be needed to address in full the underlying biology. We believe that this goes beyond the scope of the current paper.

3. The levels of Acetyl-CoA appear to be increased in the ND-42RNAi condition. Are these mitochondrial or cytosolic levels? Was this result expected and how could it relate to acetylation patterns? These questions should be discussed more thoroughly.

Thanks for raising these interesting points! Since there is no fluorescent acetyl-CoA sensor available in *Drosophila*, we do not know whether the increase in acetyl-CoA levels is restricted to mitochondria (this is, however, possible considering the strong increase in glycolysis activity and the presumed loss of TCA activity). Of note, the observed decrease in Histone acetylation would be consistent with a mitochondria-specific increase in acetyl-CoA. We note that protein acetylation in mitochondria is largely non-enzymatic (with acetylation being associated with reduced OxPhos activity), while

Histone acetylation in the nucleus is largely enzymatic. We discuss in the manuscript the possibility that increased acetyl-CoA levels are restricted to mitochondria.

Referee #2:

Veits and colleagues report a critical role for cellular metabolism, and specifically the levels of NAD+, in regulating the developmental speed of neuronal differentiation using the fly retina as a model system. The topic is important and timely (no pun intended) and the work is elegant leveraging the unique power of fly genetics. The data are convincing, clear and well presented. The conclusions represent an important advance in our understanding of the role of cellular metabolism regulates developmental tempo. I support publication in the EMBO J without significant reservations.

We thank reviewer #2 for highlighting the importance of our work.

Having said this, I have a few suggestions for the authors to consider. I believe addressing these concerns by textual changes, and new data where possible, will strengthen the conclusions. These points do not detract from the excellence of the work or my enthusiasm for its publication.

Key points:

1- Given that the evidence supports and extends recent models for the role of NAD+ and/or the NADH/NAD+ in regulating developmental speeds across species and tissues, the abstract merits ending with a more general statement about the generality of the findings.

We thank the referee for this suggestion. The revised abstract ends with the following sentence : Our findings therefore extend to *Drosophila* the key role played by the NADH/NAD^+ in the regulation of developmental speed and highlight the importance of NAD^+ availability for this regulation in *Drosophila*.

2- The authors state that "eye patterning" is unaffected. However, the data shown reflect global patterning. It would be interesting to examine whether the initial "temporal patterning" of ommatidial development is affected (R8>R2/5>R3/4). Are the cell fate of photoreceptors correctly specified and in the direct temporal order? Is there a delay in the second mitotic wave and the generation of late born photoreceptors (R1, 6 and 7)? Or does everything simply scale with the speed of the furrow. This can be easily examined by simple marker analysis. If there is no effect, this would show that the global timing of neurogenesis can be uncoupled from the temporal progression of sequential cell fate specification.

We thank the reviewer for raising this interesting question. The referee asks whether the speed of neuronal differentiation (assayed using markers of photoreceptor differentiation) can be uncoupled from the speed of progression of the MF. This is an interesting question. We present below what are, in our view, the expected results in the two scenarii (coupled vs uncoupled).

In the schematics below, we consider two fronts, one corresponding to the MF (blue) and the other to the front of neurogenesis (red). In wild-type discs, the two fronts go to the same speed with a constant time delay. If the speed of the MF is reduced but if neurogenesis progresses at the same speed (constant time delay), then we consider that both fronts are uncoupled. In contrast, if the speed of the MF is reduced and if the speed of neurogenesis is similarly decreased (increased time delay), then we will consider that the two fronts are coupled. If we now translate these in terms of distance, we expect the following spatial patterns:

• a constant time delay with a slow-moving front will shorten the distance between the MF and the neurogenesis front in a manner proportional to the speed difference in the progression of the MF (~30% for *ND-42*^{RNAi}, based on our speed assay)

• a time delay reduced in a manner proportional to to the speed difference in the progression of the MF will maintain constant the distance between the MF and the neurogenesis front.

To reliably detect a 30% difference, we would need to look at a long time delay. Looking at the onset of photoreceptor differentiation, which was detected ~3 rows behind the MF using Elav as a neuronal marker (both in control and *mirr>ND-42*^{RNAi} discs; not shown), did not provide sufficient spatial resolution to test for possible uncoupling. We therefore used Prospero that marks R7 cells, 7-8 rows

behind the MF (7.6 +/-0.7 in control discs, n=10). Preliminary analysis suggested that the distance between the Pros+ rows and the MF did not significantly differ between the dorsal and ventral compartments of *mirr>ND-42*^{RNAi} discs (dorsal, 6.6 +/-0.5 and ventral, 7.3 +/- 0.3, n=8), suggestive of coupling. This preliminary result is consistent with the view that this metabolic perturbation did not perturb only the speed of progression of the MF but likely affected the speed of many other cellular and developmental processes in the eye discs. We would, however, prefer to not include this preliminary observation in our manuscript for the following reasons : i) we do not wish to make a complicated argument for a conclusion that may not look too surprising ; ii) this analysis is not entirely satisfying since R7 specification occurs at the end of a long multi-step process of cell specification (formalized here as a single time constant).

3- The main concern I have is some confusion with regards to the compensatory role of Glycolysis and the consequently the potential role of ATP production.
3.1: The authors show that ETC inhibition via cI kick-down does not reduce ATP levels because glycolysis "kicks in" to compensate. Why then does glycolysis not compensate for cIII inhibition by AntA?
The observed effect suggests that the time scale of metabolism rewiring (i.e. increasing glycolysis upon ETC inhibition via changes in gene expression) is likely to much longer (hours) than the time scale of the acute inhibitory effect of AntA (mins).

3.2: Along the same lines, how can ND42 re-expression rescue the effects of AntA given that ND-42 acts in cI and AntaA inhibits cIII, which should be downstream, as far as I understand?
Yes, AntA inhibits cIII, whereas ND-42 is a cI subunit. If we understand the reviewer, this logic would be correct if we had shown that over-expression of ND-42 would rescue the effect of AntA in wild-type discs. What we showed is that the effect of AntA on cIII requires cI activity (rescue of *ND-42*^{RNAi} by ND-42*).

3.3: Finally, if the delay observed upon cI inhibition is explainable by reduced protein synthesis and other consequences of reduction in NAD+, how certain are the authors that this is not due to reduced ATP production, given that when glycolysis is inhibited the phenotype is exasperated? Is it not possible that the rate - rather than total amounts - of ATP production plays a critical role?
This is a good point. It is conceivable that loss of ND-42 results in a reduced flux of ATP production. This is now discussed in the Discussion section: While ATP levels appeared to remain unchanged, it is possible that the rate of ATP synthesis was altered upon cI perturbation. Consistent with this, increased ATP consumptions was detected upon cI inhibition in cultured human cells (Sercel et al., 2024; Sturm et al., 2023). Whether cI inhibition affects the flux of ATP remains to be investigated.

Addressing these issues, at least by explicitly discussing them would alleviate some of the confusion.
Additional points:
page 4: The statement that Atonal is required for eye differentiation should cite the original work by Jarman and colleagues.
Good point, thanks !

Page 9: "bearkdowns" should be "breaks down"
corrected

Referee #3:
In this manuscript the authors used mitochondrial complex I inhibition induced MF delay as an in vivo setting to address the important question of how developmental rate is controlled. They conclude that NAD+ availability and redox state constraint developmental speed in Drosophila eye. This conclusion is consistent with previous studies explaining the species-specific development rates in humans and mice. Although this study uses the Drosophila eye as an in vivo system to investigate mechanisms of developmental rate control, the novel insights

is limited compared to what is already in the literature, and the results need further clarification.
1. The key system used in the manuscript is regional/cell type specific RNAi of ND-42, a key component of mitochondrial complex I. The organization of the photoreceptors is disorganized (Fig. 2B). Is it not clear how the rows of the photoreceptors were accurately counted, and if the loss of photoceptors is caused by cell death or other defects instead of developmental delay.
We acknowledge (here and in the paper) that counting can only be performed when the pattern of ommatidia is not too disorganized. As discussed above (point #1, reviewer #1), the measured difference should only be considered as an approximation of the severity of the phenotype. Nevertheless, scoring the same genotype (*ND-42*^{RNAi}) by different student/post-docs gave very similar results. So, our results are reproducible. Moreover, as indicated in the Methods, blind counting was performed in many of our experiments. We are not planning, however, to study the basis of pattern disorganization (which might result from defective patterning at the MF, defects posterior to the MF due to a limiting number of cells due to reduced proliferation and/or cell death, etc…).

2. Inhibition of complex I is known to generates excess ROS. The potential role of ROS is not addressed. Although the study focuses on NAD+ availability, NAD+/NADH metabolism is known to affect ROS as well
The referee is correct, and we indeed cited relevant papers showing that cI inhibition led to increase ROS in flies. Additionally, our transcriptomic data suggested that ROS generation is increased, as also noted in our paper.
To further study the antioxidant response, we used GstE6 expression as a ROS reporter. As explained in our revised manuscript: GstD1 is a commonly used marker for elevated Radical Oxygen Species (ROS) levels (Sykiotis and Bohmann, 2008). While GstD1 was expressed at higher levels in *ND-42*^{RNAi} UPs (logFC=0.33, Table S3), increased up-regulation was seen for GstE6 (logFC=1.6). We therefore used GstE6 as a ROS reporter and showed that the loss of cI activity led to increased *GstE6* expression (Fig S7A-B'), indicative of high ROS levels as observed earlier (Owusu-Ansah et al., 2013).

We next examined whether ROS levels, inferred from the antioxidant response (GstE6), correlated with slow developmental speed. While inhibition of *ND-42* (MF delay: 3.6 +/- 1.1) was associated with increased GstE6 expression, loss of *Naprt* (MF delay: 3.5+/-2.4) and *Sarm* (MF delay: 3.0+/-2.0), two enzymes involved in the NAD$^+$ salvage pathway, did not lead to GstE6 up-regulation in UPs (only moderate expression was detected in peripodial upon *Naprt* inhibition). Thus, this antioxidant response, hence presumably ROS levels, did not always correlate with slow developmental speed. Thus, while ROS might contribute to the metabolic rewiring that eventually leads to a MF delay, our data did not indicate that ROS plays a central role in the timely progression of the MF in the *Drosophila* eye.

3. The main conclusion that NAD+ availability is a key constraint of developmental speed is not well supported. In some assays, for example Fig. 4K', NAD+ level in ND-42 RNAi is not reduced but rather shows an obvious trend of increase. In other assays, only NAD+/NADH was presented. In all figures showing NAD+/NADH ratio, the NAD+ and NADH levels should be individually presented.
We respectfully disagree with the referee. All experimental perturbations shown (or predicted) to increase NAD$^+$ levels (LbNOX or Ndi1 expression, food supplementation with NR/NMN) suppressed the MF delay, whereas perturbations shown (or predicted) to decrease NAD$^+$ levels (loss of Naprt and Sarm activities, loss of Ldh in the context of ETC inhibition) led to (or enhanced) a MF delay. While many of these perturbations also perturb the NADH/NAD$^+$ ratio (see Fig 7F'',K-N), there are two contexts (*Naprt*^{RNAi}+NR and *ND-42*^{RNAi}+NR; see Fig 7L,M) for which changes in developmental speed resulting from a perturbation increasing NAD$^+$ availability (+NR) were not associated with a significant change in NADH/NAD$^+$ ratio in UPs. The simplest interpretation of our data is that NAD$^+$ levels can limit developmental speed. The referee is correct about the level of NAD$^+$ in *ND-42*^{RNAi} discs, i.e. an increase in total NAD$^+$ can be noted (note that this increase, shown in Fig 4K', is not statistically

significant). As mentioned in our manuscript (Results section), this putative increase could be interpreted as a compensatory response of the cells to the strong demand for free NAD^+ (shown to be limiting upon in *ND-42*^{RNAi}). Thus, absolute levels may not necessarily be a good proxy for availability.

Otherwise, all NAD^+ and NADH values measured by Glow assay and LC-MS were reported separately. However, the SoNar and Peredox biosensors directly report ratio values. These biosensors did not measure the levels of NAD^+ and NADH.

4. It is not clearly explained why NR NMN treatments have different effects on NAD+/NADH ratio in UP and DC cells.

The referee asks why UPs and DCs respond differently to the NR/NMN treatment, likely referring to the data now shown in Fig 7M.

First, we have now studied in more details the activity of key metabolic pathways in UPs (that grow and proliferate) vs DCs (that are mostly post-mitotic and differentiate) in wild-type discs. We found that Glycolysis and PPP were more active in UPs, consistent with high anabolic activities (see new Fig 5F-H'). Upon ETC loss, UPs and DCs also showed strong metabolic differences. This includes increased glycolysis, antioxidant response, amino acid transporter activity etc… (see Fig 5). It can therefore be expected that cells that are in different metabolic states show a difference in their response to NR/NMN.

Second, the NADH/NAD+ ratio can vary upon addition of NR/NMN (see Fig S8: a lower NADH/NAD+ ratio was observed upon NR/NMN treatment in wild-type discs, but only in UPs). In Fig 7N, we showed that the NADH/NAD^+ ratio remained constant in *ND-42*^{RNAi} UPs, but not in *ND-42*^{RNAi} DCs, upon NR/NMN treatment. We interpret these results to suggest that the NR/NMN treatment provides enough NAD^+ to respond to the moderate demand for NAD^+ in DCs. In contrast, this treatment did not provide enough NAD^+ to respond to the elevated energy demand of UPs, hence leading to persistently high NADH/NAD^+ ratio. Thus, the observed difference in the response to NR/NMN would reflect a difference in metabolic activities.

This interpretation is now provided in the Results section: In contrast, adding NR and NMN reduced the NADH/NAD^+ ratio in *ND-42*^{RNAi} DCs (Fig 7M). This suggested that the NR/NMN treatment provides enough NAD^+ to respond to the moderate demand for NAD^+ in DCs, but not in UPs, possibly because the energy demand in *ND-42*^{RNAi} UPs was higher, hence leading to persistently high NADH/NAD^+ ratio.

5. In many experiments, for example Fig. 2D, 5H, 5I, 7I, the number of UAS transgenes is not equal between genotypes, making it difficult to compare the study outcome, as the attenuation of ND42 RNAi effect could be caused by dilution of GAL4 by the extra UAS transgenes.

This is a good point. We repeated the experiments shown in Figs 2D, 5I and 7I by adding one copy of a *UAS-FLP or UAS-mIFP* transgene as controls. Similar results were obtained, hence confirming our conclusions.

The effect shown in Fig 5H could not result from a difference in transgene copy number of UAS. We, therefore, did no repeat this control.

6. It is not clear in what in vivo physiological/stress condition the mechanism of ND42 RNAi-induced developmental delay is relevant.

The genetic perturbations studied here are not meant to mimic changes in physiological conditions (e.g. starvation, low temperature).

7. It is not clear what downstream pathways depend on NAD+ availability to control developmental rate. In previous studies in mammals, protein synthesis was established as a key mechanism. Is this conserved in flies? Are Sirtuins or PARPs involved?

These are important questions.

First, we have examined the rate of protein synthesis in *ND-42*^{RNAi} discs using puromycin incorporation. We found that the rate protein synthesis was up-regulated in UPs but down-regulated in DCs upon *ND-42* inhibition (Fig S4). We further observed that the local increase in protein synthesis associated with the Second Mitotic Wave (SMW) was largely lost upon *ND-42* inhibition (consistent with the loss of the SMW itself, see Fig S1I). Thus, increased NADH/NAD$^+$ ratio appeared to correlate with reduced translation rate in DCs but not UPs, hence reflecting differences in the compensatory responses of these two cell populations to a common metabolic perturbation.
These results are discussed in the following paragraph:
Also consistent with a strong demand for energy in UPs upon cI inhibition, a strong antioxidant response and an increased rate of protein synthesis were observed in these proliferative cells. In contrast, differentiated cells showed a reduced rate of protein synthesis and a weaker antioxidant response. Thus, the minor difference in energy metabolism seen in wild-type discs between UPs and DCs appeared to be amplified upon loss of *ND-42* activity in the eye. We speculate that inhibition of the ETC activity drives metabolism away from its optimum, leading to inefficient energy consumption, possibly with more energy being spent at maintaining structures results (Sturm et al., 2023) and more energy being required for energetically costly processes, e.g. the synthesis of proteins with rapid turn-over. We further speculate that the increased rate of protein synthesis seen in *ND-42*^{RNAi} UPs might result from proteostasis defects associated with increased oxidative stress. In contrast, cells located posterior to the MF showed a reduced rate of protein synthesis, accompanied by a less dramatic shift towards glycolysis and antioxidant response, suggesting that these *ND-42*^{RNAi} DCs primarily responded to the loss of ETC by spending less energy to energetically costly processes, e.g. protein synthesis.

Second, we examined the possible roles of *Parp1* and *Sirt1* using RNAi. We found that Sirt1 inhibition led to a MF delay and that *Sirt1*^{RNAi} *ND-42*^{RNAi} conferred lethality (Fig 7J), suggesting that the activity of Sirt1 also contributes to constrain developmental speed, possibly downstream of NAD$^+$ metabolism. Also, while no MF delay was detected upon *Parp1*^{RNAi}, analysis of *Parp1*^{RNAi} *ND-42*^{RNAi} eye discs showed a stronger MF delay than the one measured in *ND-42*^{RNAi} discs (Fig 7J). This suggested that the levels of Parp1-dependent protein PARylation were limiting in *ND-42*^{RNAi} discs (note that decresead PARylation levels were measured by WB analysis in *ND-42*^{RNAi} eye discs, see Fig 7H,I). Whether and how the activities of Parp1 and Sirt1 constrain developmental speed due to reduced NAD$^+$ availability remain to be studied.

8. Why the MF delay induced during development has no consequence on adult eye morphology. What mechanisms compensate for this developmental defect?

Indeed, only minor defects were seen in adult eyes (Fig S1B,C). At least two mechanisms might explain why defects are minor. First, the progression of the MF could stop a few hours later in the dorsal (perturbed) compartment relative to the ventral (control) compartment. Second, hexagonal packing of the ommatidia is a late process which might re-organize ommatidia towards a crystal-like array. We did not experimentally address these interesting questions that, in our opinion, go beyond the scope of our work.

1st Revision - Editorial Decision **13th Apr 2026**

Dear Francois,

Thank you for submitting the revised version of your manuscript to The EMBO Journal. The study has now been seen by all original referees, who find that their main concerns have been addressed satisfactorily and now recommend acceptance of the manuscript.

There now remain only a few editorial and formatting points that need to be addressed before I can extend official acceptance of the manuscript:

1. As part of the EMBO Press transparent editorial process, The EMBO Journal will publish online a Peer Review File to accompany accepted manuscripts. This file will be published in conjunction with your paper and will include the anonymous referee reports, your point-by-point response and all pertinent correspondence relating to the manuscript, including decision letters. Please note that the Author Checklist will be published at the end of the Peer Review File.
Please let us know if you want to remove or not any figures or data from the Peer Review File prior to publication. Please note that retaining unpublished data in the Peer Review File means that these count as published and that the Peer Review File would need to be referenced in future publications.
2. Please check that the funding information is correct and identical both in the manuscript and our online system. Currently, grant NV DOC4 is listed in the submission system, but not in the manuscript.
3. The manuscript sections should be in the following order: Title page - Abstract - Introduction - Results - Discussion - Methods - Data Availability - Acknowledgments - Disclosure and Competing Interests Statement - References - Figure Legends - (Main Tables with legends if applicable) - Expanded View Figure Legends.
4. Please include EV figure legends in the manuscript text file, after main figure legends.
5. CRediT has replaced the traditional author contributions section because it offers a systematic, machine-readable author contributions format that allows for more effective research assessment. Please remove the Authors Contributions from the manuscript and use the free text boxes beneath each contributing author's name in our online submission system to add specific details on the author's contribution. More information is available in our guide to authors.
6. Please turn Tables EV1-EV4 into Datasets EV1-EV4 and upload in xlsx file format as Datasets. Please adjust the nomenclature, source file name and callouts to be adjusted to Dataset EV1-EV4 accordingly. The respective legends should be included within the corresponding dataset file in a separate sheet/tab.
7. Please provide source data for the main manuscript figures as requested on September 30th, 2025.
8. In our standard image check, we noted reuse of Western blot data between between Figure 7H and Figure EV 5D' - please check and correct if needed. If this was intentional, please indicate the reuse in the figure legend.
9. Our data editors have flagged the following issues in figure legends that need correcting:
- Please define the annotated p values ****/***/**/* as well as provide the exact p-values in the legend of figures EV 1i; EV5a as appropriate.
- Please provide the exact p values in the legends of figures 1f; 2d; 4c, f, i, j, j', k, k'; 5k, l, n'; 6a, g; 7a, b, d-f', i-m; EV 3d; EV 5c.
- Please indicate the statistical test used for data analysis in the legends of figures 7l, m; EV 1i; EV 2d, f; EV 5a.
- Please define the box plots in terms of minima, maxima, centre, bounds of box and whiskers, and percentile in the legends of figures 1f; 2d; 4c, f, i, j-j', k-k'; 5k, l, m', n'; 6a, g; 7a, b, d-f', i-m; EV 1i; EV 3d; EV 4b-b', d-d'; EV 5a, c.
- Please provide the information on the number and nature of replicates in the legends of figures 4j-j', k-k'; 5m', n'; 7i, k-m; EV 1d, i; EV 3d; EV 5c.
- Please define the error bars in the legends of figures 3c, d; 5d, d'; EV 1d''.
- Please provide a scale bar for figures EV 1b, c.
- Please define blue, orange dots in the legend of figure EV 1b, c.
- Please define the white dashed lines in the legends of figures 1b-b'; 6c, c'.
10. Papers published in The EMBO Journal are accompanied online by a 'Synopsis' to enhance discoverability of the manuscript. It consists of A) a short (1-2 sentences) summary of the findings and their significance, B) 3-4 bullet points highlighting key results and C) a synopsis image that is 550x300 pixels large (width x height, jpeg or png format). You can either show a model or key data in the synopsis image. Please note that the image size is rather small and that text needs to be readable at the final size. Please send us this information together with the revised manuscript.

Please let me know if you have any questions regarding any of these points. You can use the link below to upload the revised files.

Thank you again for giving us the chance to consider your manuscript for The EMBO Journal. I look forward to receiving the final version!

With best wishes,

Ieva

Ieva Gailite, PhD
Senior Scientific Editor
The EMBO Journal
Meyerhofstrasse 1
D-69117 Heidelberg
Tel: +4962218891309
i.gailite@embojournal.org

We realize that it is difficult to revise to a specific deadline. In the interest of protecting the conceptual advance provided by the work, we recommend a revision within 3 months (12th Jul 2026). Please discuss the revision progress ahead of this time with the editor if you require more time to complete the revisions. Use the link below to submit your revision:

https://emboj.msubmit.net/cgi-bin/main.plex

Referee #1:

The authors have addressed all majors points in their revision. I see no further point preventing publication.

Referee #2:

The authors have overall satisfactorily addressed my comments. I think they could have put a bit more effort into showing that the *order* (or sequence) - not just the timing - of neuoronal fate acquisition (equivalent to the temporal series in the fly medulla or the mammalian neocortex for example) is unaffected. I understand that the 30% effect on the timing may be too subtle to detect and I have no problem with not including the Prospero marker data, but this was not the question. Nevertheless, overall, I think this is an important piece of work and I support its publication in EMBO J.

Referee #3:

The authors have adequately addressed this reviewer's comments.

All editorial and formatting issues were resolved by the authors.

2nd Revision - Editorial Decision 24th Apr 2026

Dear Francois,

Thank you for incorporating the final editorial and formatting requests in the manuscript. I am now pleased to inform you that your manuscript has been accepted for publication. Congratulations with a nice study!

Before we forward your manuscript to our publishers, there are a couple of aspects that I would need your input on:
1. We would like to propose some edits in the manuscript abstract and synopsis. I have also written a short blurb that will accompany the title of your manuscript in our online table of contents. Please take a look at the proposed text changes in the attached text file and let me know if any corrections are needed.
2. The submitted synopsis image did not fit to the need dimensions of 550x300 pixels. I have selected a section of the image and resized it for this purpose - please see in the attachment and let me know if this looks acceptable to you.
3. We need the statement about the box plot features added to all figure legends featuring them, which we have added for you based on the template you included in the legend for Figure 1.

Your manuscript will be processed for publication by EMBO Press. It will be copy edited and you will receive page proofs prior to publication. Please note that you will be contacted by Springer Nature Author Services to complete licensing and payment information.

You may qualify for financial assistance for your publication charges - either via a Springer Nature fully open access agreement or an EMBO initiative. Check your eligibility: https://link.springer.com/journal/44318/how-to-publish-with-us

Should you be planning a Press Release on your article, please get in contact with embo_production@springernature.com as early as possible in order to coordinate publication and release dates.

If you have any questions, please do not hesitate to contact the Editorial Office or me directly. Thank you for this interesting contribution to The EMBO Journal!

With best wishes,

Ieva

Ieva Gailite, PhD
Senior Scientific Editor
The EMBO Journal
Meyerhofstrasse 1
D-69117 Heidelberg
Tel: +4962218891309
i.gailite@embojournal.org

*****
Please note that it is The EMBO Journal policy for the transcript of the editorial process (containing referee reports and your response letters) to be published as an online supplement to each paper. If you should prefer removal of any referee-only figures included in the point-by-point response(s), e.g. because they may still be used for future publication or because they have been reproduced from published work by others, please do let us know immediately via response email.
More information is available here: https://link.springer.com/partners/embo-press/editorial-policies#Peer%20review

** Click here to be directed to your login page: https://emboj.msubmit.net

EMBO Press Author Checklist

Corresponding Author Name: Francois Schweisguth	
Journal Submitted to: The EMBOJ Journal	
Manuscript Number: EMBOJ-2025-122331	

USEFUL LINKS FOR COMPLETING THIS FORM
The EMBO Journal - Author Guidelines
EMBO Reports - Author Guidelines
Molecular Systems Biology - Author Guidelines
EMBO Molecular Medicine - Author Guidelines

Reporting Checklist for Life Science Articles (updated January
This checklist is adapted from Materials Design Analysis Reporting (MDAR) Checklist for Authors. MDAR establishes a minimum set of requirements in transparent reporting in the life sciences (see Statement of Task: 10.31222/osf.io/9sm4x). Please follow the journal's guidelines in preparing your
Please note that a copy of this checklist will be published alongside your article.

Abridged guidelines for figures
1. Data
The data shown in figures should satisfy the following conditions:
- ➞ the data were obtained and processed according to the field's best practice and are presented to reflect the results of the experiments in an accurate and unbiased manner.
- ➞ ideally, figure panels should include only measurements that are directly comparable to each other and obtained with the same assay.
- ➞ plots include clearly labeled error bars for independent experiments and sample sizes. Unless justified, error bars should not be shown for technical
- ➞ if n<5, the individual data points from each experiment should be plotted. Any statistical test employed should be justified.
- ➞ Source Data should be included to report the data underlying figures according to the guidelines set out in the authorship guidelines on Data

2. Captions
Each figure caption should contain the following information, for each panel where they are relevant:
- ➞ a specification of the experimental system investigated (eg cell line, species name).
- ➞ the assay(s) and method(s) used to carry out the reported observations and measurements.
- ➞ an explicit mention of the biological and chemical entity(ies) that are being measured.
- ➞ an explicit mention of the biological and chemical entity(ies) that are altered/varied/perturbed in a controlled manner.
- ➞ the exact sample size (n) for each experimental group/condition, given as a number, not a range;
- ➞ a description of the sample collection allowing the reader to understand whether the samples represent technical or biological replicates (including how many animals, litters, cultures, etc.).
- ➞ a statement of how many times the experiment shown was independently replicated in the laboratory.
- ➞ definitions of statistical methods and measures:
  - common tests, such as t-test (please specify whether paired vs. unpaired), simple χ2 tests, Wilcoxon and Mann-Whitney tests, can be unambiguously identified by name only, but more complex techniques should be described in the methods section;
  - are tests one-sided or two-sided?
  - are there adjustments for multiple comparisons?
  - exact statistical test results, e.g., P values = x but not P values < x;
  - definition of 'center values' as median or average;
  - definition of error bars as s.d. or s.e.m.

Please complete ALL of the questions below.
Select "Not Applicable" only when the requested information is not relevant for your study.

Materials

Newly Created Materials	Information included in the manuscript?	In which section is the information available? (Reagents and Tools Table, Materials and Methods, Figures, Data Availability Section)
New materials and reagents need to be available; do any restrictions apply?	Yes	Materiels and Methods

Antibodies	Information included in the manuscript?	In which section is the information available? (Reagents and Tools Table, Materials and Methods, Figures, Data Availability Section)
For **antibodies** provide the following information:<br>- Commercial antibodies: RRID (if possible) or supplier name, catalogue number and or/clone number<br>- Non-commercial: RRID or citation	Yes	Materiels and Methods + Reagents and Tools Table

DNA and RNA sequences	Information included in the manuscript?	In which section is the information available? (Reagents and Tools Table, Materials and Methods, Figures, Data Availability Section)
**Short novel DNA or RNA including primers, probes**: provide the sequences.	Not Applicable	

Cell materials	Information included in the manuscript?	In which section is the information available? (Reagents and Tools Table, Materials and Methods, Figures, Data Availability Section)
**Cell lines:** Provide species information, strain. Provide accession number in repository **OR** supplier name, catalog number, clone number, and/**OR** RRID.	Not Applicable	
**Primary cultures:** Provide species, strain, sex of origin, genetic modification status.	Not Applicable	
Report if the cell lines were recently **authenticated** (e.g., by STR profiling) and tested for mycoplasma contamination.	Not Applicable	

Experimental animals	Information included in the manuscript?	In which section is the information available? (Reagents and Tools Table, Materials and Methods, Figures, Data Availability Section)
**Laboratory animals or Model organisms:** Provide species, strain, sex, age, genetic modification status. Provide accession number in repository **OR** supplier name, catalog number, clone number, **OR** RRID.	Yes	Materiels and Methods + Reagents and Tools Table
**Animal observed in or captured from the field:** Provide species, sex, and age where possible.	Not Applicable	
Please detail **housing and husbandry conditions**.	Yes	Materiels and Methods

Plants and microbes	Information included in the manuscript?	In which section is the information available? (Reagents and Tools Table, Materials and Methods, Figures, Data Availability Section)
**Plants:** provide species and strain, ecotype and cultivar where relevant, unique accession number if available, and source (including location for collected wild specimens).	Not Applicable	
**Microbes:** provide species and strain, unique accession number if available, and source.	Not Applicable	

Human research participants	Information included in the manuscript?	In which section is the information available? (Reagents and Tools Table, Materials and Methods, Figures, Data Availability Section)
If collected and within the bounds of privacy constraints report on age, sex and gender or ethnicity for all study participants.	Not Applicable	

Core facilities	Information included in the manuscript?	In which section is the information available? (Reagents and Tools Table, Materials and Methods, Figures, Data Availability Section)
If your work benefited from core facilities, was their service mentioned in the acknowledgments section?	Not Applicable	

Design

Study protocol	Information included in the manuscript?	In which section is the information available? (Reagents and Tools Table, Materials and Methods, Figures, Data Availability Section)
If study protocol has been **pre-registered, provide DOI in the manuscript**. For clinical trials, provide the trial registration number **OR** cite DOI.	Not Applicable	
Report the **clinical trial registration number** (at ClinicalTrials.gov or equivalent), where applicable.	Not Applicable	

Laboratory protocol	Information included in the manuscript?	In which section is the information available? (Reagents and Tools Table, Materials and Methods, Figures, Data Availability Section)
Provide DOI OR other citation details if **external detailed step-by-step protocols** are available.	Not Applicable	

Experimental study design and statistics	Information included in the manuscript?	In which section is the information available? (Reagents and Tools Table, Materials and Methods, Figures, Data Availability Section)
Include a statement about **sample size** estimate even if no statistical methods were used.	Yes	Materiels and Methods (design and statistics paragraph)
Were any steps taken to minimize the effects of subjective bias when allocating animals/samples to treatment (e.g. **randomization procedure**)? If yes, have they been described?	Not Applicable	
Include a statement about **blinding** even if no blinding was done.	Yes	Materiels and Methods (design and statistics paragraph)
Describe **inclusion/exclusion criteria** if samples or animals were excluded from the analysis. Were the criteria pre-established?<br><br>If sample or data points were omitted from analysis, report if this was due to attrition or intentional exclusion and provide justification.	Not Applicable	
For every figure, are **statistical tests** justified as appropriate? Do the data meet the assumptions of the tests (e.g., normal distribution)? Describe any methods used to assess it. Is there an estimate of variation within each group of data? Is the variance similar between the groups that are being statistically compared?	Yes	Figure legends

Sample definition and in-laboratory replication	Information included in the manuscript?	In which section is the information available? (Reagents and Tools Table, Materials and Methods, Figures, Data Availability Section)
In the figure legends: state number of times the experiment was **replicated** in laboratory.	Yes	Figures
In the figure legends: define whether data describe **technical or biological replicates**.	Yes	Only biological replicates, as indicated in the Methods (Design and statistcs paragraph)

Ethics

Ethics	Information included in the manuscript?	In which section is the information available? (Reagents and Tools Table, Materials and Methods, Figures, Data Availability Section)
Studies involving **human participants**: State details of **authority granting ethics approval** (IRB or equivalent committee(s), provide reference number for approval.	Not Applicable	
Studies involving **human participants**: Include a statement confirming that **informed consent** was obtained from all subjects and that the experiments conformed to the principles set out in the WMA Declaration of Helsinki and the Department of Health and Human Services Belmont Report.	Not Applicable	
Studies involving **human participants:** For publication of **patient photos**, include a statement confirming that consent to publish was obtained.	Not Applicable	
Studies involving experimental **animals**: State details of **authority granting ethics approval** (IRB or equivalent committee(s), provide reference number for approval. Include a statement of compliance with ethical regulations.	Not Applicable	
Studies involving **specimen and field samples:** State if relevant **permits** obtained, provide details of authority approving study; if none were required, explain why.	Not Applicable	

Dual Use Research of Concern (DURC)	Information included in the manuscript?	In which section is the information available? (Reagents and Tools Table, Materials and Methods, Figures, Data Availability Section)
Could your study fall under dual use research restrictions? Please check biosecurity documents and list of **select agents and toxins** (CDC): https://www.selectagents.gov/sat/list.htm	Not Applicable	
If you used a select agent, is the security level of the lab appropriate and reported in the manuscript?	Not Applicable	
If a study is subject to dual use research of concern regulations, is the name of the **authority granting approval and reference number** for the regulatory approval provided in the manuscript?	Not Applicable	

Reporting

The MDAR framework recommends adoption of discipline-specific guidelines, established and endorsed through community initiatives. Journals have their own policy about requiring specific guidelines and recommendations to complement MDAR.

Adherence to community standards	Information included in the manuscript?	In which section is the information available? (Reagents and Tools Table, Materials and Methods, Figures, Data Availability Section)
State if relevant guidelines or checklists (e.g., **ICMJE, MIBBI, ARRIVE, PRISMA**) have been followed or provided.	Not Applicable	
For **tumor marker prognostic studies,** we recommend that you follow the **REMARK** reporting guidelines (see link list at top right). See author guidelines, under 'Reporting Guidelines'. Please confirm you have followed these guidelines.	Not Applicable	
For **phase II and III randomized controlled trials**, please refer to the **CONSORT** flow diagram (see link list at top right) and submit the CONSORT checklist (see link list at top right) with your submission. See author guidelines, under 'Reporting Guidelines'. Please confirm you have submitted this list.	Not Applicable	

Data Availability

Data availability	Information included in the manuscript?	In which section is the information available? (Reagents and Tools Table, Materials and Methods, Figures, Data Availability Section)
Have **primary datasets** been deposited according to the journal's guidelines (see 'Data Deposition' section) and the respective accession numbers provided in the Data Availability Section?	Yes	Data availability
Were **human clinical and genomic datasets** deposited in a public access-controlled repository in accordance to ethical obligations to the patients and to the applicable consent agreement?	Not Applicable	
Are **computational models** that are central and integral to a study available without restrictions in a machine-readable form? Were the relevant accession numbers or links provided?	Not Applicable	
If publicly available data were reused, provide the respective **data citations in the reference list**.	Not Applicable	
